# Conjugation of squalene to gemcitabine as unique approach exploiting endogenous lipoproteins for drug delivery

**DOI:** 10.1038/ncomms15678

**Published:** 2017-05-30

**Authors:** Dunja Sobot, Simona Mura, Semen O. Yesylevskyy, Laura Dalbin, Fanny Cayre, Guillaume Bort, Julie Mougin, Didier Desmaële, Sinda Lepetre-Mouelhi, Grégory Pieters, Bohdan Andreiuk, Andrey S. Klymchenko, Jean-Louis Paul, Christophe Ramseyer, Patrick Couvreur

**Affiliations:** 1 Institut Galien Paris-Sud, UMR 8612, CNRS, Univ Paris-Sud, Université Paris-Saclay, Faculté de Pharmacie, 5 rue Jean-Baptiste Clément, F-92296 Châtenay-Malabry Cedex, France; 2 Department of Physics of Biological Systems, Institute of Physics of the National Academy of Sciences of Ukraine, Prospect Nauky 46, 03028 Kyiv, Ukraine; 3 CEA Saclay, iBiTecS-S/SCBM, Labex LERMIT, 91191 Gif-sur-Yvette, France; 4 Laboratoire de Biophotonique et Pharmacologie, UMR CNRS 7213, University of Strasbourg, 74 route du Rhin, 67401 Illkirch Cedex, France; 5 Department of Organic Chemistry, Chemistry Faculty, Taras Shevchenko National University of Kyiv, 01601 Kyiv, Ukraine; 6 AP-HP, Hôpital Européen Georges Pompidou, Service de Biochimie, 75015 Paris, France; 7 Lip(Sys)^2^, Athérosclérose: homéostasie et trafic du cholestérol des macrophages, Univ Paris-Sud, Université Paris-Saclay, 92296 Châtenay-Malabry, France; 8 Laboratoire Chrono Environnement UMR CNRS 6249, Université de Bourgogne Franche-Comté, 16 route de Gray, 25030 Besançon Cedex, France

## Abstract

Once introduced in the organism, the interaction of nanoparticles with various biomolecules strongly impacts their fate. Here we show that nanoparticles made of the squalene derivative of gemcitabine (SQGem) interact with lipoproteins (LPs), indirectly enabling the targeting of cancer cells with high LP receptors expression. *In vitro* and *in vivo* experiments reveal preeminent affinity of the squalene-gemcitabine bioconjugates towards LP particles with the highest cholesterol content and *in silico* simulations further display their incorporation into the hydrophobic core of LPs. To the best of our knowledge, the use of squalene to induce drug insertion into LPs for indirect cancer cell targeting is a novel concept in drug delivery. Interestingly, not only SQGem but also other squalene derivatives interact similarly with lipoproteins while such interaction is not observed with liposomes. The conjugation to squalene represents a versatile platform that would enable efficient drug delivery by simply exploiting endogenous lipoproteins.

## Abstract (web summary)

The interaction of nanoparticles with a range of biomolecules once they have been injected within the body can affect their performance. Here, the authors demonstrate that squalene nanomaterials conjugated with anticancer drugs can interact with lipoproteins and can be used to target cancer cells.

Gemcitabine (Gem) is a nucleoside analogue widely used in clinical practice for the treatment of various solid tumours^1^,^2^. However, the anticancer activity of gemcitabine is hampered by serious limitations such as short biological half-life due to rapid blood metabolization, intracellular diffusion, which is restricted to the expression of the nucleoside transporter hENT1 and emergence of various mechanisms of resistance^3^,^4^,^5^. Consequently, developing improved gemcitabine formulations is an important challenge in cancer drug discovery. In this context, we have developed an original approach relying on the introduction of squalene (SQ), a natural triterpene and precursor of the cholesterol's biosynthesis, as a biocompatible material for drug delivery purposes. Squalene has been used as a building block for the synthesis of SQ-drug bioconjugates, which demonstrated the ability to self-assemble in aqueous medium in the form of nanoparticles (NPs), without the need of any other transporter material^6^,^7^,^8^. The bioconjugates obtained by the covalent linkage of SQ to gemcitabine (SQGem) resulted in the spontaneous formation of NPs in water, with a diameter of ∼100–200 nm and high drug loading (∼40%)^9^. Preclinical evaluation of these NPs revealed reduced blood clearance and metabolization after intravenous administration and greater *in vitro* and *in vivo* anticancer activity, compared to free Gem, against both solid subcutaneously grafted tumours and aggressive metastatic leukaemia^10^,^11^,^12^. It has been also reported that SQGem NPs can interact with cellular membranes^13^,^14^. But, in the absence of any specific ligand, how these NPs could accumulate into cancer cells remained totally unexplored. Therefore, despite promising results, the introduction of this therapeutic concept into clinical trials has been hindered by a lack of knowledge concerning the exact mechanism behind tumour recognition and anticancer activity of SQGem NPs. Accordingly, current efforts need to be focused on the identification and elucidation of such mechanism of action.

Once introduced in the organism, NPs encounter a complex biological environment composed of a plethora of endogenous molecules. In function of the composition of the surrounding biological environment (depending on the administration route), as well as the NP physico-chemical properties (for example, material, size, surface charge and functionalization), NPs immediately interact with a specific set of biomolecules, thereby acquiring certain biological identity^15^,^16^,^17^,^18^. This identity will govern the *in vivo* fate of NPs in terms of biodistribution, pharmacokinetics, therapeutic efficacy and potential toxicity^19^,^20^,^21^. The interacting biomolecules might hinder the NPs recognition by the targeted cells or, on the contrary, increase the specific interaction of the NPs with the corresponding biological target^19^,^22^,^23^.

Among proteins capable of interacting with NPs, apolipoproteins have received special attention^20^,^24^,^25^,^26^,^27^. Apolipoproteins are amphipathic molecules, which associate with different plasma lipids to form complex structures called lipoproteins (LPs), acting as macromolecular vehicles of water-insoluble lipids in the circulation (for example, cholesterol, triglycerides and so on)^28^. LPs display various structures and functions and according to their ultracentrifugation flotation density and electrophoretic mobility, they can be classified into chylomicrons, very low density LPs (VLDL), low density LPs (LDL) and high density LPs (HDL). Apolipoproteins play a major role in determining LPs structural stability, metabolism, as well as interaction with cells since they act as ligands for LP receptors^29^,^30^.

Despite observing the presence of these apolipoproteins at the NP surface after intravenous administration, only few studies investigated the interactions of NPs with blood lipid components or with the LP particles as a whole^31^,^32^ and examined the relevance of such interactions for interpreting the *in vivo* fate of NPs. The association to LPs in the bloodstream has been largely described for many hydrophobic drugs and it is well known that it can have a strong impact on the drug disposition and biological activity. Accordingly, since 2002, the US Food and Drug Administration (FDA) has recommended the introduction of LP-drug distribution studies for every novel drug with hydrophobic character^33^. In addition, LPs have been described as excellent carriers for targeted delivery of various drugs and imaging agents, due to their endogenous, non-toxic, long-circulating nature and to their ability to be recognized and taken up via the LP receptors^34^,^35^,^36^. However, the development of LPs for drug delivery has been hampered by the difficulty to prepare reproducible batches of drug loaded endogenous LPs^37^.

The capacity of LPs to transport hydrophobic molecules, the lipid nature of squalene as well as its bio-relation to cholesterol altogether led us to believe that the interaction between the SQGem NPs and LPs deserved to be deeply explored. In the present study, we demonstrate that SQGem bioconjugates intravenously administered as NPs could be captured and hence transported by plasma LPs, in particular via cholesterol-rich ones, both *in vitro* in human blood and *in vivo* in rodents. We have also analysed the ability of SQGem to spontaneously interact with LDL on a molecular level. We discover that endogenous LDL particles may function as carriers for SQGem molecules, thus allowing the indirect targeting of cancer cells displaying high expression and activity of LDL receptors (LDLR), without the need to functionalize NPs surface with specific ligands.

## Results

### *In vitro* distribution of SQGem and Gem in human plasma

**Figure 1:**
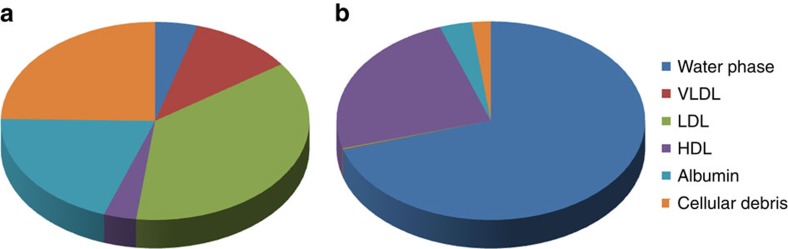
^3^H-SQGem and ^3^H-Gem plasmatic distribution *in vitro*. The distribution of ^3^H among the different plasma fractions was analysed after incubation (5 min) of (**a**) ^3^H-SQGem NPs or (**b**) ^3^H-Gem with human blood. Results are expressed as a percentage (mean values) of total plasma radioactivity.

The interaction of SQGem NPs (Supplementary Fig. 1, Supplementary Table 1) with the different plasma fractions was first investigated *in vitro* after incubation with human blood samples at 37 °C for 5 min. To track the distribution of SQGem in the different fractions, radiolabelled ^3^H-SQGem NPs were prepared and the radioactive signal was monitored. After blood centrifugation and plasma separation from the blood cells, it was found that most of ^3^H-SQGem remained in the plasma (∼80%; Supplementary Fig. 4), which was then analysed for ^3^H signal into the LP fractions (that is, VLDL, LDL and HDL), the albumin fraction and the water phase. The latter corresponded to unbound ^3^H-SQGem. To be noted that after the ultracentrifugation step, a small pellet was observed at the bottom of the ultracentrifugation tube, which was attributed to cellular debris still present in the plasma. The results expressed as a percentage of the overall plasma signal revealed that, once in contact with human blood, ^3^H-SQGem was mainly distributed between LP fractions (51%) and albumin (20%) with some amount associated to the cellular debris (25%) and a very small quantity present in the water phase (4%) (Fig. 1a). Remarkably, among the LP fractions, the LDL was the predominant ^3^H-SQGem-interacting LP (37%) followed by VLDL (11%) and HDL (3%). On the other hand, free ^3^H-Gem was almost entirely recovered in the water phase (70%), even if some radioactivity was also measured in HDL (24%). Interaction of ^3^H-Gem with albumin and LDL was almost negligible (3% and below 1%, respectively; Fig. 1b). The ^3^H-SQGem NPs and ^3^H-Gem distribution among the different plasma fractions was further tested after longer blood incubation times (*t*=15, 30, 60, 90 and 120 min) but the distribution profile remained unchanged overtime for both formulations (Supplementary Fig. 5).

**Figure 2:**
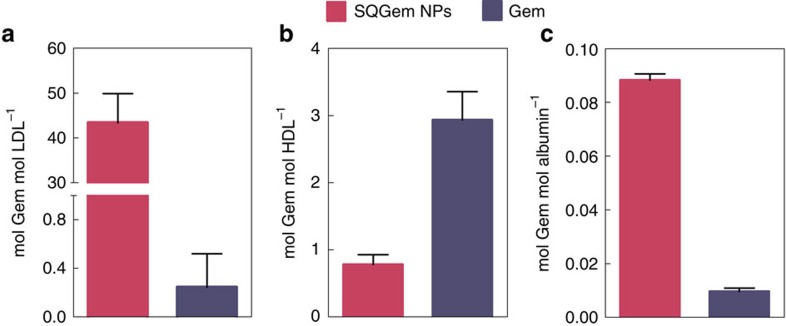
Molar ratios between SQGem or Gem and plasmatic particles. Molar concentration of SQGem (magenta bars) and Gem (blue bars) per mole of (**a**) LDL, (**b**) HDL and (**c**) albumin after incubation with total human blood (5 min). Bars represent mean±s.e.m.

The molar concentration of ^3^H-SQGem found in each fraction was then related to the molar concentration of albumin, LDL and HDL in each patient's blood sample (Fig. 2). Results revealed a dramatic affinity of ^3^H-SQGem for LDL (that is, 43 moles of Gem per mole of LDL), while more than a 50-fold lower affinity was observed towards HDL and albumin. The very low molar ratios of Gem confirmed the negligible interaction of the free drug with plasma components.

### Isothermal titration calorimetry analysis

The specificity of the interaction between SQGem and LPs was further investigated by isothermal titration calorimetry (ITC) experiments performed using human LPs separated from the blood of healthy volunteers. The heat flows were determined when SQGem NPs were added to LDL dispersion, HDL dispersion or albumin solution placed in the titration cell. ITC thermograms revealed the existence of a strong interaction between the SQGem bioconjugate and the LDL, while such interaction was not observed either with HDL or with albumin. Specificity of the recorded signal was confirmed after dilution of SQGem NPs in PBS (Supplementary Fig. 8). These results confirmed the establishment of a specific interaction of SQGem with the main cholesterol transporters (that is, LDL) in human blood.

### *In silico* modelling

**Figure 3:**
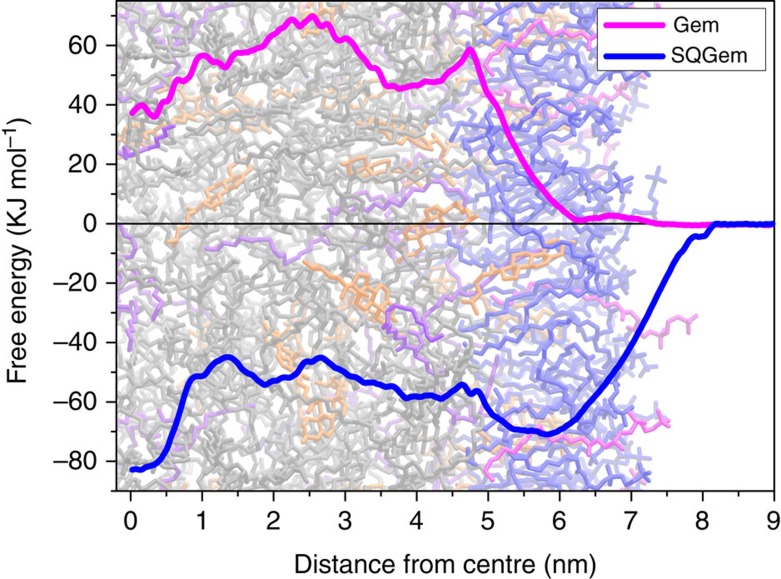
Mean force potentials. Potentials of mean force of transferring individual Gem (magenta line) and SQGem (blue line) molecules from bulk water to the lipid core of model LDL particle. The plots are superimposed onto a snapshot of the equilibrated LDL system. 1-palmitoyl-2-oleoyl-sn-glycero-3-phosphocholine (POPC) lipids are shown in blue, 1-palmitoyl -2-hydroxy-sn-glycero-3-phosphocholine (lyso PC) in red, cholesterol in orange, cholesterol oleate in grey and glyceryl trioleate in violet.

Atomic details of the interaction of LDL with single SQGem and Gem molecules were obtained by means of molecular dynamics simulations. A simplified atomistic model, which reproduces the hydrophobic properties of the lipid cores of LDL particles, was used (Supplementary Figs 18 and 19). We computed the potential energy profiles of mean force of transferring Gem and SQGem molecules from bulk water to the lipid core of LDL particle (Fig. 3). It was clearly observed that interaction of Gem with LDL was energetically unfavourable while SQGem had strong affinity towards LDL and accumulated in their hydrophobic core.

### *In vivo* distribution of SQGem and Gem in rat plasma

**Figure 4:**
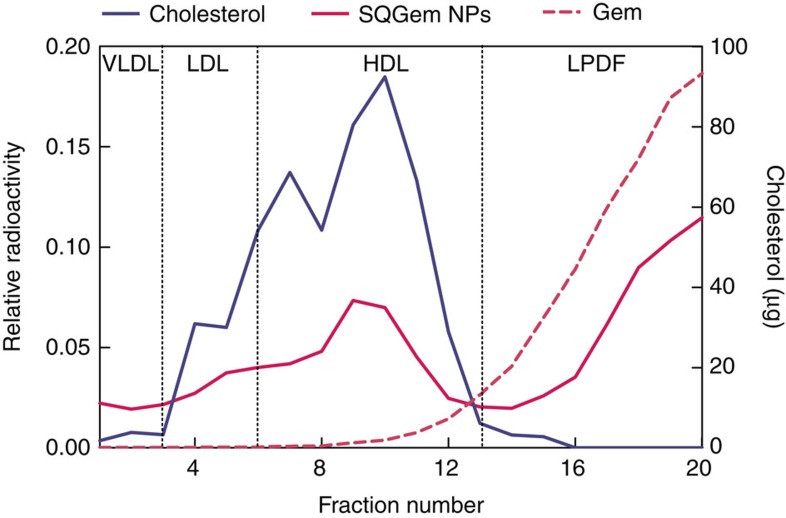
^3^H-SQGem and ^3^H-Gem plasmatic distribution *in vivo*. Radioactivity (magenta lines) and cholesterol (blue line) distribution among the collected fractions of plasma obtained from rats treated with ^3^H-SQGem (solid magenta line) or free ^3^H-Gem (dashed magenta line), 5 min post administration. Results are expressed as relative radioactivity (mean values) compared to total plasma.

The distribution of SQGem between the different plasma fractions was then investigated after intravenous injection of ^3^H-SQGem NPs to rats with plasma collection 5 min post administration. Twenty fractions (200 μl each), from the top to the bottom of the tube, were separated on the basis of their hydrated density by NaBr gradient ultracentrifugation method. According to the measured density and the electrophoretic mobility, fractions were then attributed to the different LP classes, that is, VLDL (fractions 1–3), LDL (fractions 4–6), HDL (fractions 7–13) or LP-deficient fraction (LPDF; fractions 14–20), which contained only water soluble proteins (Supplementary Fig. 6). Plasma albumin (62 mg) was recovered in fractions 16–20. The radioactivity was determined in each fraction and expressed as relative to the radioactivity in the whole plasma. Figure 4 shows that the ^3^H-SQGem mainly distributed between LP (49%) and LPDF fractions (51%). Among the LPs, the highest percentage of ^3^H-SQGem was found in HDL fractions (32%), followed by LDL (10%) and VLDL (6%). The cholesterol used as a marker was completely recovered (99%) in the LP fractions (Fig. 4). To be noted that the HDL fraction was the most abundant in cholesterol (75%) and displayed two peaks corresponding to two distinct HDL subpopulations.

The same set of experiments was carried out after injection of free ^3^H-Gem to rats, highlighting the absence of interaction of the drug with LPs. Indeed, the totality of ^3^H was recovered in the LPDF fraction (Fig. 4), likely as protein-unbound since Gem was previously shown to have negligible interaction with plasma proteins after intravenous administration^38^. This has been verified after incubation of ^3^H-Gem with NaBr solutions of various densities without plasma proteins. Whatever the density of the solution in which the ^3^H-Gem was placed before ultracentrifugation, its position remained unchanged thus indicating that the observed repartition reflected the very own physico-chemical behaviour of Gem during ultracentrifugation rather than any interaction with plasma proteins (Supplementary Fig. 7).

### *In vivo* distribution of other bioconjugates and liposomes

With the aim of generalizing the proposed approach, we have investigated the behaviour of NPs made by the self-assembly of two other squalene derivatives: ^3^H-Squalene-adenosine (^3^H-SQAd) NPs and Squalene-Cyanine 5.5 (SQCy5.5) NPs. (Supplementary Figs 2 and 3, Supplementary Table 1) The interaction of these two structurally different squalene derivatives with LPs was determined by using the same experimental setting previously established for SQGem NPs. Thus, ^3^H-SQAd NPs and SQCy5.5 NPs were intravenously injected to rats and their distribution among plasma fractions was assessed.

First, it was observed that the distribution profile of ^3^H-SQAd NPs perfectly overlapped the one already observed after the administration of SQGem NPs, and the highest percentage of ^3^H-SQAd was found in the HDL fraction (63%), followed by the LDL (15%) and the VLDL (7%) fractions (Supplementary Fig. 9). Only 15% of the ^3^H-SQAd was distributed in the LPDF fractions. Similarly to the free gemcitabine, the free adenosine did not interact significantly with the LPs and was mainly recovered at the bottom of the tube, in the LPDF. As already observed with free gemcitabine (Supplementary Fig. 7), this localization could be attributed to the physico-chemical properties of the molecule, rather than to interactions with soluble plasma proteins.

Second, the administration of SQCy5.5 NPs also resulted in comparable distribution among the plasma fractions, thus confirming that the conjugation to squalene led to a specific interaction with LPs. In this case, due to detection limits, quantification has been carried out in the pool of fractions corresponding to LDL/VLDL (fractions 1–6), HDL (fractions 7–13) and LPDF (fractions 14–20). The highest % of SQCy5.5 (expressed as relative to the concentration in the whole plasma) has been found again in the HDL fractions (50%) (Supplementary Fig. 10).

We additionally investigated whether such a specific interaction with the major cholesterol-transporting particles in rats could also occur (or not) with other types of nanocarriers. Thus, ^3^H-gemcitabine-loaded liposomes (^3^H-LipoGem) were prepared and they were administered intravenously to rats. Following the same experimental protocol as already described for ^3^H-SQGem NPs, ^3^H-SQAd NPs and SQCy5.5 NPs, blood was collected by cardiac puncture 5 min after injection and the different plasma fractions were separated, according to the density gradient ultracentrifugation. The majority of the radioactivity, expressed as relative to the total amount in plasma, was found in the fractions 5–8, which could *a priori* correspond to the HDL1 subpopulation, also suggesting an interaction of liposomes with this subpopulation.

However, very importantly, a control experiment carried out by replacing the plasma with a 1.25 g ml^−1^ NaBr solution incubated for 5 min with ^3^H-GemLipo, revealed that ^3^H-GemLipo accumulated in the same 5–8 fractions even in the absence of any LP. In addition, it was observed that, contrarily to the different squalene derivatives, the LipoGem only partly overlapped with the cholesterol content in the different fractions. Accordingly, it was concluded that the distribution of ^3^H-GemLipo in fractions 5–8 simply resulted from their accumulation in the region of the gradient which displayed the same density of the liposomal formulation and was not a consequence of the interaction with the LPs (Supplementary Fig. 11).

### FRET NPs stability in blood

Stability of SQGem NPs was investigated *in vitro* using FRET SQGem NPs (Supplementary Table 1) to give insight about the effect of blood on NP integrity. SQGem NPs were labelled with the squalene derivatives of the cyanine 5.5 (SQCy5.5, 0.6% w/w) and cyanine 7.5 (SQCy7.5, 0.6% w/w; Supplementary Fig. 3), which behaved as FRET donor and acceptor, respectively. Fluorescence emission spectrum of FRET NPs is reported in Supplementary Fig. 12. Taking advantage of the dependence of FRET signal on the distance between the FRET pair (SQCy5.5/SQCy7.5) this approach enabled to monitor the integrity of SQGem NPs over time. Thus, NPs stability was assessed at 37 °C after opportune dilution in water, rat blood or ethanol, a solvent in which SQGem is soluble. Thus, the dilution in ethanol was used as a control of the complete disassembly of FRET SQGem NPs and consequently, the absence of energy transfer between the donor and acceptor dyes. While NPs were stable in water up to 24 h, the rapid drop of the FRET signal in the rat blood clearly indicated a fast disassembly of the NPs in this medium (Supplementary Fig. 13), resulting in the release of individual SQGem molecules able to insert into LPs, probably as a result of the bio-relation between the squalene moiety and the cholesterol.

### SQGem cellular uptake

**Figure 5:**
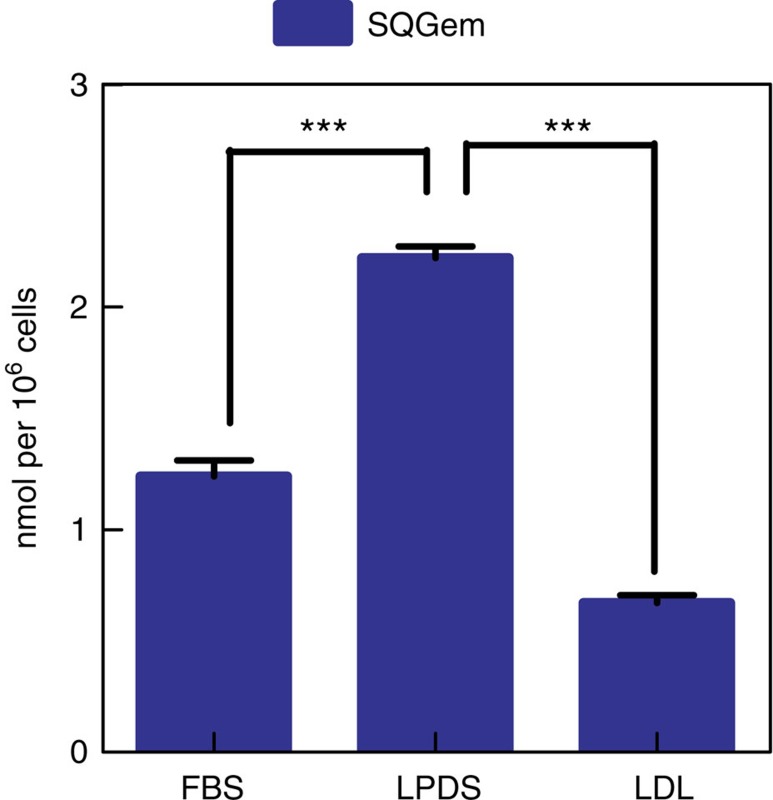
^3^H-SQGem NPs cellular uptake in MDA-MB-231 cells. ^3^H-SQGem NPs uptake in MDA-MB-231 cells cultured in medium supplemented with FBS, LPDS or preincubated with an excess of LDL in LPDS-supplemented medium at 37 °C. Results are expressed as nanomoles of Gem per one million of cells (bars represents mean values±s.e.m. ***P<0.001 by analysis of variance, Tukey's multiple comparison test).

The ability of LDL particles to mediate ^3^H-SQGem uptake via LDLR was investigated on a human breast cancer cell line (MDA-MB-231) with high LDLR expression (Supplementary Fig. 15). When MDA-MB-231 cells were cultivated in the absence of LPs (that is, medium supplemented with serum deficient in LPs, LPDS), an increased uptake of fluorescently labelled LDL (LDL-f) was observed, as compared to cells cultured in the presence of complete serum (fetal bovine serum (FBS)) (Supplementary Fig. 16), which was attributed to an increase in LDLR expression in these experimental conditions^39^. On the contrary, LDLR-mediated endocytosis of LDL-f was strongly inhibited when cells were preincubated with an excess of LDL. Interestingly, cell starvation with LPDS (serum deficient in LPs) significantly increased ^3^H-SQGem NPs cell uptake, whereas after pre-incubation with an excess of LDL more than threefold reduction in intracellular radioactivity signal was observed (Fig. 5).

The contribution of the LDLR has been further investigated by comparing the uptake of ^3^H-SQGem NPs on MDA-MB-231 and MCF-7 breast cancer cells, which display high and low level of LDLR expression, respectively (Supplementary Fig. 15). A higher radioactivity signal was detected in MDA-MB-231 cells compared to MCF-7 after 6 h of incubation with ^3^H-SQGem NPs at 37 °C (Supplementary Fig. 20).

### Tumour uptake of ^3^H-SQGem NPs *in vivo*

We then investigated the ability of LDL particles to transport and deliver ^3^H-SQGem to subcutaneously grafted tumours expressing different levels of LDL receptors. To make the proof of concept and to increase the amount of circulating LDL, the mice received diet with high cholesterol content (2%). After 4 weeks, cholesterol enriched diet led to a 1.6-fold increase in the LDL cholesterol level compared to a standard (cholesterol<0.3%) chow diet (0.72 versus 0.44 mmol l^−1^). Two human breast cancer cell lines with high (MDA-MB-231) and low (MCF-7) LDLR expression (Supplementary Fig. 15) were used to develop xenograft tumours in mice. At 6 h post administration of a single dose of ^3^H-SQGem NPs, the percentage of radioactivity in the tumour was twofold higher in tumour xenografts originating from MDA-MB-231 (high level of LDLR) cells than in xenografts originating from MCF-7 (low level of LDLR) (Supplementary Fig. 17).

## Discussion

This study clearly shows that the linkage of squalene to the anticancer agent gemcitabine enables a spontaneous strong interaction of SQGem molecules with blood plasma LPs which are therefore becoming a kind of endogenous carrier of this bioconjugate into the bloodstream. Altogether, the results of *in vitro* studies with human blood and *in vitro* ITC analysis, *in silico* computational studies, as well as *in vivo* studies with rodents strongly support this discovery.

The *in vitro* data with human blood showed that LDL were the major SQGem-interacting LP particles (Fig. 1a), while the ITC thermograms revealed the specificity of this interaction. The observed association with serum albumin was in accordance to previous reports about the importance of proteins in SQGem cellular uptake^40^. However, it has to be noted that albumin is the most abundant protein in human plasma and consequently, its molar concentration in plasma is very high in comparison to that of LDL particles (∼600 μM versus ∼1.3 μM)^41^,^42^. Hence, the quantitative distribution of ^3^H-SQGem among plasmatic components, when expressed as a single percentage value in each fraction, does not necessarily reflect the real affinity towards proteins and/or LPs. More relevant information has been obtained taking into account the molar concentration of albumin and LPs in each blood sample used in this study. Indeed, after incubation of ^3^H-SQGem NPs with human blood, molar ratio values demonstrated a 500-fold higher affinity of the bioconjugate for LDL in comparison to albumin (Fig. 2), thereby emphasizing the importance of LDL as SQGem carrier in the bloodstream. Moreover, when LDL and albumin were present at the same concentration (ITC experiments) no specific interaction between SQGem and albumin was measured (Supplementary Fig. 8). LDL particles contain two thirds of plasma cholesterol and they are the main carriers of cholesterol to peripheral tissues in humans^43^. Thus, the observed interaction clearly supported our initial hypothesis about similar behaviour of squalene bioconjugate and cholesterol.

The interaction of SQGem NPs with LPs might be imagined as a dynamic process which first involves the adsorption of the small LDLs (22 nm)^44^ at the NPs surface as soon as they enter into contact. This event was clearly observed in TEM images (Supplementary Fig. 14) where shape-modified SQGem NPs, possibly already in the course of a disassembly process (yellow arrows), and LDL particles (pink arrows) are visible but also SQGem NPs clearly displaying LPs on their surface (blue arrows). Then, the fast disassembly of the NPs as shown by FRET experiments (Supplementary Fig. 13) leads to the release of the SQGem bioconjugates, which will further insert into the LPs.

Of note, *in silico* simulations not only confirmed the affinity of SQGem for LDL but also showed that the transfer of individual SQGem molecules from water to the interior of LDL lipid droplet was strongly energetically favourable (Fig. 3). There is a deep energy well (60–80 kJ mol^−1^) which begins at the level of the outer layer of phospholipids and extends up to the centre of the lipid droplet and there is no energy barrier for SQGem to enter the LDL particle. This means that SQGem molecules can seamlessly diffuse into LDL, effectively accumulate inside and be then transported in the circulation.

On the contrary, after incubation with human blood, free gemcitabine was mainly recovered in the water phase, without association with LDL or any other plasma protein (Fig. 1b). This behaviour was confirmed by *in silico* modelling convincingly showing that free Gem molecules did not even bind to the surface of LDL lipid droplet (Fig. 3). Indeed, no energy well was observed at the level of the outer phospholipid layer and a high energy barrier (∼60 kJ mol^−1^) prevented Gem molecules from penetration into the LDL particle. Such a drastic difference concerning the behaviour of Gem and SQGem molecules, which was in perfect agreement between experiments and simulations, corroborates the idea that the SQGem-LDL interaction was mediated through the squalene moiety.

The interaction of ^3^H-SQGem with plasma components was also investigated *in vivo* after intravenous injection of ^3^H-SQGem NPs in rats. There are important qualitative and quantitative differences in the LP system of rodents and humans, in particular with HDL being more abundant and LDL being almost depleted in rats comparatively to humans. The LP separation method, normally used in clinical setting and here applied in the experiments performed with human blood samples, was not directly transposable to the blood collected from rats^45^,^46^. Hence, a modified single-step procedure using the technique of ultracentrifugation on a sodium bromide (NaBr) gradient was employed. In this set of experiments, a strong association of ^3^H-SQGem with LPs was observed once again but, unlike in human blood, the main SQGem-interacting LP in rats was HDL rather than LDL (Fig. 4). As mentioned above, rodents are known to differ from humans in their LP metabolism, which causes differences at the level of certain LP classes. In rats, the lack of cholesteryl ester transfer from HDL to VLDL and LDL results in very poor LDL population and a large HDL_1_ and HDL_2_ subpopulations especially enriched with cholesterol^46^. Accordingly, HDL particles acquire the role of cholesterol transporter in rodents' circulation. This was confirmed by the dosage of cholesterol performed in all rat plasma fractions which revealed two peaks that corresponded to the two HDL subpopulations (HDL_1_ and HDL_2_) with highest cholesterol accumulation (Fig. 4). Interestingly, ^3^H-SQGem repartition among the LP fractions perfectly followed the cholesterol profile with the highest amount of radioactivity found in the HDL fractions. Even though humans and rats differ in the main SQGem-interacting LP particles (LDL in human and HDL in rat), in both cases the observed repartition was consistent with their cholesterol content, thereby confirming the association of SQGem with endogenous cholesterol-transporting particles. This observation was also in accordance with previous reports using Langmuir blodget and showing accelerated penetration of SQGem into lipid monolayers when containing cholesterol, due to attractive interactions between these two molecules^13^. To be noted that SQGem incorporation into the lipid core of LP particles is unlikely to be triggered by the apoprotein component, since LDL and HDL have different protein compositions.

After injection of ^3^H-Gem in rats, absence of radioactivity in LPs was observed, confirming, once again, that in the case of SQGem, the interaction with LPs was mediated through the SQ moiety (Fig. 4). As already mentioned before, the presence of ^3^H-Gem in the LPDF fraction is explainable by the fact that, in the absence of interactions with plasma components, low molecular weight compounds such as gemcitabine, are not distributed along the different density fractions during the ultracentrifugation but simply remain at the deposition site (Supplementary Fig. 7). In addition, the lack of gemcitabine interaction with proteins after intravenous administration is well described in literature^38^.

Noteworthy is that other squalene derivatives (SQAd, SQCy5.5) were also observed to be capable to associate with LPs (Supplementary Figs 9 and 10), evidencing the key role of the SQ moiety, that being bio-similar to cholesterol, was driving the interaction with the main cholesterol-transporting particles in the circulation. On the contrary, when tested in the same experimental conditions, gemcitabine-loaded liposomes did not display any specific interaction with LPs (Supplementary Fig. 11), thus highlighting that the lipid nature of the carrier was not enough to ensure the establishment of a specific interaction.

Since the LDL endocytic uptake was proved to be highly increased in rapidly growing malignant cancer cells^47^, the incorporation of various anticancer drugs in endogenous LDL carriers has been proposed as a strategy for cancer cell targeting^35^. Despite initially promising results, the complexity of LDL isolation from fresh donor plasma, the storage concerns (that is, aggregation and degradation processes), as well as the need for efficient drug incorporation strategies, have significantly hampered their industrial development. As an alternative, synthetic recombinant LDL-like particles have been proposed^34^. However, also in this case, several challenges need to be faced. The main one consists in synthesizing endogenous-like particles, closely mimicking their native counterparts and capable of interacting with the LDL receptors. Since the apoB100 protein is responsible for binding and interaction with the LDLR, it is essential to have this component in functional recombinant LDL-like particles. However, apoB100 is a large size protein commercially available neither in recombinant nor in purified form, thereby complicating the production of these LDL-like particles.

In contrast to this challenging panorama, the interaction between SQGem and the entire endogenous LDL occurs spontaneously, without an additional need for complex LDL isolation, recombinant LDL synthesis and/or drug incorporation. LPs might therefore act as a natural endogenous carrier that transports SQGem towards the targeted site of action, thus enabling the accumulation in cells characterized by high LDL receptors activity.

Hence, we have investigated *in vitro* the ability of LDL particles to mediate the SQGem cellular uptake via the LDLR by tuning their expression and activity at the surface of MDA-MB-231 cancer cells. The activity of LDLR is normally regulated by the cellular demand for cholesterol^39^, thus an increased expression of the receptor may be observed in cholesterol-deprived cells cultivated in LPDS-containing medium whereas, on the contrary, the LDLR-mediated uptake would be blocked by an oversaturation with LDL. These conditions were confirmed by tracking the cellular uptake of fluorescently labelled LDL (Supplementary Fig. 16). Interestingly, it was observed that the cellular uptake of ^3^H-SQGem was highly dependent on the LDLR activity (Fig. 5). The higher uptake of ^3^H-SQGem in the MDA-MB-231 cells (high LDLR expression) compared to the MCF-7 (low LDLR expression) further confirmed the key role of this receptor in the cellular internalization of the SQGem bioconjugates (Supplementary Fig. 20). Similar observation was also done *in vivo*, when increased uptake of ^3^H-SQGem occurred in tumour xenografts originating from the breast cancer cell line with the highest expression of LDLR (Supplementary Fig. 17).

In conclusion, we show here that the single chemical linkage of squalene to gemcitabine allows incorporation into LDL after intravenous administration, which indirectly confers targeting capacity towards LDLR expressing cells. To the best of our knowledge, using squalene to drive a drug insertion into LDL and subsequent targeting represents a novel concept in drug delivery. In contrast to the more and more sophisticated and complicated drug delivery nanodevices described in the recent literature, our approach simply exploits the endogenous LPs as an indirect natural carrier. Although, the conjugation to squalene has been already successfully applied to a variety of molecules with different therapeutic indications (that is, anticancer agents^10^,^48^,^49^,^50^,^51^,^52^,^53^, neuroprotective molecules^54^, antibiotics^55^ and siRNA^56^), here for the first time, we have demonstrated that when conjugated to SQ these molecules are capable of interacting with the LPs. Accordingly, this approach represents a flexible and versatile platform for drug delivery via LPs.

## Methods

### ^3^H-SQGem nanoparticles formulation and characterization

^3^H-SQGem NPs were prepared by nanoprecipitation. Briefly, SQGem was dissolved in ethanol (8 mg ml^−1^) and it was mixed with the radiolabelled bioconjugate (^3^H-SQGem). The resulting solution was then added dropwise under magnetic stirring into 1 ml of MilliQ water (ethanol/water 0.5:1 v/v). Formation of the NPs occurred spontaneously without using any surfactant. After solvent evaporation under reduced pressure, an aqueous suspension of pure ^3^H-SQGem NPs was obtained (final SQGem concentration 4 mg ml^−1^). Final specific activity of ^3^H-SQGem NPs for *in vitro* and *in vivo* experiments was 40 μCi ml^−1^ and 50 μCi ml^−1^, respectively. For *in vivo* experiments, dextrose (5% w/v) was added into the final formulation for isotonicity. Mean particle size and polydispersity index were systematically determined after preparation by Dynamic Light Scattering using a Nano ZS (173° scattering angle) at 25 °C. (Malvern Instrument, UK). Measurements were carried out in triplicate.

### *In vitro* SQGem and Gem incubation with human blood

Fresh citrated blood of human volunteers was obtained from the Ambroise Paré hospital (Paris, France) and preserved in tubes containing tetrahydrouridine (THU; 8 μl ml^−1^). Blood samples were incubated with ^3^H-SQGem NPs (*n*=3; 155μM eq. Gem) or free ^3^H-Gem (*n*=3; 155 μM) for 5 min (or 15, 30, 60, 90 and 120 min) at 37 °C, followed by the centrifugation step (3,000*g*, 15 min, 15 °C) to separate plasma. The study was performed in accordance with the principles set out in the declaration of Helsinki and was approved by the Ethics Review Board of the institution (AP-HP). All healthy volunteers provided written informed consent to participate in the study (form of no-objection).

### Separation of lipoprotein fractions from human plasma

The separation of LP fractions from human plasma was performed employing a protocol currently used in clinical setting with minimal adaptation. In brief, 1 ml of previously separated human plasma was placed in ultracentrifuge tubes and then overlaid with 4.2 ml of 0.9% NaCl. Centrifugation was performed on a 70.1Ti rotor (Beckman Instruments, Inc.) and centrifuged (121,010*g*, 13 h 50 min, 4 °C) in an OptimaTM LE-80 K Ultracentrifuge (Beckman Instruments, Inc.). After the centrifugation, the upper fraction (∼4 ml) representing the VLDL solution was separated from the lower fractions (LDL+HDL+albumin) and the cellular debris pellet. Furthermore, VLDL and LDL particles were precipitated from their respective fractions using the cholesterol precipitant reagent (Biomerieux, France) according to the manufacturer's instructions. Finally, albumin was separated from the HDL fraction using PureProteome Albumin Magnetic Beads kit (Merck, Millipore), as indicated in the manufacturer's instructions. The albumin depletion was based on the interaction with the antibody specific for the human serum albumin, conjugated to the magnetic beads. After the removal of the magnetic beads, the supernatant represented the pure HDL fraction.

### *In vivo* SQGem and Gem administration in rats

All animals were housed in appropriate animal care facilities during the experimental period and were handled according to the principles of laboratory animal care and legislation in force in France. Experimental approval was obtained from the Ethical Committee C2EA—26 (Comité d'éthique en expérimentation animale de l'IRCIV, Authorization number 00245.02). Radiolabelled ^3^H-SQGem NPs or free ^3^H-Gem (5.9 mg kg^−1^ eq. Gem, 20 μCi per rat) were administered to healthy male Sprague Dawley rats (∼150 g, Janvier Labs, France) by intravenous (iv) injection through the tail vein (*n*=6). After 5 min, rats were anaesthetized using intraperitoneal (ip) injection of a mixture of ketamine (100 mg kg^−1^) and xylazine (10 mg kg^−1^) and blood was collected by cardiac puncture into Vacuette tubes EDTA K_3_ (Greiner Bio-One, France) containing tetrahydrouridine (THU; 8 μl ml^−1^), immediately centrifuged (1,700*g*, 15 min, 15 °C) and plasma was separated. ^3^H-SQAd NPs, ^3^H-Ad, ^3^H-LipoGem were intravenously injected following similar procedure (for details, see Supplementary Information Files).

### Separation of lipoprotein fractions from rat plasma

LP and LPDF were separated on the basis of their hydrated density with a modified single-step procedure according to the ultracentrifugation on sodium bromide (NaBr) gradient previously described by Cassidy *et al*.^57^. Briefly, a 1.006 g ml^−1^ density NaBr solution was placed at the bottom of the centrifugation tube and it was then carefully underlaid with increasing density NaBr solutions (1.063 and 1.21 g ml^−1^) and the rat plasma, whose density has been previously adjusted to 1.25 g ml^−1^ by addition of sodium bromide. Lipid-staining Sudan black (0.01% w/v) was added to the 1.25 g ml^−1^ solution (one tube) to visualize all the LP fractions. Centrifugation was performed on a SW 60 Ti swinging bucket rotor (Beckman Instruments, Inc.) at 164,326*g* for 14 h at 15 °C in an OptimaTM LE-80K Ultracentrifuge (Beckman Instruments, Inc.). On completion of the run, 20 fractions of 200 μl each were collected from the top to the bottom of the tube and the density of each fraction was measured.

### SQ bioconjugates and liposomes distribution

The distribution of radioactivity among the plasma fractions for both *in vitro* and *in vivo* experiments was determined using a β-scintillation counter (Beckman Coulter LS6500). To a previously measured volume of plasma and to each of the separated fractions, 10 ml of UltimaGold scintillation cocktail (Perkin Elmer) were added. In the case of precipitated pellets, samples were solubilized with Soluene-350 (Perkin Elmer) at 50 °C overnight before the addition of Hionic-Fluor scintillation cocktail (Perkin Elmer). Finally, all the samples were vigorously vortexed for 1 min and kept aside for 2 h before the counting.

### Cell lines

Human lung fibroblast cell line (MRC-5) and four human cancer cell lines (human breast carcinoma basal epithelial cell lines (MDA-MB-231 and MCF-7), ovarian adenocarcinoma cell line (SK-OV-3) and human adenocarcinoma alveolar basal epithelial cell line (A-549)) were obtained from ATCC (France) and maintained as recommended. Briefly, MDA-MB-231 cells were maintained in Leibovitz's L15 medium supplemented with 15% (v/v) FBS, glutamine (2 mM) and sodium hydrogen carbonate (20 mM). MCF-7 cells were grown in Dulbecco's Modified Eagle's Medium-high glucose (DMEM-F12) supplemented with 10% (v/v) heat inactivated FBS (56 °C, 30 min). SK-OV-3, A-549 and MRC-5 cells were cultured in McCoy's 5A, RPMI 1,640 medium and Eagle's Minimum Essential Medium, respectively, supplemented with 10% (v/v) FBS. All media were further supplemented with penicillin (50 U ml^−1^) and streptomycin (50 U ml^−1^) (Lonza, Levallois, France). Cells were maintained in a humid atmosphere at 37 °C with 5% CO_2_. The absence of mycoplasma was determined with the MycoAlertTM PLUS Mycoplasma Detection Kit (LONZA-LT07-705).

### LDL receptor expression

Cells were lysed in RIPA buffer (Sigma-Aldrich) supplemented with phosphatase and protease inhibitor cocktail (Sigma-Aldrich), vortexed and centrifuged (15 min, 11,000*g*, 4 °C). The concentration of extracted proteins was then measured using a colorimetric assay (Pierce BCA Protein Assay kit, Thermo Fisher Scientific). Equal amounts (30 μg) of proteins were incubated for 5 min at 99 °C with 4X Laemmli Sample Buffer supplemented with 5% ß-mercaptoethanol (Bio-Rad, France) and then separated on sodium dodecylsulfate-polyacrylamide gels (Mini-Protean-TGX 4–15%, Bio-Rad). Separated proteins were further transferred to polyvinylidene difluoride membrane using a liquid transfer system (100 V, 45 min). After blockage (5% dry milk suspension in 0.1% Tween 20 in TBS), the membrane was first incubated for 2 h at room temperature followed by an overnight incubation at 4 °C with the primary antibody solution. The membrane was then washed (3 × 15 min) in 0.1% Tween20-TBS buffer and incubated for 1 h at room temperature with secondary antibody solution. Antibodies used in the study: rabbit monoclonal anti-LDLR antibody (ab52818, Abcam) diluted 1/5,000, mouse anti-β-actin diluted 1/5,000 (AC-74, Sigma-Aldrich), goat anti-mouse secondary antibody conjugated to horseradish-peroxidase diluted 1/10,000 (sc-2005, Santa-Cruz Biotechnology) and goat anti-rabbit secondary antibody conjugated to horseradish-peroxidase diluted 1/10,000 (sc-2004, Santa-Cruz Biotechnology). Detection of chemiluminescence was performed using the Clarity Western ECL substrate (Bio-Rad Laboratories) and images were captured by the ChemiBIS system from DNR Bioimaging Systems (Thoiry, France).

### ^3^H-SQGem cellular uptake *in vitro*.

A total of 150,000 cells per well (MDA-MB-231) was seeded in 24-wells plates with culture medium supplemented with FBS (control) or LPDS (for LDLR modifications). 24 h post-seeding, an excessive amount of endogenous LDL (100 μg ml^−1^) was added in the wells for the competition studies. After 30 min, ^3^H-SQGem NPs, (previously incubated in culture medium containing FBS for 30 min at 37 °C to allow the interaction with serum LPs; *n*=3) or fluorescently labelled LDL (LDL-f; *n*=2) were added to each well (final Gem concentration 10 μM, 0.1 μCi ml^−1^; final LDL-f concentration 15 μg ml^−1^). After 90 min incubation media were removed, cells were washed 2-times with 1% BSA-PBS (1 ml) and 1-time with PBS (1 ml) and then treated with 0.2 ml of 0.25% trypsin for 5 min at 37 °C and 5% CO_2_. The action of trypsin was stopped by adding 0.8 ml of culture medium, the cellular suspension was centrifuged (200*g*, 5 min, 4 °C), supernatant discarded and the cells dispersed in 1 ml of PBS. Viable cells were counted according to the trypan blue exclusion method (0.4% trypan blue solution, Sigma Aldrich). The amount of internalized ^3^H-SQGem was determined using a β-scintillation counter (Beckman Coulter LS6500). Briefly, cellular suspensions were first solubilized with Soluene-350 (Perkin Elmer) at 50 °C overnight before the addition of Hionic-Fluor scintillation cocktail (Perkin Elmer). Finally, samples were vigorously vortexed for 1 min and kept aside for 2 h before the counting. For the cells incubated with LDL-f, the fluorescence was recorded using a C6 flow cytometer (Accuri Cytometers Ltd., UK). Excitation was carried out using a 488 nm argon laser and emission fluorescence was measured at 515 nm. The results were expressed as mean fluorescence intensity.

### ^3^H-SQGem cellular uptake *in vivo*

Athymic nude, four-week-old female mice were purchased from Envigo Laboratory (France). All animals were housed in appropriate animal care facilities during the experimental period and were handled according to the principles of laboratory animal care and legislation in force in France. Mice were fed with a cholesterol-rich (2%) diet (TD.01383, Harlan Teklad diets). After 4 weeks, cancer cells (MDA-MB-231 or MCF-7) were injected subcutaneously in the upper right flank of mice to develop a solid tumour model (5 × 10^6^ cells per mouse). Two weeks after tumour inoculation, mice were treated with ^3^H-SQGem NPs at dose of 5 mg kg^−1^ eq. Gem. After 6 h, mice (*n*=3 per group) were killed, tumours collected and the radioactivity was measured using liquid scintillation counting. In brief, to previously weighed tumours, 1 ml of Solvable (Perkin Elmer) was added for an overnight incubation at 50 °C. Then, after the addition of 100 μl of H_2_O_2_, vials were kept again overnight at 50 °C. Finally, Hionic-Fluor scintillation cocktail (Perkin Elmer) was added and the samples were vigorously vortexed for 1 min and kept aside for 2 h before the counting. No randomization method was used and no blinding was done.

### Data availability

Data supporting the findings of this study are available within the article (and its Supplementary Information Files) and from the corresponding author upon reasonable request.

## Additional information

**How to cite this article:** Sobot, D. *et al*. Conjugation of squalene to gemcitabine as unique approach exploiting endogenous lipoproteins for drug delivery. *Nat. Commun.* **8,** 15678 doi: 10.1038/ncomms15678 (2017).

**Publisher's note:** Springer Nature remains neutral with regard to jurisdictional claims in published maps and institutional affiliations.

## Supplementary Material

Supplementary Information Supplementary Figures, Supplementary Tables, Supplementary Methods and Supplementary References.

Peer Review File 2,569 kB
