# Supplementary material for: Conjugation of squalene to gemcitabine as unique approach exploiting endogenous lipoproteins for drug delivery
Source: Nat Commun. 2017 May 30;8:15678. doi: 10.1038/ncomms15678 (PMC5459998; doi:10.1038/ncomms15678)
Supplement: Supplementary Information — Supplementary Figures, Supplementary Tables, Supplementary Methods and Supplementary References. [file ncomms15678-s1.pdf]

Supplementary Methods.

Materials.

*Tris*norsqualenic acid was prepared as reported previously[1]. Gemcitabine base (98% minimum purity) and gemcitabine hydrochloride were purchased from Sequoia Research Products Ltd (UK) and [5'-^{3}H]-Gemcitabine hydrochloride was obtained from Moravek Biochemicals (USA). Squalene, ethyl chloroformate, triethylamine, adenosine, 1-hydroxybenzotriazole hydrate, 2-(1H-7-azabenzotriazol-1-yl)-1,1,3,3-tetramethyluronium hexafluorophosphate and tetrabutylammonium fluoride**,** dextrose, Trizma® hydrochloride, sodium chloride, sodium bromide and Sudan Black B were obtained from Sigma-Aldrich (France). Tetrahydrouridine (THU) was provided by MerckMillipore (San Diego,CA). Low-density lipoprotein from human plasma (LDL) and fluorescently labeled LDL (BODIPY® FL LDL) were obtained from ThermoFisher Scientific (France). 1,2-distearoyl-sn-glycero-3-phosphocholine (DSPC), cholesterol (Chol) and 1,2-distearoyl-sn-glycero-3-phosphoethanolamine-N-[amino(polyethylene glycol)-2000] (DSPE-PEG2000) were purchased from AvantiPolar (France). Deionized and filtered MilliQ® water was produced using a water purification system (Millipore, France).

Chemical synthesis

General details on the chemical characterization

IR spectra were obtained as solid or neat liquid on a Fourier Transform Bruker Vector 22 spectrometer. Only significant absorptions are listed. Optical rotations were measured on a Perkin-Elmer 241 Polarimeter at 589 nm. For SQGem and SQAd the ^{1}H and ^{13}C NMR spectra were recorded on Bruker Avance-300 spectrometers, at 300 MHz and 75 MHz, for ^{1}H and ^{13}C, respectively. Recognition of methyl, methylene, methine, and quaternary carbon nuclei in

^{13}C NMR spectra rests on the J-modulated spin-echo sequence. Mass spectra were recorded on a Bruker Esquire-LC. Analytical thin-layer chromatography was performed on Merck silica gel $60F_{254}$ glass pre-coated plates (0.25 mm layer). Column chromatography was performed on Merck silica gel 60 (230-400 mesh ASTM). Tetrahydrofuran (THF) was distilled from sodium/benzophenone ketyl. DMF and CH_2Cl_2 were distilled from calcium hydride, under a nitrogen atmosphere. Methanol and ethanol were dried over magnesium and distilled. All reactions involving air- or water-sensitive compounds were routinely conducted in glassware which was flame-dried under a positive pressure of nitrogen.

Synthesis of SQGem and ^{3}H-SQGem

SQGem and ^{3}H-SQGem were synthesized as previously described[2,3]. Practically, to a stirred solution of *tris*norsqualenic acid (0.5 g, 1.2 mmol) in anhydrous THF (3 mL), triethylamine (0.15 g, 1.4 mmol) was added under nitrogen. The mixture was cooled to –15 °C, and a solution of ethylchloroformate (0.135 g, 1.2 mmol) in anhydrous THF (3 mL) was added dropwise. The mixture was stirred at –15 °C for 15 min and anhydrous DMF (5 mL) was added to the reaction mixture at the same temperature followed by solid gemcitabine base (0.39 g, 1.5 mmol) at once. The reaction mixture was stirred for 72 h at 22 °C and then concentrated *in vacuo*. Aqueous sodium hydrogen carbonate was added and the mixture was extracted with ethyl acetate (3 x 50 mL). The combined extracts were washed with water, dried over $MgSO_4$, and concentrated under reduced pressure. The crude product was purified by chromatography on silica gel eluting with 1 to 5% methanol in dichloromethane to give pure 4-*N*-trisnorsqualenoyl-gemcitabine as an amorphous white solid (0.46 g, 57%). $[\alpha]_D$ = 3.1 (c = 0.95, CH_2Cl_2); IR (neat, cm^{-1}) 3500-3150, 2950, 2921, 2856, 1709, 1656, 1635, 1557, 1490, 1435, 1384, 1319, 1275, 1197, 1130, 1071; ^{1}H NMR (300 MHz, $CDCl_3$) δ 9.40 (broad s, 1 H, NHCO), 8.17 (d, 1 H, *J* = 7.2 Hz, H6), 7.48 (d, 1 H, *J* = 7.2 Hz, H5), 6.17 (t, 1 H, *J* =

7.0 Hz, H1'), 5.50-5.20 (br s, 1H, OH), 5.22-5.15 (m, 5 H, *H*C=C(CH_3)), 4.55-4.35 (m, 1 H, H3'), 4.10-3.55 (m, 2 H, H4' and H5'), 3.90 (d, *J* = 11.2 Hz, H-5'), 2.61-2.50 (m, 2 H), 2.35-2.28 (m, 2 H), 2.12-1.90 (m, 16 H, CH_2), 1.67 (s, 3H, C=C(CH_3)$_2$), 1.69-1.55 (m, 15H, C=C(CH_3)); ^{13}C NMR (75 MHz, $CDCl_3$) δ 173.7 (*C*ONH), 163.0 (HN*C*=N), 155.8 (N*C*ON), 145.4 (CH, C6), 135.1 (C), 134.9 (2 C), 132.7 (C), 131.2 (C), 125.7 (CH), 124.4 (2 CH), 124.2 (2 CH), 122.4 (CF_2), 97.7 (CH, C5), 81.7 (2 CH), 69.2 (CH), 59.9 (CH_2), 39.7 (2 CH_2), 39.5 (CH_2), 36.5 (CH_2), 34.3 (CH_2), 28.3 (2 CH_2), 26.8 (CH_2), 26.7 (CH_2), 26.6 (CH_2), 25.6 (CH_3), 17.6 (CH_3), 16.0 (3 CH_3), 15.8 (CH_3); MS (CI, isobutane): *m/z* (%): 646 (100%); Anal. Calcd for $C_{36}H_{53}N_3O_5$: C, 66.95, H, 8.27, N, 6.51. Found: C, 66.76, H, 8.40, N, 6.39.

^{3}H-SQGem (2.89 mCi mmol^{-1}) was synthesized in a similar manner, but using ^{3}H-gemcitabine (1.6 mCi mmol^{-1}). 3.5 mCi of [5'-^{3}H]-gemcitabine was mixed with cold gemcitabine hydrochloride (10 mg) to obtain 10 mg of tritiated gemcitabine hydrochloride (3.5 mCi, 0.033 mmol). The coupling with trisnorsqualenic acid was then realized according to the experimental procedure described above in the presence of 1.1 equiv. of triethylamine to provide pure tritiated 4-(*N*)-trisnorsqualenoylgemcitabine ([5'-^{3}H]SQGem) (0.017 g, 84%, 84.2 mCi mmol^{-1}). The specific activity was adjusted to 2.89 mCi mmol^{-1} by mixing this material together with cold SQGem (343 mg) to finally obtain 360 mg of [5'-^{3}H]SQGem. The product was stored at –20 °C in 60 mL of a solution of acetone/ethanol (1:1).

Synthesis of SQAd and ^{3}H-SQAd

Squalene-Adenosine (SQAd) and radiolabeled SQAd (^{3}H-SQAd) were synthesized as previously reported.[4] Briefly, to a solution of tris-O-silylated N^6-squalenylacetic-acid adenosine (2.20 g, 2.08 mmol) in dry THF (25 mL) was added n-Bu_4NF (1M solution in THF, 7.53 mL, 7.53 mmol) and the mixture was stirred for 3 h at room temperature. The reaction was then quenched with brine and the mixture was extracted with ethyl acetate (3 × 20 mL).

The combined extracts were dried on $MgSO_4$, and concentrated under vacuum. The crude product was purified by chromatography on silica gel eluting with 5% methanol in dichloromethane to give pure N^6-squalenylacetic-acid adenosine as a colourless oil (0.820 g, 55 %); $[\alpha]_D = -35$ (c = 0.4, $CHCl_3$); IR (film, cm^{-1}): 3500-3000, 2965, 2915, 2854 1681, 1614, 1584, 1536, 1462, 1376, 1353, 1318, 1304, 1224, 1191, 1150, 1104, 1079, 1041; ^1H NMR (300 MHz, DMSO) δ: 10.69 (broad s, 1 H, NHCO), 8.70 (s, 1 H, H8), 8.64 (s, 1 H, H2), 6.01 (d, 1 H, J = 5.4 Hz, H1'), 5.15 (t, 1 H, J = 6.3 Hz, $HC=C(CH_3)$), 5.05-5.14 (m, 5 H, $HC=C(CH_3)$), 4.61 (t, 1 H, J = 4.9 Hz, H2'), 4.18 (m, 1 H, H3'), 3.98 (m, 1 H, H4'), 3.69 (dd, 1 H, J = 11.9 Hz, J = 3.7 Hz, H5'), 3.57 (dd, J = 11.9 Hz, J = 3.3 Hz, 1 H, H5'), 2.66 (t, 2 H, J = 7.8 Hz, $O_2CCH_2CH_2$), 2.27 (t, 2 H, J = 7.8 Hz, $O_2CCH_2CH_2$), 2.05-1.91 (m, 20 H, $=C(CH_3)CH_2CH_2$), 1.62 (s, 3 H, $C=C(CH_3)$), 1.60 (s, 3 H, $C=C(CH_3)$), 1.54 (s, 15 H, $C=C(CH_3)$); ^{13}C NMR (75 MHz, $CDCl_3$) some carbons of the purine ring were not detected δ: 173.0 (CONH), 150.0 (C4), 149.5 (C6), 135.1 (C, $HC=C(CH_3)$), 135.0 (C, $HC=C(CH_3)$), 134.9 (C, $HC=C(CH_3)$), 134.8 (C, $HC=C(CH_3)$), 133.1 (C, $HC=C(CH_3)$), 131.2 (C, $HC=C(CH_3)$), 125.7 (CH, $HC=C(CH_3)$), 124.45 (CH, $HC=C(CH_3)$), 124.38 (CH, $HC=C(CH_3)$), 124.28 (CH, $HC=C(CH_3)$), 24.26 (2CH, $HC=C(CH_3)$), 122.8 (C5), 91.0 (CH, C1'), 87.4 (CH, C2'), 74.0 (CH, C4'), 72.3 (CH, C3'), 62.9 (CH_2, C5'), 39.75 (CH_2), 39.73 (CH_2), 39.71 (CH_2), 39.6 (CH_2), 36.7 (CH_2), 34.4 (CH_2), 31.9 (CH_2), 29.7 (CH_2), 28.3 (CH_2), 26.9 (CH_2), 26.7 ($2CH_2$), 26.6 (CH_2), 25.7 (CH_3), 17.6 (CH_3), 16.06 (CH_3), 16.04 (CH_3), 16.02 (CH_3), 16.01 (CH_3), 15.98 (CH_3). MS (+APCI) m/z (%) 718.7 (100) $[M+H]^+$, 586.5 (12) $[M-C_5H_8O_4+H]^+$, 483.5 (60), 268.3 (22); HRMS (+ESI) m/z calcd for $C_{42}H_{63}N_5O_5Na$ $[M+Na]^+$ 740.4753, found 740.4727; Anal. Calcd for $C_{42}H_{63}N_5O_5$. H_2O: C, 68.54, H, 8.90, N, 9.52. Found: C, 68.35, H, 8.84, N, 9.36.

For radioactive synthesis, ^3H-SQAd was obtained by H/T exchange with HTO. A suspension containing HTO in dioxane-d_8 was introduced into a NMR tube containing dry SQAd and

heated at 105 °C in the dark for 16 h. The slightly yellow solution obtained was diluted with MeOH and the solvent was evaporated. The crude product was purified by preparative HPLC to give 12 mCi of pure SQ-[^{3}H]-Ad with a specific activity of 1.95 Ci mmol^{-1}. The product has been conserved as a solution of 1,4-dioxane (C = 1.92 mCi mL^{-1}) at -20 °C.

Synthesis of SQCy5.5 and SQCy7.5

Non-symmetrical cyanine, bearing an alcohol group, was synthesized similarly to a described procedure[5]. Then, this alcohol group was acylated with squalenic acid to obtain the target compounds Squalene-C6-Cy5.5 (SQCy5.5) and Squalene-C6-Cy7.5 (SQ Cy7.5).

Briefly, 3-(6-Hydroxyhexyl)-1,1,2-trimethyl-1H-benzo[e]indol-3-ium chloride (1.2 eq., 526 mg, 1.35 mmol) and 3-Ethyl-1,1-dimethyl-2-[(1*E*,3*E*)-4-(*N*-phenylacetamido)buta-1,3-dien-1-yl]-1H-benzo[e]indol-3-ium chloride (1 eq., 500 mg, 1.12 mmol) were mixed in dry pyridine (5 mL) and heated at 50 °C for 1 h. After completion of the reaction, the mixture was concentrated at reduced pressure, and the residue dissolved in dichloromethane (DCM). This solution was washed with 0.1 M HCl, brine and dried over Na_2SO_4. After filtering off the Na_2SO_4 and concentrating the solution, the crude product was purified by gradient column chromatography on silica gel (DCM/MeOH 99:1 – 90:10) to give Cy5.5-C6-OH (390 mg, 0.63 mmol, 56%). ^{1}H NMR (400 MHz, MeOH-d_4) δ 8.34 - 8.43 (2 H, m), 8.25 (2 H, br d, J = 8.3 Hz), 7.95 - 8.04 (4 H, m), 7.61 - 7.66 (4 H, m), 7.43 - 7.51 (2 H, m), 6.72 (1 H, br t, J = 12.1 Hz), 6.32 - 6.40 (2 H, m), 4.28 (4 H, m), 3.53 - 3.61 (2 H, m), 2.02 (12 H, br s), 1.84 - 1.94 (2 H, m), 1.45 - 1.58 (9 H, m) ppm. ^{13}C NMR (126 MHz, MeOH-d_4) δ 174.47 (s), 174.15 (s), 153.06 (s), 152.92 (s), 139.61 (s), 139.16 (s), 133.86 (s), 133.71 (s), 132.07 (s), 132.02 (s), 130.39 (s), 130.32 (s), 129.73 (s), 128.13 (s), 128.08 (s), 127.35 (s), 127.33 (s), 125.15 (s), 124.68 (s), 121.97 (s), 110.72 (s), 110.48 (s), 102.55 (s), 102.28 (s), 61.33 (s), 51.08 (s), 51.04

(s), 43.67 (s), 38.84 (s), 32.03 (s), 27.37 (s), 26.29 (s), 26.19 (s), 25.33 (s), 11.53 (s) ppm. HRMS (*m*/*z*): [M]+ calcd. for $C_{41}H_{47}N_2O^+$, 583.36884; found 583.36852.

For the synthesis of Cy7.5-C6-OH a mixture of 3-(6-Hydroxyhexyl)-1,1,2-trimethyl-1H-benzo[e]indol-3-ium chloride (1.2 eq., 497 mg, 1.27 mmol) and 3-Ethyl-1,1-dimethyl-2-[(1*E*,3*E*,5*E*)-6-(*N*-Phenylacetamido)hexa-1,3,5-trien-1-yl]-1H-benzo[e]indol-3-ium chloride (1 eq., 500 mg, 1.06 mmol) in dry pyridine (5 mL) was heated at 50 °C for 1 h. Then, the mixture was concentrated under reduced pressure, and the residue dissolved in DCM. The solution was washed with 0.1 M HCl, brine and dried over Na_2SO_4. After filtering off the Na_2SO_4 and concentrating the solution, the crude product was purified by gradient chromatography on silica gel (DCM/MeOH 99/1 - 90/10) to give Cy7.5-C6-OH (337 mg, 0.522 mmol, 49 %). ^{1}H NMR (400 MHz, MeOH-d_4) δ 8.24 (2 H, br d, *J* = 8.5 Hz), 7.97 - 8.10 (6 H, m), 7.56 - 7.73 (5 H, m), 7.48 (2 H, br t, *J* = 7.4 Hz), 6.58 - 6.67 (2 H, t, *J* = 12.1 Hz), 6.36 (2 H, m), 4.28 (2 H, m), 4.23 (2 H, br t, *J* = 7.3 Hz), 3.58 (2 H, br t, *J* = 6.0 Hz), 2.00 (12 H, s), 1.84 - 1.94 (2H, m), 1.41 - 1.61 (9 H, m) ppm.^{13}C NMR (126 MHz, MeOH-d_4) δ 172.96 (s), 172.74 (s), 150.78 (s), 150.59 (s), 139.78 (s), 139.32 (s), 133.59 (s), 133.41 (s), 131.95 (s), 131.90 (s), 130.38 (s), 130.30 (s), 129.71 (s), 128.15 (s), 128.11 (s), 127.31 (s), 125.62 (s), 124.55 (s), 121.94 (s), 110.67 (s), 110.44 (s), 103.00 (s), 102.80 (s), 61.35 (s), 50.82 (s), 50.76 (s), 43.69 (s), 38.86 (s), 32.04 (s), 27.39 (s), 26.30 (s), 26.19 (s), 25.35 (s), 11.59 (s) ppm. HRMS (*m*/*z*): $[M]^+$ calcd. for $C_{43}H_{49}N_2O^+$, 609.38449; found 609.38545.

Squalene-C6-Cy5.5, (SQCy5.5) (4*E*,8E,16*E*)-4,8,13,17,21-Pentamethyldocosa-4,8,12,16,20-pentaenoic acid (1.1 eq., 28.5 mg, 0.071. mmol), Cy5.5-C6-OH (1 eq., 40 mg, 0.064 mmol), 1-ethyl-3-(3-dimethylaminopropyl)carbodiimide hydrochloride (1.5 eq., 18.6 mg, 0.097 mmol) and DMAP (0.2 eq., 1.58 mg, 0.013 mmol) were mixed in DCM (0.8 mL) and the resulting mixture was stirred overnight. After completion of the reaction, the mixture was

purified by gradient chromatography DCM/MeOH 99:1 – 90:10 using a Puriflash 430 to afford SQCy5.5 (46 mg, 0.046 mmol, 71%). ^{1}H NMR (500 MHz, MeOH-d_4 + 5% $CDCl_3$) δ 8.27 - 8.34 (2 H, m), 8.21 (2 H, d, *J* = 8.5 Hz), 7.95 - 8.01 (4 H, m), 7.64 (2 H, m), 7.53 (2 H, m), 7.48 (2 H, t, *J* = 7.4 Hz), 6.67 (1 H, t, *J* = 12.1 Hz), 6.30 (2 H, t, *J* = 13.9 Hz), 5.03 - 5.10 (5 H, m), 4.26 (2 H, q), 4.21 (2 H, br t, *J* = 7.3 Hz), 4.06 (2 H, t, *J* = 6.6 Hz), 2.34 - 2.39 (2 H, m), 2.21 - 2.26 (2 H, m), 2.04 (18 H, m), 1.89 - 1.96 (12 H, m), 1.61 - 1.68 (5 H, m), 1.51 - 1.57 (19 H, m), 1.45 - 1.49 (3 H, m) ppm. ^{13}C NMR (126 MHz, MeOH-d_4+ 5% $CDCl_3$) δ 174.35 (s), 174.18 (s), 173.97 (s), 152.92 (s), 152.70 (s), 139.49 (s), 139.04 (s), 134.66 (s), 134.50 (s), 134.43 (s), 133.95 (s), 133.73 (s), 133.00 (s), 132.10 (s), 132.02 (s), 130.73 (s), 130.59 (s), 130.52 (s), 129.87 (s), 128.15 (s), 128.14 (s), 127.53 (s), 127.51 (s), 125.23 (s), 124.97 (s), 124.88 (s), 124.87 (s), 124.38 (s), 124.19 (s), 124.16 (s), 124.09 (s), 122.00 (s), 110.58 (s), 110.42 (s), 102.51 (s), 102.39 (s), 64.03 (s), 51.17 (s), 51.09 (s), 43.81 (s), 39.54 (s), 39.52 (s), 39.38 (s), 39.06 (s), 34.51 (s), 32.95 (s), 28.30 (s), 27.92 (s), 27.43 (s), 26.77 (s), 26.64 (s), 26.52 (s), 26.29 (s), 26.26 (s), 26.22 (s), 25.56 (s), 25.04 (s), 24.88 (s), 16.80 (s), 15.21 (s), 15.19 (s), 15.16 (s), 15.10 (s), 11.91 (s) ppm. HRMS (*m*/*z*): $[M]^+$ calcd. for $C_{68}H_{89}N_2O_2^+$, 965.69241; found 965.69410.

Squalene-C6-Cy7.5, (SQCy7.5) A mixture of (4*E*,8*E*,16*E*)-4,8,13,17,21-pentamethyldocosa-4,8,12,16,20-pentaenoic acid (1.1 eq., 27.3 mg, 0.068 mmol), Cy7.5-C6-OH (1 eq., 40 mg, 0.062 mmol), 1-ethyl-3-(3-dimethylaminopropyl)carbodiimide hydrochloride (1.5 eq., 17.8 mg, 0.093 mmol) and DMAP (0.2 eq, 1.51 mg, 0.012 mmol) in DCM (0.8 mL) was stirred overnight at room temperature. After completion of the reaction, the mixture was purified by gradient chromatography DCM/MeOH 99:1 – 90:10 using a Puriflash 430 to give SQCy7.5 (49 mg, 0.047 mmol, 77 %). ^{1}H NMR (400 MHz, CH_3CN-d_3, MeOH-d_4 50/50) δ 8.25 (2 H, br d, *J* = 7.8 Hz), 7.97 - 8.06 (6 H, m), 7.66 (3 H, br s), 7.46 - 7.60 (4 H, m), 6.59 (2 H, br t),

6.31 (2 H, br t), 5.12 (5 H, br s), 4.19 - 4.27 (2 H, m), 4.15 (2 H, br s), 4.07 (2 H, br s), 2.35 - 2.42 (2 H, m), 2.26 (2 H, m), 1.91 - 2.12 (28 H, m), 1.86 (2 H, br s), 1.66 (5 H, br s), 1.47 – 1.62 (19 H, m), 1.44 (3 H, m) ppm.^{13}C NMR (126 MHz, CH_3CN-d_3, MeOH-d_4 50/50) δ 174.55 (s), 173.92 (s), 173.79 (s), 151.63 (s), 151.15 (s), 140.98 (s), 140.52 (s), 135.80 (s), 135.67 (s), 135.63 (s), 134.77 (s), 134.43 (s), 134.34 (s), 132.87 (s), 132.76 (s), 131.95 (s), 131.43 (s), 131.33 (s), 130.86 (s), 130.84 (s), 129.08 (s), 128.53 (s), 128.52 (s), 126.35 (s), 125.81 (s), 125.78 (s), 125.73 (s), 125.36 (s), 125.36 (s), 125.23 (s), 125.18 (s), 125.12 (s), 123.19 (s), 123.18 (s), 125.23 (s), 125.18 (s), 125.12 (s), 111.96 (s), 111.83 (s), 103.99 (s), 64.93 (s), 51.92 (s), 51.77 (s), 44.85 (s), 40.46 (s), 40.43 (s), 40.31 (s), 40.14 (s), 35.44 (s), 33.84 (s), 29.23 (s), 28.86 (s), 28.25 (s), 27.47 (s), 27.44 (s), 27.30 (s), 27.23 (s), 27.20 (s), 27.04 (s), 26.44 (s), 25.73 (s), 17.65 (s), 16.05 (s), 16.03 (s), 16.00 (s), 15.95 (s), 12.79 (s) ppm. HRMS (m/z): [M]+ calcd. for $C_{70}H_{91}N_2O_2+$, 991.70806; found 991.71013.

Formulation of nanoparticles and liposomes

^{3}H-AdSQ nanoparticles

^{3}H-SQAd nanoparticles were prepared by nanoprecipitation. Briefly, SQAd was dissolved in ethanol (3 mg mL^{-1}) and mixed with the radiolabeled bioconjugate (^{3}H-SQAd). The resulting solution was then added dropwise under magnetic stirring to a 5% (w/v) aqueous dextrose solution (ethanol/dextrose solution 1:3 v/v). Formation of the NPs occurred spontaneously without using any surfactant. After solvent evaporation under reduced pressure, an aqueous suspension of pure ^{3}H-SQAd NPs was obtained (final SQAd concentration 1 mg mL^{-1}). Final volume activity of ^{3}H-SQAd NPs was 7 µCi mL^{-1}. Mean particle sizes and polydispersity indexes were systematically determined after preparation by Dynamic Light Scattering (DLS) using a Nano ZS (173° scattering angle) at 25 °C. (Malvern Instrument, UK). NPs surface

charge was investigated using the same apparatus by zeta potential measurements at 25 °C after dilution with 1 mM NaCl solution applying the Smoluchowski equation. Measurements were carried out in duplicate.

SQCy5.5 nanoparticles

SQCy5.5 nanoparticles were prepared by nanoprecipitation. Briefly, SQCy5.5 was dissolved in acetone (4 mg mL^{-1}) and then added dropwise under magnetic stirring into 1 mL of MilliQ® water (ethanol/water 0.5:1 v/v). Formation of the NPs occurred spontaneously without using any surfactant. After solvent evaporation under reduced pressure, an aqueous dispersion of pure SQCy5.5 NPs was obtained (final SQCy5.5 concentration 2 mg mL^{-1}). Mean particle sizes and polydispersity indexes were systematically determined after preparation by Dynamic Light Scattering (DLS) using a Nano ZS (173° scattering angle) at 25 °C. (Malvern Instrument, UK). NPs surface charge was investigated using the same apparatus by zeta potential measurements at 25 °C after dilution with 1 mM NaCl solution, applying the Smoluchowski equation. Measurements were carried out in duplicate.

FRET nanoparticles

FRET NPs were prepared by nanoprecipitation. Briefly, 2 mg of SQGem mixed with SQCy5.5 (0.6% w/w) and SQCy7.5 (0.6 % w/w) were dissolved in ethanol. The resulting solution was added dropwise under magnetical stirring into MilliQ water (ethanol/water 0.5:1 v/v). Ethanol was then evaporated under reduced pressure and a suspension of FRET NPs (final concentration of SQGem 2 mg mL^{-1}) was obtained. Mean diameter and polydispersity index were determined by dynamic light scattering at 25 °C (Zetasizer Nano ZS, Malvern Instruments, UK, 173° scattering angle). NPs surface charge was investigated using the same

apparatus by zeta potential measurements at 25 °C after dilution with 1 mM NaCl solution applying the Smoluchowski equation. All measurements were repeated at least three times.

Absorbance spectra of these NPs were measured with a UV-visible spectrophotometer (Lambda 25, Perkin Elmer, USA). Fluorescence emission spectra were measured with a spectrofluorometer (SPEX1681, Horiba, Japan). Measurements were performed after opportune dilution of NPs, which assured an absorbance ≤ 0.1 at the excitation wavelengths and a total dye concentration < 1 µM.

^{3}H-Gemcitabine-loaded liposomes (^{3}H-LipoGem)

^{3}H-Gemcitabine-loaded liposomes were prepared as previously reported with slight modifications.[7] Briefly, lipids (DSPC:Chol:DSPE-PEG2000, 60:30:10 mol%, 80 mg) were dissolved in $CHCl_3$/MeOH 3:1 (5 mL) and introduced in a round-bottom flask (100 mL). The lipid film was obtained by stirring at 60 °C under reduced pressure (slowly reduced to 100 mbars for 15 min) using a rotary evaporator and then drying under high vacuum (0.05 mbar) for 15 h. The film was then rehydrated at 60 °C with ammonium sulfate (250 mM, pH 5.4, 2 mL) under stirring in the presence of glass beads for 15 min. The lipid suspension was then extruded using a stainless steel extrusion device (LIPEX extruder, Whitley, Canada), at 60 °C, through polycarbonate membrane filters (Millipore) (5 x 400 nm, 5 x 200 nm, 8 x 100 nm) before ultracentrifugation (147000 *g*, 1.5 h). The pellet was resuspended in a solution of Gem (free base form, 20 mM) in PBS (1 mL, final lipid concentration 91.9 mM). The suspension was then introduced into a tube containing the ^{3}H-Gem solution (50 µCi,1 mCi mL^{-1}) previously dried by N_2 flow for 30 min. The mixture was shaken (800 rpm) at 60 °C for 20 h (Thermo-Shaker, TS-100, Labgene Scientific Instrument). The ^{3}H-Gemcitabine loaded liposomes were purified by size exclusion chromatography (PD

MidiTrap G-25) for removing the unentrapped drug and recovered in PBS (1 mL) (loading efficiency 12 ± 2%). Final volume activity of ^{3}H-LipoGem was 6 µCi mL^{-1}.

Analysis of Human plasma fractions

Plasma lipid and protein profile determination

Lipid parameters (total cholesterol, triglycerides, HDL-C, LDL-C, apoA1 and apoB) and albuminemia for each blood sample were determined at the Georges Pompidou European Hospital (France) using standard laboratory procedures with DxC800® and DxI800® (Beckman-Coulter, Villepinte, France). All patients were normolipidemic and had normal albuminemia. Molar concentrations of LDL and HDL were calculated using the Equations 1 and 2, respectively[8]. The equations took into account molecular weight of LDL and HDL (2.3x10^{6} and 1.8x10^{5} Da, respectively) as well as the percentage of apoprotein content in corresponding LP particle (21% for apoB and 50% for apoA).

$$[\mathrm{LDL}\ (\mathrm{mol\ L^{-1}})] = \frac{[\mathrm{apoB}\ (\mathrm{g\ L^{-1}})]}{0.21\ \times\ 2.3 \times 10^{6}} \quad (1)$$

$$[\mathrm{HDL}\ (\mathrm{mol\ L^{-1}})] = \frac{[\mathrm{apoA}\ (\mathrm{g\ L^{-1}})]}{0.5\ \times\ 1.8\ \times\ 10^{5}} \quad (2)$$

Treatment and analysis of rat plasma fractions

Dialysis of separated fractions from rat plasma

Reduced volume dialysis was performed to remove the NaBr from the collected fractions using Slide-A-Lyzer MINI dialysis devices (3.5K MWCO, Thermo Fisher). Fractions were dialyzed against Tris-buffered saline (50 mM TrisHCl, 150 mM NaCl, pH 7.4) during 2 h

with the addition of the fresh buffer every 30 min. Volume of each fraction was measured before and after the dialysis and the recovery factors were calculated.

Electrophoresis

The purity of each of the separated LP fractions from rat plasma was determined using the agarose gels electrophoresis kit for LP separation according to manufacturer instructions (HYDRAGEL LIPO + Lp(a) K20, Sebia). Briefly, 10 µL of each fraction and total plasma were placed on the gel and were allowed to migrate for 90 minutes at constant voltage (50 V). After the migration, the gel was dried for 45 min at 80 °C and stained using Sudan Black. Finally, after the steps of discoloration and washing, the migrated LP fractions were visualized and attributed to a specific LP class. Total plasma was used as control.

Albumin and cholesterol dosage

Albumin and cholesterol content in all the separated fractions obtained from rat plasma was determined using albumin rat ELISA kit (ab108790, Abcam) and cholesterol assay kit (ab65390, Abcam) according to manufacturer's instructions.

In silico modeling

***In silico* modeling**

A slice of the LDL particle which contained 10% of its full volume has been simulated (Supplementary Figures 18, 19). This made the system computationally tractable on atomistic level, while keeping realistic lipid composition and all major physico-chemical ingredients of the LDL lipid droplet. ApoB protein was not included into simulations since interactions with the lipid core of the particle represented the key event in the interaction of SQGem

nanoparticles with LDL. The lipid composition of the system used for LDL simulation is summarized in Supplementary Table 2. This composition has been chosen according to typical human LDL particle and to the first coarse-grained modeling of LDL[9]. For molecular dynamics simulations of LDL-SQGem and LDL-Gem interaction, the system was designed as follows. The hydrophobic core containing cholesterol, cholesterol oleate and glyceryl trioleate was constructed as a random mixture of these compounds and simulated for 50 ns until it reaches its equilibrium density. After that, two identical surface layers containing POPC and Lyso PC molecules were added to the sides of this core. The system was then solvated at both sides of the surface layers and subject to long equilibration runs of 500 ns. A snapshot of the equilibrated system is shown in Supplementary Figure 19. Note that Slipids force field[10] was used for all lipids. Topologies for lyso PC, cholesterol oleate and glyceryl trioleate were constructed manually by combining existing Slipids topologies. Initial topologies of Gem and SQGem were generated by Acpype topology generator[11]. The structure of gemcitabine was optimized in Gaussian09[12] at the B3LYP/6-31++G(d) level of theory. Partial charges of the substituted sugar ring were calculated in order to map the electrostatic potential (ESP charges) and added to the initial topologies of Gem ans SQGem. The charges of other Gem atoms were taken from the Amber 99 nucleic acids to keep the maximal consistency with the rest of the force field. The atom types of squalene tail of SQGem were adjusted to match the Slipids force field.

All molecular dynamics simulations were performed with the Gromacs 5.0.4 package[13]. Preparation and analysis of the systems were performed using Pteros molecular modeling library[14,15]. TIP3P water model was used[16]. All simulations were performed at constant temperature of 320 K and constant pressure of 1 bar (NPT conditions) maintained by v-rescale thermostat[17] and Berendsen barostat[18], respectively. Profiles of mean force (PMFs) of transferring Gem and SQGem molecules from water to the core of LDL particle were

computed by umbrella sampling simulations. The center of masses of Gem or SQGem molecule was restrained by harmonic potential characterized by a 2,000 kJ mol^{-1} nm^{-2} force constant at different distances from the center of the slice. This resulted in 90 umbrella sampling windows each separated by 0.1 nm along Z axis. Each window was sampled during 200 ns. PMFs were obtained with the weighted histogram technique[19] as implemented in Gromacs package using the last 100 ns of each window for stastistical analysis.

SQGem NPs and Lipoproteins interaction

ITC analysis

Isothermal titration calorimetry (ITC) experiments (n=2) were performed at 37 °C in PBS with an ITC200 calorimeter (Microcal® Malvern). The lipoprotein concentration in the microcalorimeter cell and the SQGem concentration in the syringe were set at 10 µM and 100 µM, respectively. A first injection of 0.4 µL was followed by 20 injections of 2 µL at intervals of 180 s. The data were analyzed according to the one binding-site model using the MicroCal Origin software provided by the manufacturer.

Transmission electron microscopy (TEM)

A physical mixture of SQGem NPs (final concentration 500 µg mL^{-1}) and LDL (final concentration 50 µg mL^{-1}) was incubated for 5 minutes at 37 °C immediately before grid preparation. Then, five microliters of the mixture were deposited on Formvar/carbon 400 Mesh Copper grid. After 5 min at room temperature, the remaining drop was blotted with a filter paper. A drop of phosphotungstic acid 2% filtered on 0.22 µm was added for 30 sec for negative staining and the excess of volume was removed with a blotting filter paper. The grids were then observed with a JEOL 1400 120 kV electron microscope operating at 80 kV at a

nominal magnification of 5000−40,000. Digital images were directly recorded on a CCD postcolumn high-resolution (11 MegaPixel) high-speed camera (SC1000 Orius, Gatan Inc.) using Digital Micrograph image acquisition and processing software (Gatan Inc.).

Images of SQGem NPs (1 mg mL^{-1}) and LDL dispersion (25 µg mL^{-1}) were acquired according to the same protocol unless the incubation at 37 °C.

In vivo studies

All animals were housed in appropriate animal care facilities during the experimental period and were handled according to the principles of laboratory animal care and legislation in force in France. *(*Authorization number 00245.02).

^{3}H-SQAd NPs and ^{3}H-Ad administration in rats

Radiolabeled ^{3}H-SQAd NPs (1.7 mg kg^{-1} eq. Ad, 5.25 µCi per rat, n=4) or free ^{3}H-Ad (2.2 mg kg^{-1} eq. Ad, 20 µCi per rat, n=2) were administered to healthy male Sprague Dawley rats (~150 g, Janvier Labs, France) by intravenous injection through the tail vein. After 5 minutes, rats were anesthetized using intraperitoneal (ip) injection of a mixture of ketamine (100 mg kg^{-1}) and xylazine (10 mg kg^{-1}) and blood was collected by cardiac puncture into Vacuette® tubes EDTA K_3 (Greiner Bio-One, France), immediately centrifuged (1700 *g*, 15 min, 15 °C) and plasma was separated.

SQCy5.5 administration in rats

SQCy5.5 NPs (4.2 mg kg^{-1}, n=4) were administered to healthy male Sprague Dawley rats (~150 g, Janvier Labs, France) by intravenous injection through the tail vein. After 5 minutes, rats were anesthetized using intraperitoneal (ip) injection of a mixture of ketamine (100 mg kg^{-1}) and xylazine (10 mg kg^{-1}) and blood was collected by cardiac puncture into Vacuette®

tubes EDTA K_3 (Greiner Bio-One, France) immediately centrifuged (1700 *g*, 15 min, 15 °C) and plasma was separated. The distribution of SQCy5.5 among the plasma fractions was determined using a spectrofluorometer (SPEX1681, Horiba, Japan).

^{3}H-Gemcitabine-loaded liposomes administration in rats

^{3}H-Gem loaded liposomes (2.8 mg kg^{-1} eq. Gem, 4.5 µCi per rat, n=4) were administered to healthy male Sprague Dawley rats (~150 g, Janvier Labs, France) by intravenous injection through the tail vein. After 5 minutes, rats were anesthetized using intraperitoneal (ip) injection of a mixture of ketamine (100 mg kg^{-1}) and xylazine (10 mg kg^{-1}) and blood was collected by cardiac puncture into Vacuette® tubes EDTA K_3 (Greiner Bio-One, France), immediately centrifuged (1700 *g*, 15 min, 15 °C) and plasma was separated.

^{3}H-LipoGem control study

^{3}H-LipoGem were incubated with 1 mL of 1.25 g mL^{-1} NaBr solution (final volume activity 0.27 µCi mL^{-1}) for 5 minutes at 37 °C (n=2). Then, a density gradient ultracentrifugation was performed as described for lipoprotein separation unless replacing the rat plasma by this ^{3}H-LipoGem-containing NaBr solution.

FRET NPs stability investigation

Animals

Male Sprague Dawley rats (~150 g) were bough form Janvier Labs (France). Animals were housed in an appropriate animal care facility during the experimental period. Experiments were carried out according to the principles of laboratory animal care and legislation in force in France. Rat blood was obtained by cardiac puncture under deep terminal anesthesia with pentobarbital, and collected in Vacuette® tubes EDTA K_3 (Greiner Bio-One, France)

In vitro stability study

FRET NPs were diluted (1:6) with MilliQ® water, rat blood or ethanol and incubated at 37 °C under stirring over a period of 24 h. Immediately after mixing (t=1 minute), and then at t=35 minutes, 2 h, 4 h, 6 h and 24 h post incubation, 70 µL of each mixture was loaded in a 96-well black plate (Sigma Aldrich, France) and fluorescence signal was collected with the IVIS Lumina® LT Series III (PerkinElmer, USA). After excitation at 640 nm (*i.e.,* donor excitation), the donor signal (D) was recovered on the 695-770 nm emission filter and the FRET signal (A) on the 810-875 nm emission filter. Images were processed by using the Living Image software (PerkinElmer, USA). A region of interest, automatically drawn around the wells of the 96-well plates, was used for the signal quantification. Average radiant efficiency values (threshold of 5%) have been used for quantification. The semi-quantitative measurement of the efficiency of the FRET signal (*i.e.,* FRET proximity ratio) was calculated as A/(A+D) ratio. The experiment was repeated twice in duplicate. Results were expressed as mean ± standard error of the mean.

In vitro studies

^{3}H-SQGem uptake in MDA-MB-231 and MCF7 cells.

For cellular uptake studies MDA-MB-231 or MCF-7 cells (250000 cells per well) were seeded in 12-wells plates 24 h prior to the incubation with ^{3}H-SQGem NPs diluted in culture medium (final Gem concentration/well: 10 µM, 0.1 µCi mL^{-1}) for 6 h at 37 °C (n=3). At the end of the incubation period, cells were washed with 1 mL of PBS and then treated with 0.3 mL of 0.25% trypsin for 5 min at 37 °C. The action of trypsin was stopped by adding 1 mL of complete culture medium and the cellular suspension was centrifuged (200 g, 5 min, 4°C), the supernatant discarded and the cells dispersed in 1 mL of PBS. 10 µL of each cellular suspension was mixed with 10 µL of trypan blue (Sigma Aldrich) for cell counting. The

amount of internalized ^{3}H-SQGem was determined using a β-scintillation counter (Beckman Coulter LS6500). Briefly, cell suspensions were first solubilized with 1 mL of Soluene-350 (Perkin Elmer) at 50 °C overnight prior to the addition of 10 mL of Hionic Fluor scintillation cocktail (Perkin Elmer). Finally, samples were vigorously vortexed for 1 minute and kept aside for 2 h prior to the counting.

Statistical analyses

For each data set presented, experiments were performed at least two times. P values in Figure 5 were calculated using one way analysis of variance, with a Tukey's multiple comparison post-test (black asterisks in the figure). P value: *** $P<0.001$. P value in Supplementary Figure 20 was calculated using an unpaired, two-tailed test with Welch's correction (black asterisk in the figure). P value: * $P<0.05$. For data sets obtained using animals, two to six animals were used per group. Plasma and tumor tissue of each animal were processed independently. For data sets obtained using cells at least two experiments were conducted and analysed. For animal experiments, sample size was determined by the minimal number of animals required to obtain statistically significant results. No animal or sample was excluded from the analysis.

Supplementary Figures and tables

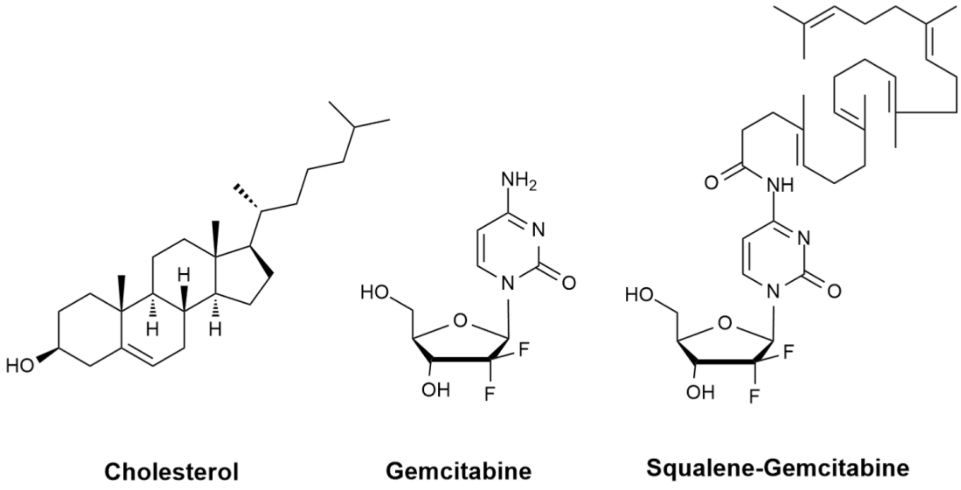

Supplementary Figure 1. Chemical structures of cholesterol, gemcitabine and SQGem.

Chemical structures of cholesterol, gemcitabine and squalene-gemcitabine bioconjugate.

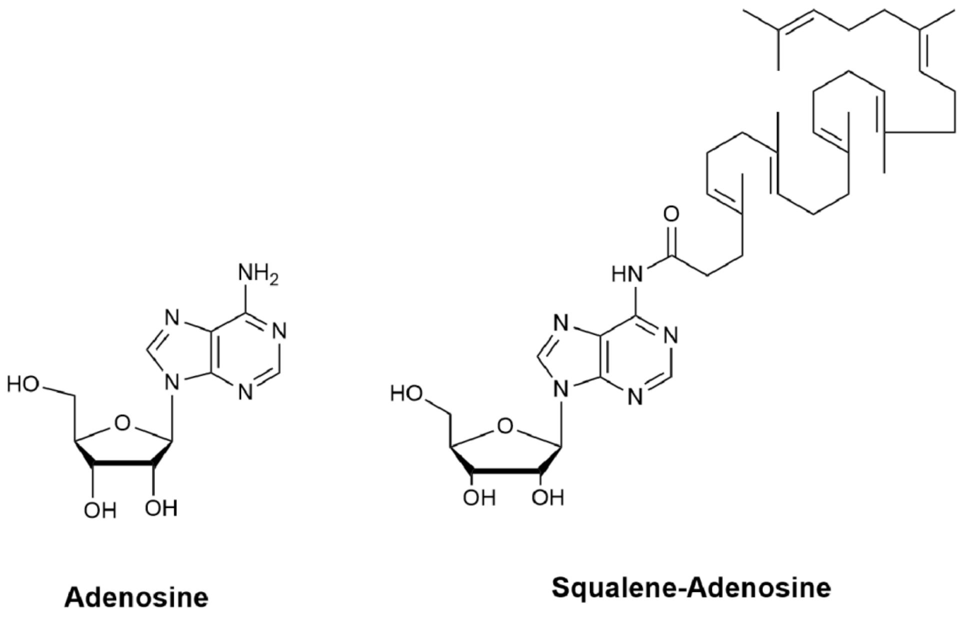

Supplementary Figure 2. Chemical structures of adenosine and squalene-adenosine. Chemical structures of adenosine and squalene-adenosine bioconjugate.

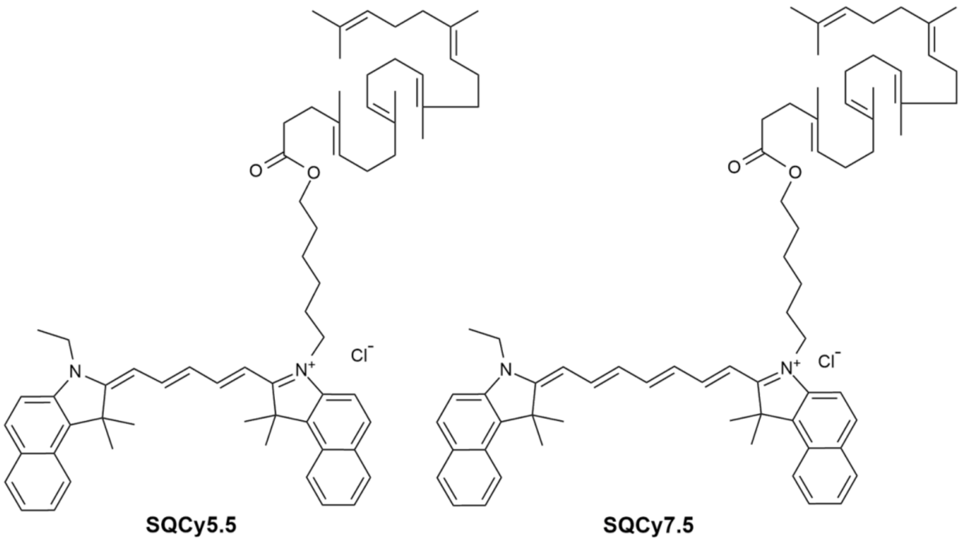

Supplementary Figure 3. Chemical structures of SQCy5.5 and SQ-Cy7.5. Chemical structures of the bioconjugates obtained by coupling the cyanine 5.5 and the cyanine 7.5 to squalene *via* a six-carbon linker.

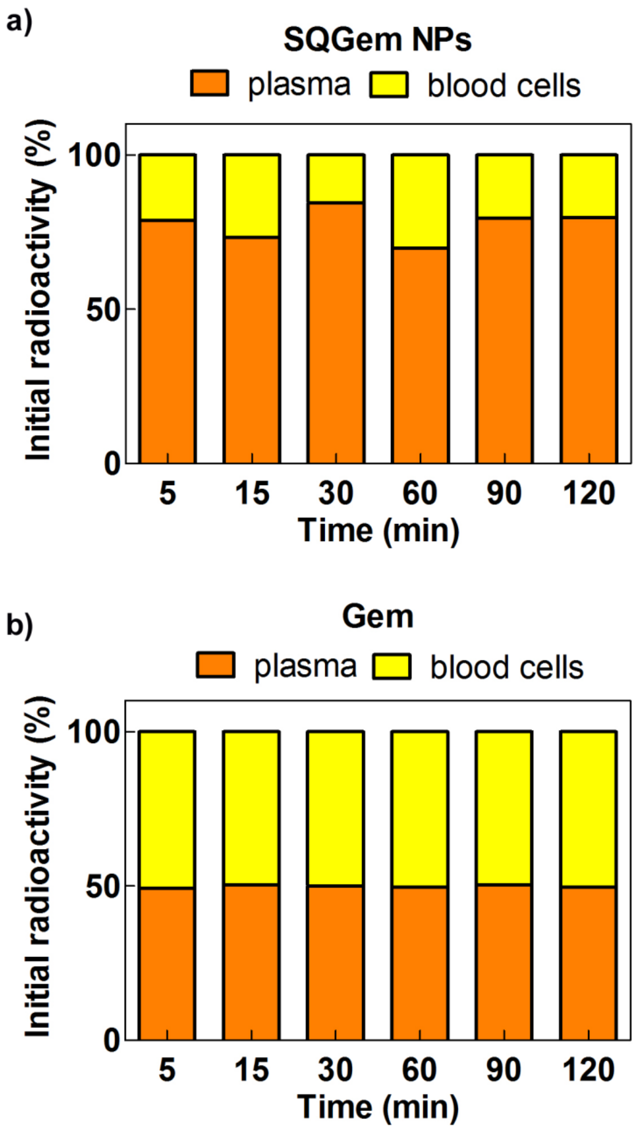

Supplementary Figure 4. ^{3}H-SQGem and ^{3}H-Gem repartition after blood incubation. Repartition of the radioactivity between plasma and blood cells after centrifugation (3000 *g*, 15 min, 15 °C) of human blood previously incubated with (a) ^{3}H-SQGem NPs or (b) free ^{3}H-Gem for 5, 15, 30, 60, 90 or 120 min. Results are expressed as a percentage of total radioactivity initially incubated with blood (mean values).

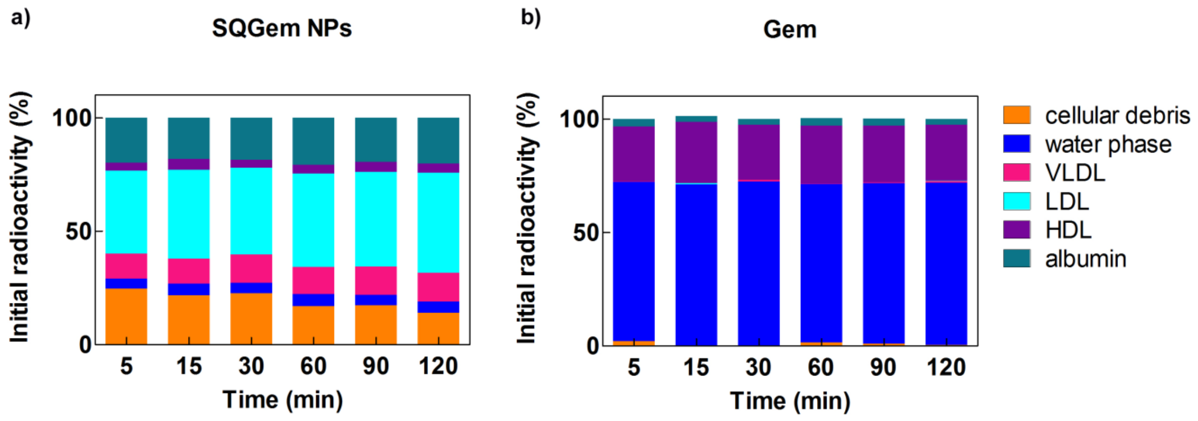

Supplementary Figure 5. ^{3}H-SQGem and ^{3}H-Gem repartition among plasma fractions. Distribution of the radioactivity between the different plasma fractions after incubation of human blood with (a) ^{3}H-SQGem NPs or (b) free ^{3}H-Gem for 5, 15, 30, 60, 90 or 120 min. Results are expressed as a percentage of total plasma radioactivity (mean values).

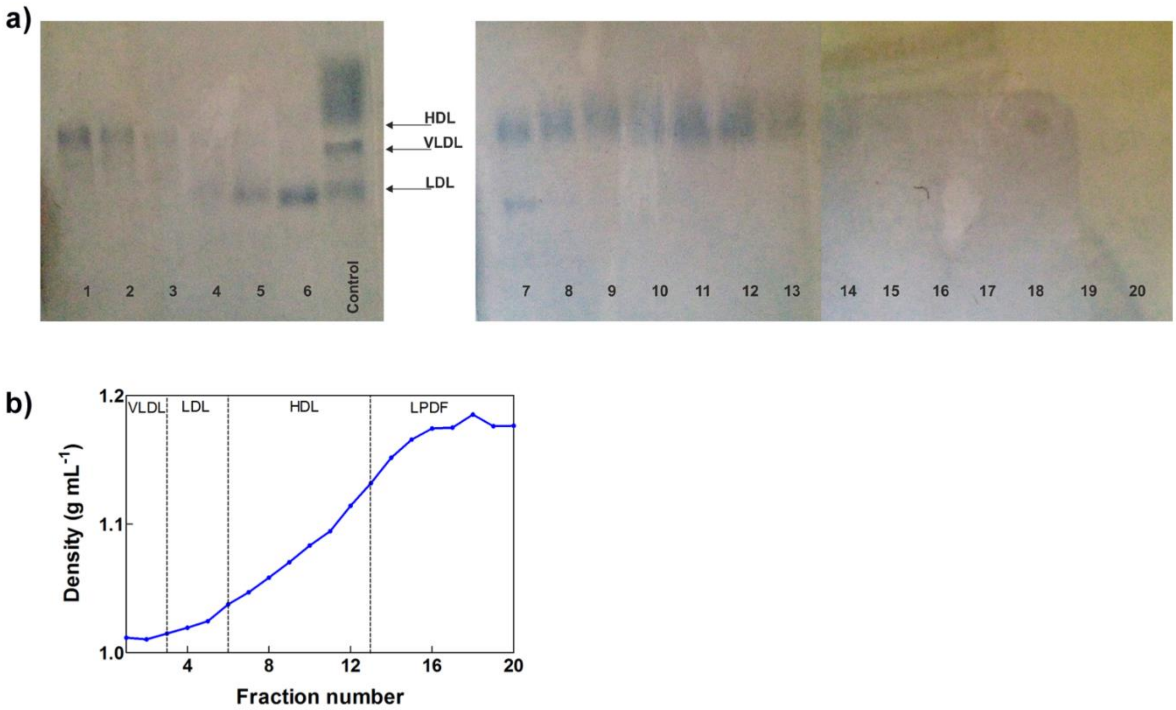

Supplementary Figure 6. Lipoprotein separation by gradient ultracentrifugation. (a) Electrophoresis gels of the twenty fractions separated from rat plasma: VLDL (fractions 1-3), LDL (fractions 4-6), HDL (fractions 7-13) and LPDF with absence of lipoprotein bands (fractions 14-20). (b) Density profile of separated plasma fractions (mean values, n=3).

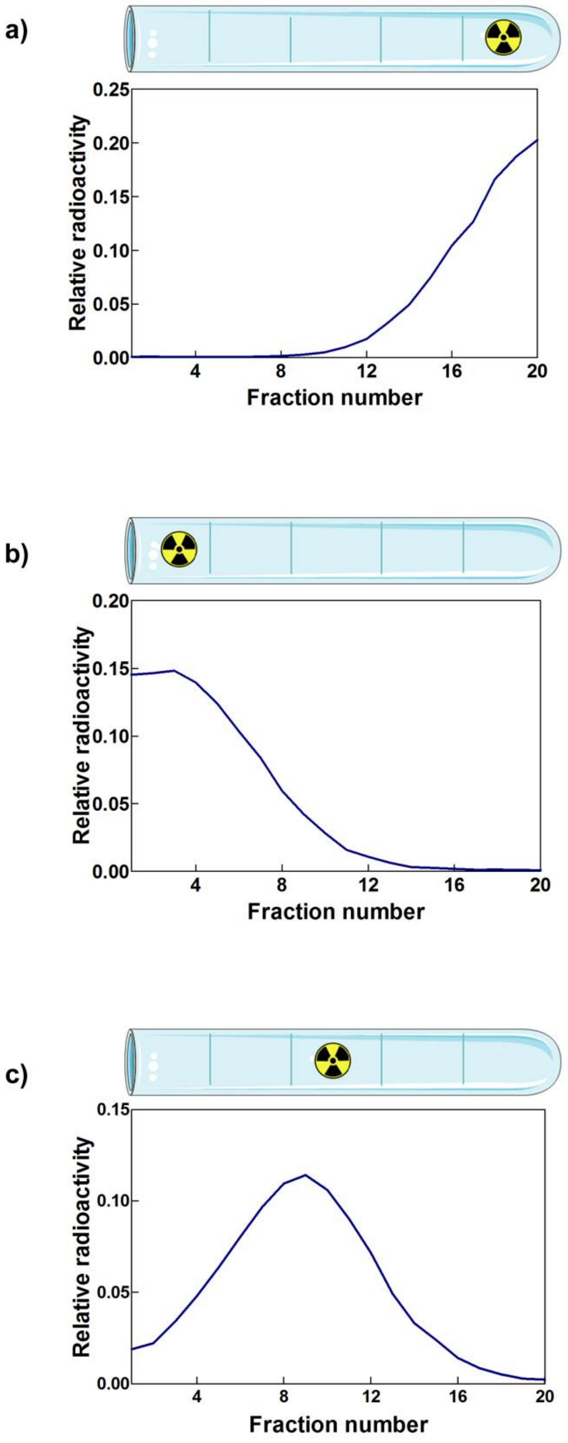

Supplementary Figure 7. ^{3}H-Gem distribution as function of the deposition site. ^{3}H-Gem was incubated with (a) 1.25 g mL^{-1}; (b) 1.006 g mL^{-1} or (c) 1.063 g mL^{-1} NaBr solution for 5 min prior to the ultracentrifugation (mean values, n=3 for (a) and (b) and n=1 for (c)).

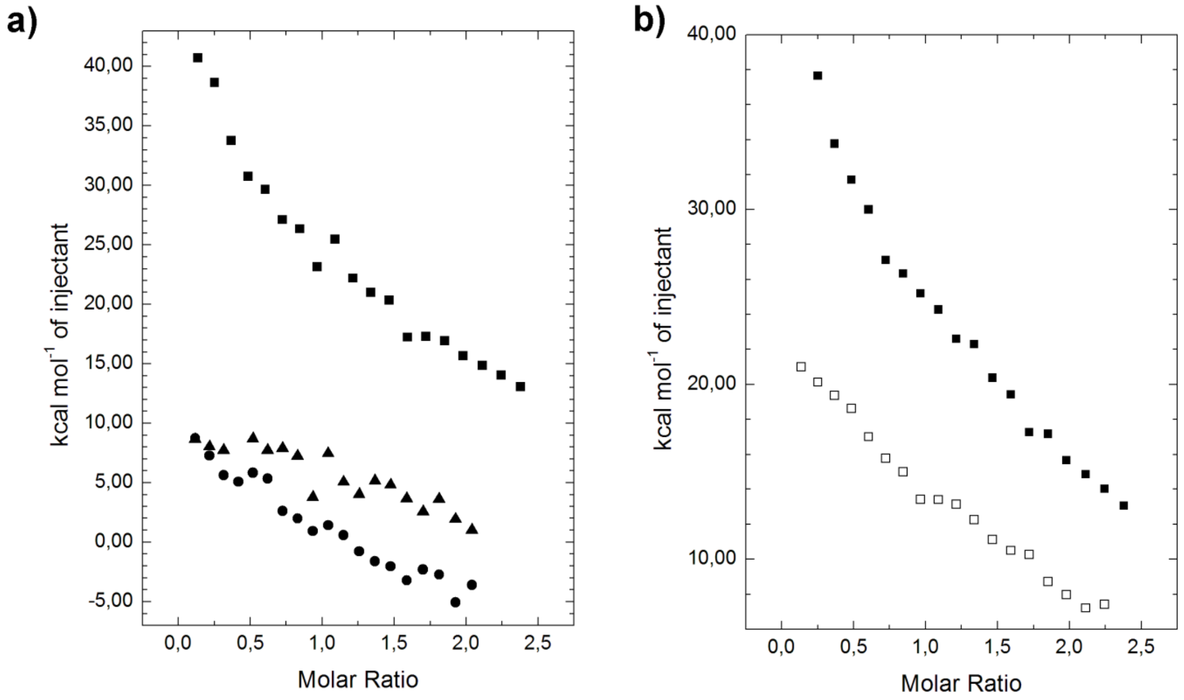

Supplementary Figure 8. **Isothermal titration calorimetry analysis.** Isothermal titration calorimetry analysis of the interaction between the SQGem NPs and lipoproteins or albumin. (a) Integrated binding heats upon injection of SQGem NPs into LDL (solid squares), HDL (solid triangles) dispersions or albumin (solid circles) solution. (b) Integrated binding heats upon injection of SQGem NPs into LDL dispersion (solid squares) or PBS (open squares).

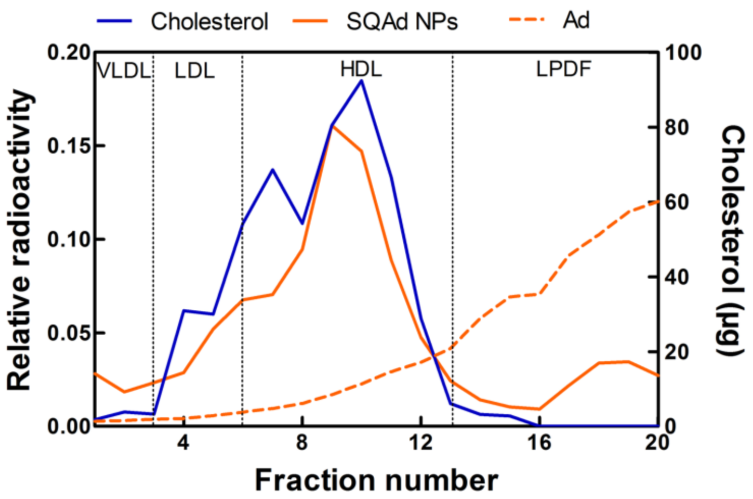

Supplementary Figure 9. ^{3}H-SQAd and ^{3}H-Ad distribution in rat plasmatic fractions. Radioactivity (orange lines) and cholesterol (blue line) distribution among the collected fractions of plasma obtained from rats intravenously injected with ^{3}H-SQAd (solid orange line) or free ^{3}H-Ad (dashed orange line), 5 min post administration. Results are expressed as relative radioactivity compared to total plasma.

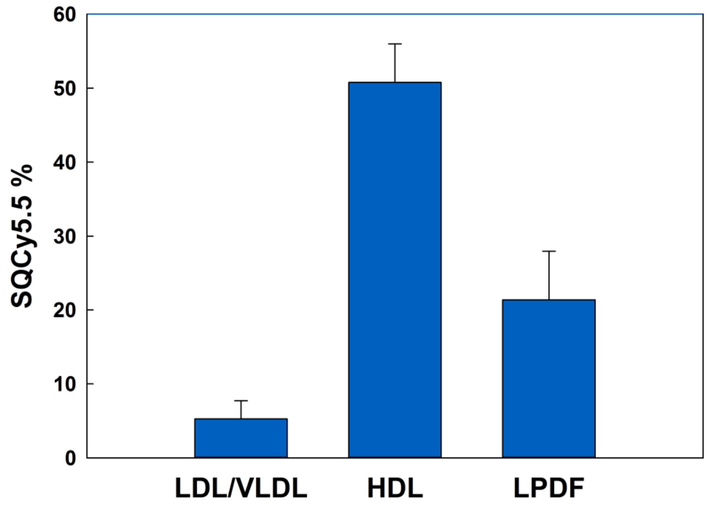

Supplementary Figure 10. **SQCy5.5 distribution in rat plasmatic fraction.** SQCy5.5 distribution of fluorescence among the collected fractions of plasma obtained from rats intravenously injected with SQCy5.5 NPs, 5 min post administration. Results (mean ± standard error of the mean (s.e.m.)) are expressed as relative to the concentration in the whole plasma.

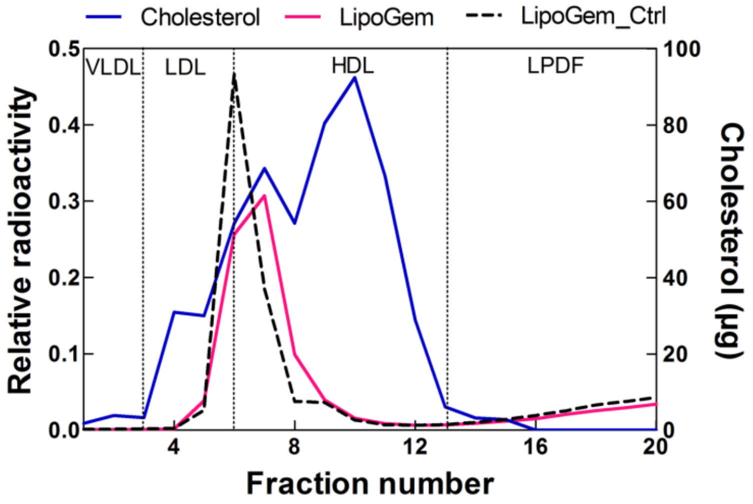

Supplementary Figure 11. ^{3}H-lipoGem distribution in rat plasmatic fractions. Radioactivity (pink line) distribution among the collected fractions of plasma obtained from rats intravenously injected with ^{3}H-LipoGem. Results are expressed as relative radioactivity compared to total plasma. Blue line indicates the cholesterol distribution in Sprague Dawley rats. Black dashed line corresponds to the radioactivity distribution among the collected fractions after gradient ultracentrifugation of the 1.25 g mL^{-1} NaBr solution incubated with ^{3}H-LipoGem.

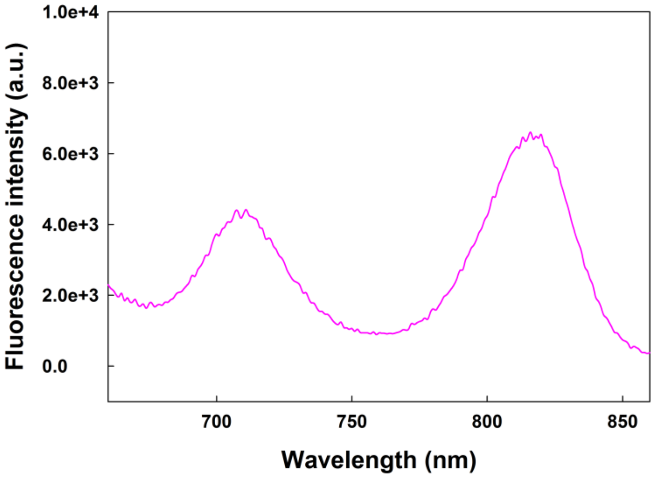

Supplementary Figure 12. Fluorescence emission spectrum of FRET NPs. Fluorescence emission spectrum of FRET SQGem NPs diluted in MilliQ® water (1:26.5) after excitation of the donor at 640 nm.

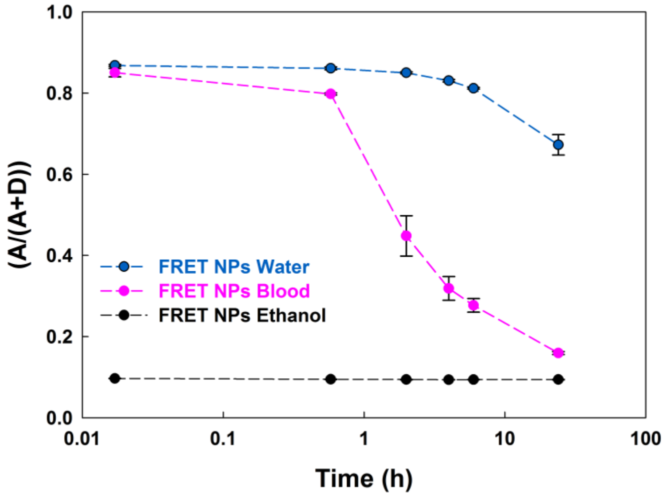

Supplementary Figure 13. *In vitro* stability of FRET NPs. *In vitro* stability study of FRET NPs after dilution with MilliQ® water, rat blood or ethanol. Results are expressed as mean ± standard error of the mean (s.e.m.).

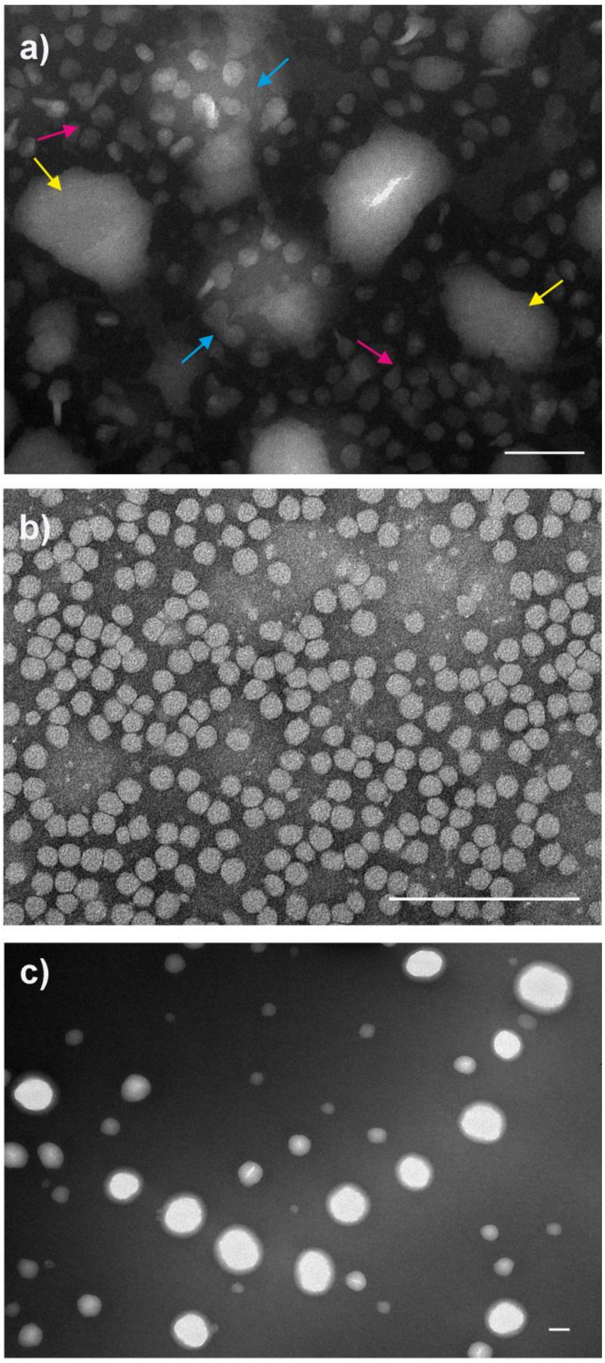

Supplementary Figure 14. TEM images. TEM images of (a) a physical mixture of SQGem NPs (final concentration 500 µg mL^{-1}) and LDL (final concentration 50 µg mL^{-1}) after 5 minutes incubation at 37 °C. Yellow arrows point SQGem NPs, pink arrows point LDL particles and blue arrows point SQGem NPs with LDL particles on their surface (scale bar: 100 nm); (b) LDL dispersion (25 µg mL^{-1}) (scale bar:200 nm) and (c) SQGem NPs (1 mg mL $^{-1}$) (scale bar: 100 nm).

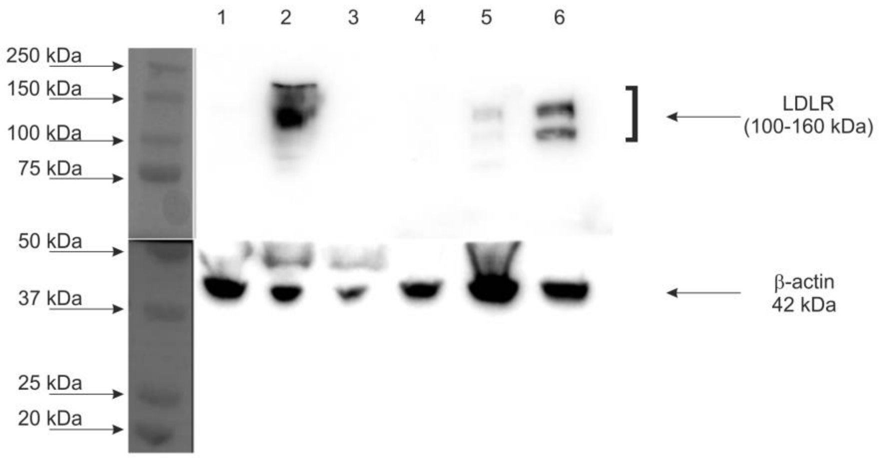

Supplementary Figure 15. LDLR expression in cancer cell lines. Representative Western Blot showing LDL receptors expression in (1) SK-OV-3, (2) MDA-MB-231, (3) A-549, (4) MCF-7, (5) MRC-5 cell lines and (6) MDA-MB-231 tumor xenografts excised from mice. Protein loading was demonstrated by probing for ß-actin. Indicated molecular weight corresponds to the predicted band size. For the LDLR it takes into account the different glycosylated forms of the protein.

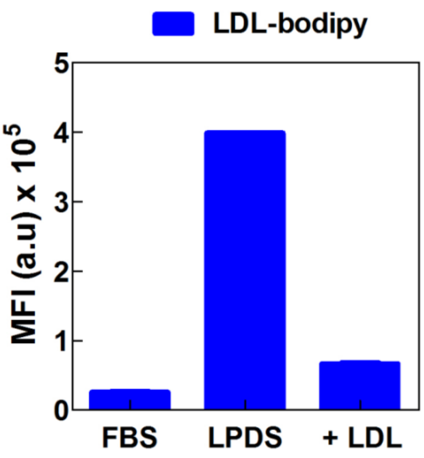

Supplementary Figure 16. Cellular uptake of fluorescently labeled LDL. Cellular uptake of fluorescently labeled LDL (BODIPY® FL LDL) in MDA-MB-231 cells cultured in FBS, in LPDS or preincubated with an excess of LDL in LPDS-supplemented medium at 37 °C. Results are expressed as mean fluorescence intensity (MFI). Bars represent mean values ± s.e.m.

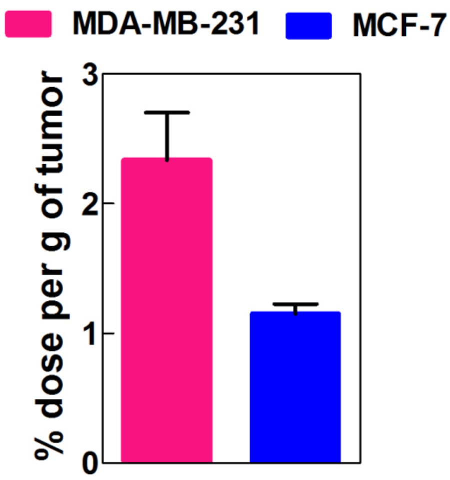

Supplementary Figure 17. Tumor uptake of ^{3}H-SQGem NPs. Radioactivity found in MDA-MB-231 or MCF-7 tumor xenografts excised from athymic nude mice 6 h after treatment with ^{3}H-SQGem NPs (5 mg kg^{-1} eq. Gem). Bars represent means ± s.e.m.

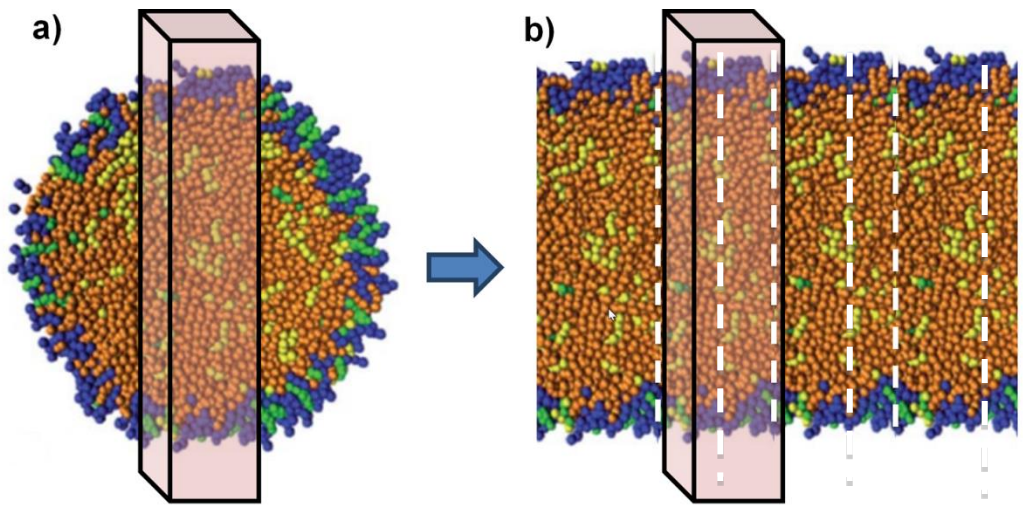

Supplementary Figure 18. Scheme of the simplified LDL lipid core model. (a) Whole spherical lipid droplet of LDL (image adapted from [17]). Black bar shows selected slice of the droplet. (b) This slice was simulated by applying periodic boundary conditions in the same manner as lipid bilayers in MD simulations. White dashed lines show boundaries of individual periodic cells.

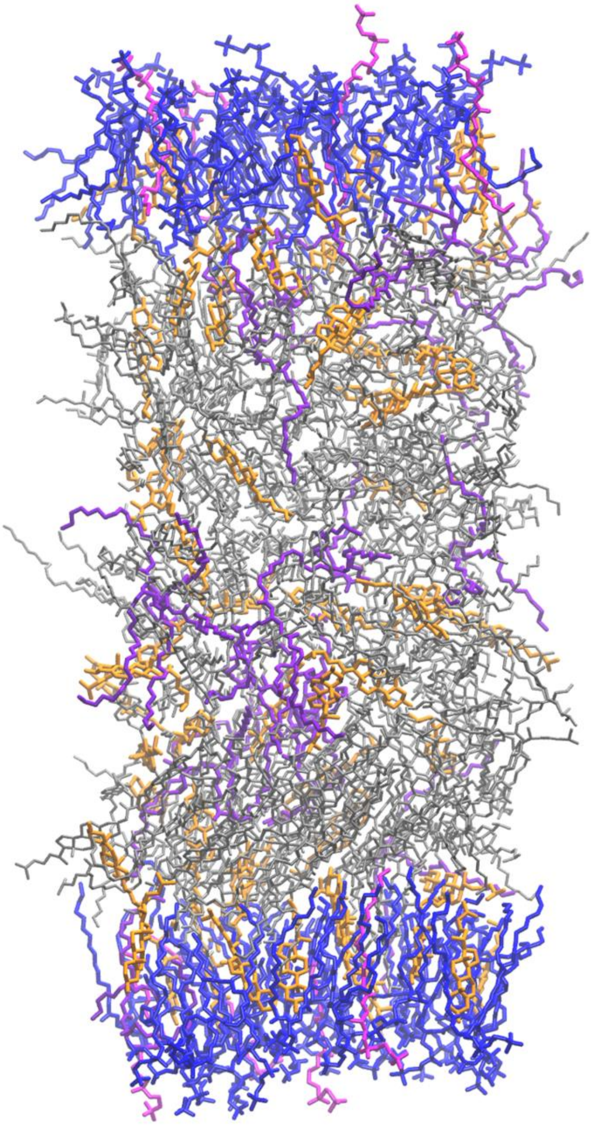

Supplementary Figure 19. Snapshot of equilibrated slice of LDL-like lipid droplet. A single periodic cell is shown. 1-palmitoyl-2-oleoyl-sn-glycero-3-phosphocholine (POPC) lipids are shown in blue, 1- palmitoyl-2-hydroxy-sn-glycero-3-phosphocholine (lyso PC) in magenta, cholesterol in orange, cholesterol oleate in gray and glyceryl trioleate in violet. Water is not shown for clarity. Single periodic cell is shown.

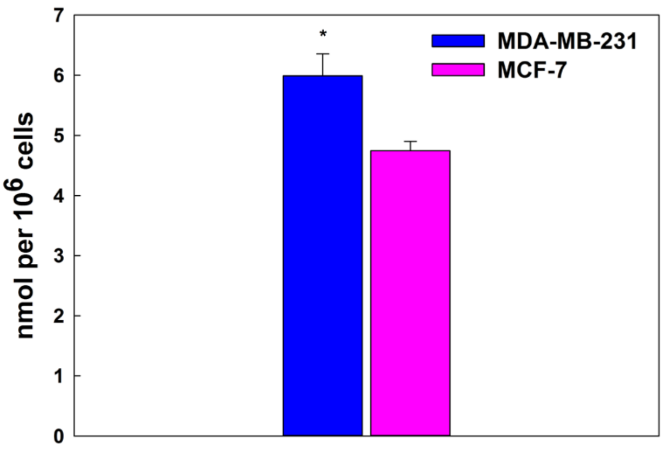

Supplementary Figure 20. ^{3}H-SQGem uptake in MDA-MB-231 and MCF-7 cells. Comparison of ^{3}H-SQGem NPs uptake in MDA-MB-231 and MCF-7 breast cancer cells after 6 h incubation at 37 °C. Results are expressed as nanomoles of Gem per million of cells. Bars represent mean ± standard error of the mean (s.e.m.) (* indicates $p < 0.05$ by unpaired, two-tailed test with Welch's correction).

Supplementary Table 1. Characterization of studied nanoformulations.

Formulation	Mean Diameter [a] (nm)	PdI[a]	Zeta potential (mV)
SQGem NPs	138 ± 9	0.10 ± 0.04	-22 ± 5
^{3}H-SQGem NPs	124 ± 36	0.13 ± 0.06	nd
^{3}H-SQAd NPs	85 ± 20	0.09 ± 0.02	-27 ± 2
SQCy5.5 NPs	120 ± 16	0.07± 0.02	44 ± 4
FRET NPs	139 ± 7	0.09 ± 0.02	-45 ± 7
^{3}H-LipoGem	127 ± 4	0.04 ± 0.03	nd

[a] Determined by dynamic light scattering (DLS).
Results are expressed as mean ± standard deviation (s.d.)

The table above reports the mean diameter, the particle size distribution and the zeta potential of the formulations used in this study.

Supplementary Table 2. Composition of the system used for molecular dynamics simulations.

**Molecule**	**Number**
POPC	64
Lyso PC	8
Cholesterol	60
Cholesterol oleate	160
Glyceryl trioleate	18
Water	4900

The table above reports the composition of the system used for molecular dynamics simulations of LDL. The number of lipid molecules corresponds to 1/10 of the whole LDL particle which has been simulated in coarse-grained simulation[10].

Supplementary References

1 van Tamelen, E. E. & Curphey, T. J. The selective in vitro oxidation of the terminal double bonds in squalene. *Tetrahedron Lett.* **3**, 121-124 (1962).

2 Couvreur, P. *et al.* Squalenoyl Nanomedicines as Potential Therapeutics. *Nano Letters* **6**, 2544-2548 (2006).

3 Reddy, L. H. *et al.* Squalenoylation Favorably Modifies the in Vivo Pharmacokinetics and Biodistribution of Gemcitabine in Mice. *Drug Metab. Dispos.* **36**, 1570-1577 (2008).

4 Gaudin, A. *et al.* Squalenoyl adenosine nanoparticles provide neuroprotection after stroke and spinal cord injury. *Nat. Nanotech.* **9**, 1054-1062 (2014).

5 Simmons, R. L., Yu, R. T. & Myers, A. G. Storable Arylpalladium(II) Reagents for Alkene Labeling in Aqueous Media. *J. Am. Chem. Soc.* **133**, 15870-15873 (2011).

6 Bekkara-Aounallah, F. *et al.* Novel PEGylated Nanoassemblies Made of Self-Assembled Squalenoyl Nucleoside Analogues. *Adv. Func. Mat.* **18**, 3715-3725 (2008).

7 Kirui, D. K. *et al.* Mild hyperthermia enhances transport of liposomal gemcitabine and improves in vivo therapeutic response. *Adv. Healthc. Mater.* **4**, 1092-1103 (2015).

8 Ohnishi, T. *et al.* Frontal analysis of drug–plasma lipoprotein binding using capillary electrophoresis. *J. Pharm. Biomed. Anal.* **27**, 607-614 (2002).

9 Murtola, T. *et al.* Low density lipoprotein: structure, dynamics, and interactions of apoB-100 with lipids. *Soft Matter* **7**, 8135-8141 (2011).

10 Jämbeck, J. P. M. & Lyubartsev, A. P. Another Piece of the Membrane Puzzle: Extending Slipids Further. *J. Chem. Theory Comput.* **9**, 774-784 (2013).

11 Sousa da Silva, A. W. & Vranken, W. F. ACPYPE - AnteChamber PYthon Parser interfacE. *BMC Research Notes* **5**, 367, (2012).

12 Frisch, M. *Gaussian 09.*, (2009).

13 Abraham, M. J. *et al.* GROMACS: High performance molecular simulations through multi-level parallelism from laptops to supercomputers. *SoftwareX* **1–2**, 19-25, (2015).

14 Yesylevskyy, S. O. Pteros: fast and easy to use open-source C++ library for molecular analysis. *J. Comput. Chem.* **33**, 5 (2012).

15 Yesylevskyy, S. O. Pteros 2.0: Evolution of the fast parallel molecular analysis library for C++ and python. *J. Comput. Chem.* **36**, 1480-1488 (2015).

16 Jorgensen, W. L., Chandrasekhar, J., Madura, J. D., Impey, R. W. & Klein, M. L. Comparison of simple potential functions for simulating liquid water. *J. Chem. Phys.* **79**, 926-935 (1983).

17 Bussi, G., Donadio D Fau - Parrinello, M. & Parrinello, M. Canonical sampling through velocity rescaling. *J. Chem. Phys.* **126** (2007).

18 Berendsen, H. J. C., Postma, J. P. M., Gunsteren, W. F. v., DiNola, A. & Haak, J. R. Molecular dynamics with coupling to an external bath. *J. Chem. Phys.* **81**, 3684-3690 (1984).

19 Kumar, S., Rosenberg, J. M., Bouzida, D., Swendsen, R. H. & Kollman, P. A. The weighted histogram analysis method for free-energy calculations on biomolecules. I. The method. *J. Comput. Chem.* **13**, 1011-1021 (1992).
