# Supplementary material for: Conjugation of squalene to gemcitabine as unique approach exploiting endogenous lipoproteins for drug delivery
Source: Nat Commun. 2017 May 30;8:15678. doi: 10.1038/ncomms15678 (PMC5459998; doi:10.1038/ncomms15678)
Supplement: Peer Review File — 2,569 kB [file ncomms15678-s2.pdf]

Reviewer #1 (Remarks to the Author):

The paper submitted by the research group that exploited the use of squalene-based conjugate compounds, reports very interesting results that support the validity of the use of squalene chain as a very simple approach to improve the performance of well-known anticancer drugs. The main results the demonstration that after intravenous administration, nanoparticles made of gemcitabine-squalene strongly interacted with lipopoproteins (LP) which indirectly enabled targeting of cancer cells with high LP receptors expression. This outcome will help the scientific community to consider squalene based nanoparticles as a simple, useful and efficient strategy to overcome all the problems connected with the in vivo use of anticancer compounds.

The high level paper is noteworthy not for the originality but for the importance of the results that have the value to highlight the beneficial effect of creating squalene-based conjugate compounds.

The paper is very clear and the conclusions sounds to be reliable.

The authors are encouraged to consider also:

- Graph Abstract: structure is incorrect, the carbonyl group is missing

- Line 61: "squalenoylation" seems to suggest the introduction of an acyl residue. That is not the case for all the reported squalene-based compounds

- A very recent review on self-assembled NPs is online available doi.org/10.1016/j.drudis.2016.06.018 (Drug Discovery Today)

- Line 355: the authors are citing different papers mainly published by their own research group but other papers are available in the literature: see the Review mentioned before

Reviewer #2 (Remarks to the Author):

When nanoparticles (NPs) systemically administrated into an organism they will immediately interact with variety of biological molecules (proteins, lipids and lipoproteins), which might influence their biological fate and clearance mechanisms.

In this manuscript, the authors show that, after i.v. administration, NPs made of the squalene derivative of the gemcitabine (SQGem) strongly interacted with lipopoproteins (LPs), which indirectly enabled targeting of cancer cells with high LP receptors expression.

The manuscript is well written and interesting however, the claims should be supported by the data. In this version of the manuscript they are still claims without strong, comprehensive evidence for the suggested mechanism.

The concept of designing SQ NPs is not novel and this strong group has published elegant papers in this field including a high profile Nature Nanotech paper a few years ago. Here, they claim that they have insights into the mechanism by which these NPs are taken up and home to cancer cells highly expressing LP receptors.

The work is fairly solid but there is lack of robust, killer experiments to show directly the authors claims.

If the authors can address the concerns below the paper should be published in Nature Comm.

Major concerns that needs to be address prior to publication:

1. The particles bind to HDL in the serum according to the radioactive data. This could be misleading, since the NPs has lipids properties can can be easily found in this fraction anyhow. The authors need to demonstrate selectivity by other means and specificity.

2. In the in vitro experiment - it is better to selectively completely knockout the HDL receptor (or comprehensively silence it) and show it is really the mechanism behind this observation.

3. Many of the experiments are not "strong enough to state the current statement. It is wised to perform a comprehensive proteomic analysis to comprehensively demonstrate that HDL are directly interacting with the SQ NPs.

4. It is also important to understand that other types of NPs made from other materials may not interact the same. Therefore and in order to generalize the concept - it is important to use as control at least two other types of NPs made from different materials and show that they also interact in the same manner.

Reviewer #3 (Remarks to the Author):

The authors have carried out and report here a study of the use of squalene to induce drug insertion into LPs for cancer cell targeting. After intravenous administration, SQ-Gem nanoparticles strongly interacted with lipopoproteins, which may facilitate active targeting due to high LP receptors expression in tumor cells. The topic is interesting. However, the whole study is not well designed and several major points were missed. The paper is premature for publication in Nature Communications. I recommend this paper should be submitted to a more specific journal. And several major concerns need to be addressed before resubmission.

1. Lack of originality innovation. The SQ-Gem conjugate and SQ-Gem NPs has been previously reported (Journal of controlled release, 2010, 147(2): 163-170; Nanomedicine: Nanotechnology, Biology and Medicine, 2011, 7(6): 841-849; Chemical Communications, 2014, 50(40): 5336-5338.). The concept of lipopoproteins involved nanoparticles for potentially targeting tumor cells cannot be considered as a novel drug delivery strategy, which is the inherent advantage of SQ-Gem conjugate and SQ-Gem NPs. This paper is a follow-up study of SQ-Gem NPs, and is more related to the pharmacokinetics of SQ-Gem NPs after intravenous administration, e.g. the distribution of SQGem and Gem in human plasma fractions.

2. The SQ-Gem NPs and 3H-SQ-Gem NPs were not well characterized, for example, the data related to the particle size, size distribution and surface charge status are missing. How will lipopoproteins affect the nanostructure of NPs? e.g. the TEM images of SQ-Gem NPs and lipopoproteins-SQ-Gem NPs. There is only speculation, as the results of in silico simulations. Some aspects of this seem non-intuitive to me.

3. The authors concluded that squalene was chosen because of the lipid nature and its structural similarity with cholesterol, which have good affinity with lipoproteins. Whether cholesterol is a better choice to conjugate with gemcitabine?

4. It is not clear to me what is meant by " In this study, a protein-driven dissociation of SQ-Gem nanoparticles into SQ-Gem monomers was demonstrated to occur before cell capture" (line 267 and 268, p 13). Please provide convincing interpretations, maybe with more evidence. If the SQ-Gem NPs were dissociated before cell uptake, how can lipopoproteins facilitate tumor cell targeting? In addition, if the SQ-Gem NPs will be dissociated in blood, what's the advantage of SQ-Gem NPs over SQ-Gem conjugates? Will the PEGylated SQ-Gem NPs also be dissociated in blood? If not, which one is a better formulation for drug delivery, PEGylated SQ-Gem NPs or non-PEGylated SQ-Gem NPs?

5. The organ distribution and the systematic pharmacokinetics profile of SQ-Gem NPs should be examined. How will lipopoproteins in vivo affect the biodistribution in organs and the pharmacokinetics of SQ-Gem NPs?

6. The authors conclude that "It was discovered that endogenous LDL particles may function as carriers for SQ-Gem, thus allowing the indirect targeting of cancer cells displaying high expression and activity of LDL receptors, without the need to functionalize NPs surface with hydrophilic PEG (polyethylene glycol) chains and/or with specific ligands." Actually, PEGylation cannot facilitate the cellular uptake of NPs, even hinder the cellular uptake, but benefit the long circulation in blood. Moreover, the authors didn't set the PEGylated SQ-Gem NPs as a control. How can they draw this conclusion?

7. The manuscript should be carefully checked for grammar mistakes.

INSTITUT GALIEN PARIS-SUD – UMR CNRS 8612

5, rue Jean-Baptiste Clément
92296 Châtenay-Malabry cedex - France

Answers to the reviewers

Reviewers' comments:

Reviewer #1:

The paper submitted by the research group that exploited the use of squalene-based conjugate compounds, reports very interesting results that support the validity of the use of squalene chain as a very simple approach to improve the performance of well-known anticancer drugs. The main results the demonstration that after intravenous administration, nanoparticles made of gemcitabine-squalene strongly interacted with lipopoproteins (LP) which indirectly enabled targeting of cancer cells with high LP receptors expression. This outcome will help the scientific community to consider squalene based nanoparticles as a simple, useful and efficient strategy to overcome all the problems connected with the in vivo use of anticancer compounds.

The high level paper is noteworthy not for the originality but for the importance of the results that have the value to highlight the beneficial effect of creating squalene-based conjugate compounds.

The paper is very clear and the conclusions sounds to be reliable.

The authors are encouraged to consider also:

- Graph Abstract: structure is incorrect, the carbonyl group is missing

Answer: As suggested by the reviewer the graphical abstract has been opportunely corrected. (Revised manuscript page 3)

- Line 61: "squalenoylation" seems to suggest the introduction of an acyl residue. That is not the case for all the reported squalene-based compounds

Answer: We agree with the reviewer that the term "*squalenoylation*" seems to suggest the introduction of an acyl residue, although it does not occur in all the squalene derivatives. Accordingly, in order to avoid any confusion, the term "*squalenoylation*" has been removed from all the manuscript and the sentences containing this term have been reformulated (Revised manuscript: pages 4 and 22). The title has been also modified as follows: "*Conjugation of squalene to gemcitabine, a unique approach which exploits endogenous lipoproteins for drug delivery*".

- A very recent review on self-assembled NPs is online available doi.org/10.1016/j.drudis.2016.06.018 (Drug Discovery Today)

Answer: As suggested by the reviewer, the reference "Fumagalli G. et al., Self-assembly drug conjugates for anticancer treatment, *Drug discovery today*, **2016**, 21 (8) 1321, has been added in the revised version: Manuscript (Reference 8, pages 4 and 31).

- Line 355: the authors are citing different papers mainly published by their own research group but other papers are available in the literature: see the Review mentioned before

Answer: As suggested by the reviewer, the following new references related to other studies on squalene bioconjugates have been added in the revised version: Manuscript (References 51, 52 and 53, pages 22 and 35)

Sarpietro MG *et al.*, Squalenoyl prodrug of paclitaxel: synthesis and evaluation of its incorporation in phospholipid bilayers *Int J Pharm*. **2012** 436(1-2):135-40

Dosio F *et al.*, Novel nanoassemblies composed of squalenoyl-paclitaxel derivatives: synthesis, characterization, and biological evaluation. *Bioconjug Chem*. **2010**;21 (7):1349-61

Valetti, S. *et al.* Peptide-functionalized nanoparticles for selective targeting of pancreatic tumor. *J. Control. Release* **2014** 192, 29–39

The authors thank the reviewer for the positive comments.

Reviewer #2:

When nanoparticles (NPs) systemically administrated into an organism they will immediately interact with variety of biological molecules (proteins, lipids and lipoproteins), which might influence their biological fate and clearance mechanisms.
In this manuscript, the authors show that, after i.v. administration, NPs made of the squalene derivative of the gemcitabine (SQGem) strongly interacted with lipopoproteins (LPs), which indirectly enabled targeting of cancer cells with high LP receptors expression.
The manuscript is well written and interesting however, the claims should be supported by the data. In this version of the manuscript they are still claims without strong, comprehensive evidence for the suggested mechanism.
The concept of designing SQ NPs is not novel and this strong group has published elegant papers in this field including a high profile Nature Nanotech paper a few years ago. Here, they claim that they have insights into the mechanism by which these NPs are taken up and home to cancer cells highly expressing LP receptors.
The work is fairly solid but there is lack of robust, killer experiments to show directly the authors claims.
If the authors can address the concerns below the paper should be published in Nature Comm.

Major concerns that needs to be address prior to publication:
1. The particles bind to HDL in the serum according to the radioactive data. This could be misleading, since the NPs has lipids properties can can be easily found in this fraction anyhow. The authors need to demonstrate selectivity by other means and specificity.

Answer: As suggested by the reviewer additional experiments have been performed to further demonstrate the specific interaction between SQGem and the lipoproteins.
First, to check if NPs can be (or not) easily found in the same fraction anyhow, we have carried out a control ultracentrifugation in NaBr density gradient. Briefly, ^{3}H-SQGem NPs have been added to a solution of 1.25 g mL^{-1} NaBr and incubated at 37 °C for 5 minutes. Then, 1 mL of this mixture has been placed at the bottom of a centrifuge tube (exactly in the same conditions than with plasma collected from rats 5 minutes post iv administration of ^{3}H-SQGem NPs). It was clearly observed that NPs were not stable in the 1.25 g mL^{-1} NaBr solution since they rapidly formed a white precipitate. Following the ultracentrifugation, this precipitate localized at the level of the fraction 12. On the contrary, when the experiment was performed with plasma collected after intravenous administration of the ^{3}H-SQGem NPs to rats, the peak of radioactivity corresponded to the fractions 8-11, containing also the highest amount of cholesterol and precipitates were never observed. (Revised manuscript: Figure 4, page 11). These results highlight that the localization of the radioactivity in the HDL fractions is the result of a specific interaction and did not result from an artifact due to NPs density.

The specificity of the interaction between SQGem NPs and LDL was then confirmed by isothermal titration calorimetry experiments performed with human lipoproteins separated from the blood of healthy volunteers. The heat flows were determined when SQGem NPs were added to LDL dispersion, HDL dispersion or albumin solution placed in the titration cell. ITC thermograms revealed the existence of a strong interaction between the SQGem and the LDL, while it was not observed either with HDL or with albumin. The specificity of the recorded signal was confirmed after dilution of SQGem NPs in PBS (Supplementary Fig. S8). These results are in agreement with those obtained after incubation of the SQGem NPs with human blood (Revised Manuscript: Figure 1, page 8) and provide a further proof of the existence of a specific interaction with the human LDL fraction.

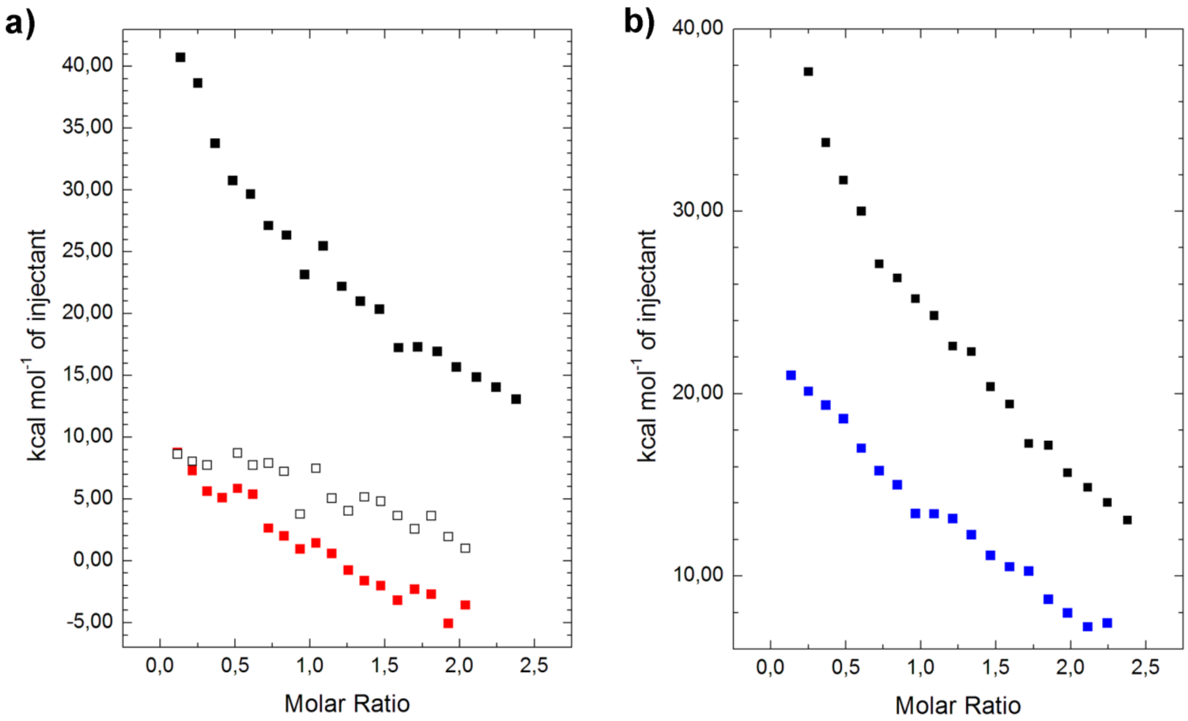

Supplementary Fig. S8. **Isothermal titration calorimetry analysis** Isothermal titration calorimetry analysis (n=2) of the interaction between the SQGem NPs and lipoproteins or albumin. (a) Integrated binding heats upon injection of SQGem NPs into LDL (solid squares), HDL (open squares) dispersions or albumin (red squares) solution. (b) Integrated binding heats upon injection of SQGem NPs into LDL dispersion (solid squares) or PBS (blue squares).

These results have been added in the revised version: Manuscript (pages 9 and 16-17); Supplementary Information (page 18); Supplementary Fig. S8.

2. In the in vitro experiment - it is better to selectively completely knockout the HDL receptor (or comprehensively silence it) and show it is really the mechanism behind this observation.

Answer: All the in vitro cell culture experiments have been performed on human cancer cells and not in rodent cancer cells. Since LDLR is involved in cholesterol uptake in humans and to meet the reviewer recommendation, we have looked for LDLR knockout human cells. Unfortunately, we failed to obtain cells displaying complete inhibition of LDL receptor expression. In addition, it was mandatory to have a cell line allowing relevant comparison with MDA-MB-231 used in this article. Nevertheless, with the aim to further support the hypothesis, we have performed a supplementary experiment and have investigated the contribution of the LDLR in the uptake of ^{3}H-SQGem NPs on MDA-MB-231 and MCF-7 breast cancer cells, since those cells share similar features (they are both human breast adenocarcinoma cells) but display high and low level of LDLR expression, respectively (Supplementary Fig. S16). After 6 h of incubation with ^{3}H-SQGem NPs at 37 °C, a higher radioactivity signal was detected in MDA-MB-231 cells (high expression of LDLR) compared to the MCF-7 ones (low expression of LDLR) (Supplementary Fig. S21). These results showed that a higher LDLR expression induced greater uptake which confirmed the key role of the LDLR in the cell internalization of the SQGem bioconjugates.

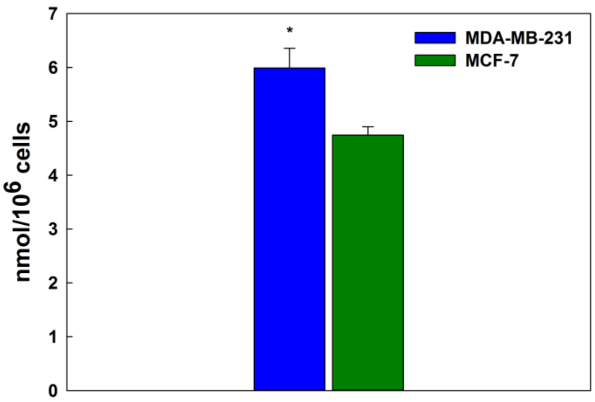

Supplementary Fig. S21 ^{3}H-SQGem uptake in MDA-MB-231 and MCF-7 cells. Comparison of ^{3}H-SQGem NPs uptake in MDA-MB-231 and MCF-7 breast cancer cells after 6 h incubation at 37 °C. Results are expressed as nanomoles of Gem per million of cells. Bars represent mean ± standard error of the mean (s.e.m.) (* indicates $p < 0.05$ by unpaired, two-tailed test with Welch's correction, n=3).

These results have been added in the revised version: Manuscript (pages 16 and 21-22); Supplementary Information (page 22); Supplementary Fig. S21.

3. Many of the experiments are not "strong enough to state the current statement. It is wised to perform a comprehensive proteomic analysis to comprehensively demonstrate that HDL are directly interacting with the SQ NPs.

Answer: As suggested by the reviewer, we have performed proteomic analysis to identify and quantify the proteins adsorbed at the nanoparticles surface.

The commonly used experimental setting requires the incubation of NPs with plasma, several washing steps and finally, the desorption of the proteins by treatment with dithiothreitol and SDS solution (2 h, 60 °C). However, when this protocol has been applied to the SQGem NPs (n=2) no trace of protein corona was detected (see lines 2 and 5 in the figure below, which clearly showed the absence of protein corona after incubation of SQGem NPs with rat plasma. Lines 3 and 6 correspond to the total plasma proteins, while lines 1 and 4 correspond to the HDL fraction)

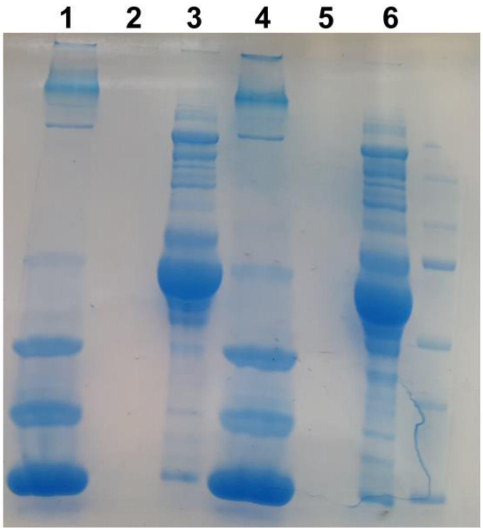

1) Rat 1 HDL
2) Protein corona (SQGem NPs/plasma rat 1)
3) Rat 1 Plasma
4) Rat 2 HDL
5) Protein corona (SQGem NPs/plasma rat 2)
6) Rat 2 Plasma

These results are in agreement with our previous conclusion that the squalene moiety drives the insertion of SQGem molecules into LDL, which supposes that SQGem NPs undergo first a quick disassembly in plasma. As a result of the NPs loss of integrity, no stable protein adsorption at the surface of the SQGem NPs with subsequent formation of a protein "corona" could occur.
Thus, we have further investigated the disassembly of the SQGem NPs in blood by performing new experiments with FRET SQGem NPs. These NPs have been prepared by labelling SQGem NPs with the squalene derivatives of the cyanine 5.5 (SQCy5.5, 0.6% w/w) and the cyanine 7.5 (SQCy7.5, 0.6% w/w), which behave as FRET donor and acceptor, respectively. For details concerning the preparation of these FRET nanoparticles, see Supplementary Information pages 13-14. Fluorescence emission spectrum of these FRET NPs is reported in Supplementary Fig. S12. Taking advantage of the dependence of FRET signal on the distance between the FRET pair (SQCy5.5/SQCy7.5), this approach enabled to monitor the integrity of SQGem NPs over time.
Stability has been evaluated at 37 °C after opportune dilution of NPs in (i) water, (ii) rat blood and (iii) ethanol where SQGem is soluble. Thus, the dilution in ethanol has been used as a control of the complete disassembly of FRET SQGem NPs and consequently absence of energy transfer between the donor and acceptor dyes. Samples were imaged at regular times post incubation using the IVIS Lumina imaging system. After excitation at 640 nm (SQCy5.5 excitation wavelength), the emission was collected at 695-770 nm (donor signal, D) and 810-875 nm (FRET signal, A). Following signal quantification, the FRET proximity ratio, providing a semiquantitative measurement of the FRET efficiency has been calculated according to the equation A/(A+D).(Preus, S. & Wilhelmsson, L. M.. *Chembiochem*, **2012**, 13, 1990-2001)

While NPs were stable in water up to 24 h, the rapid drop of the FRET signal in blood clearly indicated a fast disassembly of the NPs in this medium. (Supplementary Fig. S13). These results explain the absence of the protein corona after proteomic analysis and support the hypothesis that the interaction between SQGem NPs and lipoproteins involves SQGem molecules and not intact nanoparticles.

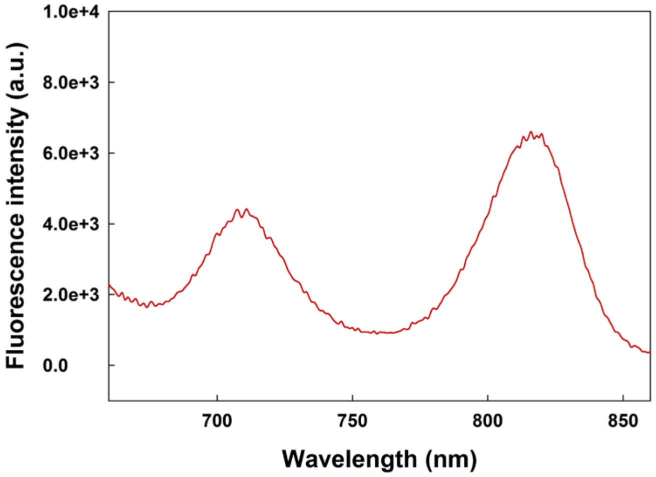

Supplementary Fig. S12: Fluorescence emission spectrum of FRET NPs. Fluorescence emission spectra of FRET SQGem NPs, diluted in MilliQ® water (1:26.5) after excitation of the donor at 640nm

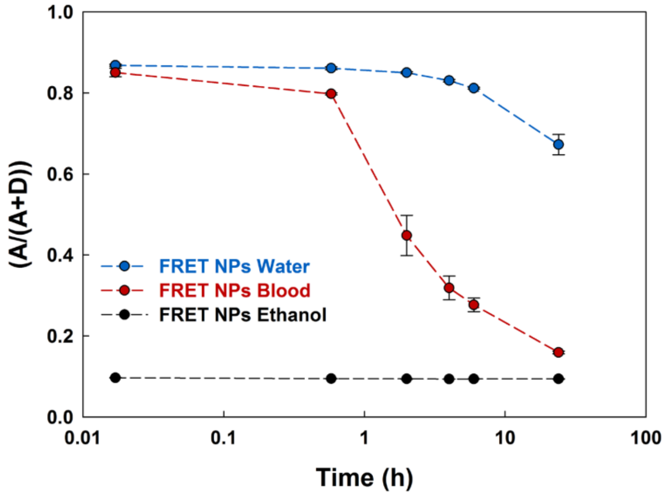

Supplementary Fig. S13. *In vitro* stability of FRET NPs.*In vitro* stability study of FRET NPs after dilution with (i) MilliQ® water, (ii) rat blood or (iii) ethanol. Results are expressed as mean ± standard error of the mean (s.e.m.) (n=2).

These new data have been added in the revised version: Manuscript (page 14); Supplementary Information (pages 9-14 and 21); Supplementary Fig. S12 and S13.

Finally, we have investigated the interaction between SQGem NPs and lipoproteins by transmission electron microscopy. Briefly, SQGem NPs have been incubated with human LDL for 5 minutes at 37 °C and then images of this physical mixture have been acquired. SQGem NPs only and LDL particles only have been used as controls. In the physical mixture, shape modified SQGem NPs, possibly in the course of a disassembled process (yellow arrows), and LDL particles (pink arrows) are visible but also SQGem NPs displaying lipoproteins on their surface (blue arrows).

(Supplementary Fig. S15) This microscopy image reveals how the first encounter between SQGem NPs and LDL occurs. Of note, it provides only a static image, while the interaction between the SQGem and lipoproteins is rather a dynamic phenomenon. Indeed, as shown in the FRET experiments detailed above, the establishment of this preliminary interaction is followed by the disassembly of the SQGem NPs and by the release of single SQGem bioconjugates that will further insert into the lipoproteins (which is obviously not observable by TEM).

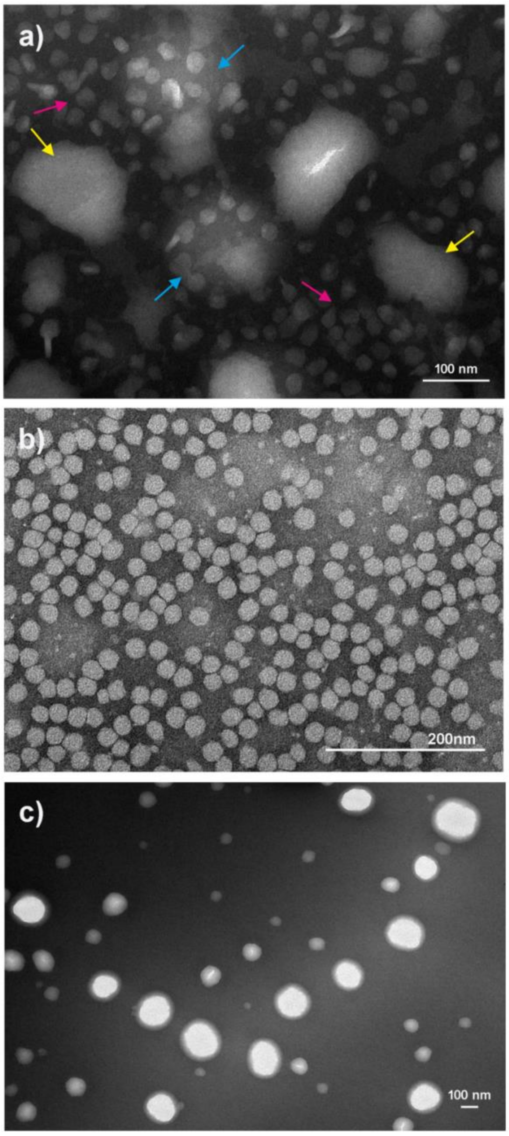

Supplementary Fig. S15. TEM images. TEM images of (a) physical mixture of SQGem NPs (final concentration 500 µg mL^{-1}) and LDL (final concentration 50 µg.mL^{-1}) after 5 minutes incubation at 37°C. Yellow arrows point SQGem NPs, pink arrows point LDL particles and blue arrows point SQGem NPs with LDL particles on their surface; (b) LDL dispersion (25 µg.mL^{-1}) and (c) SQGem NPs (1 mg.mL^{-1})

These new results have been added in the revised version: Manuscript (pages 17-18); Supplementary Information (page 19); Supplementary Fig. S15.

In conclusion, all these additional experiments using ITC, Proteomics, FRET and Transmission Electron Microscopy converge to demonstrate the interaction between SQGem and HDL/LDL.

4. It is also important to understand that other types of NPs made from other materials may not interact the same. Therefore and in order to generalize the concept - it is important to use as control at least two other types of NPs made from different materials and show that they also interact in the same manner.

Answer: As suggested by the reviewer additional experiments have been performed in order to demonstrate that *(i)* the interaction with the lipoproteins is a feature common to different squalene-based nanoparticles and not restricted to SQGem NPs and that (*ii*) the lipidic nature of a drug nanocarrier does not imply the capacity to establish an interaction with circulating lipoproteins.

Thus, with the aim of generalizing the proposed approach, we have investigated the behavior of nanoparticles made by the self-assembly of two other squalene derivatives: ^{3}H-Squalene-adenosine (^{3}H-SQAd) NPs (chosen because of the already demonstrated pharmacological activity of SQAd in stroke, see Gaudin et al., Nature Nanotechnology, 2014) and Squalene-Cy5.5 (SQCy5.5) NPs (chosen because this squalene conjugate allowed another analytical detection, ie. fluorescence). The interaction with lipoproteins of these two structurally different squalene derivatives has been determined by using the same experimental setting already established for SQGem NPs. Thus, ^{3}H-SQAd NPs and SQCy5.5 NPs were intravenously injected to rats and their distribution among plasma fractions was assessed.
First, it was observed that the distribution profile of ^{3}H-SQAd NPs perfectly overlapped that already observed after administration of SQGem NPs, and the highest percentage of ^{3}H-SQAd has been found in the HDL fraction (63%), followed by the LDL (15%) and the VLDL (7%) fractions. Only 15% of the ^{3}H-SQAd distributed in the LPDF fraction. Similarly to the free gemcitabine, also the free adenosine did not interact significantly with the lipoproteins and was mainly recovered at the bottom of the tube, in the LPDF. As already observed with free gemcitabine (Supplementary Fig. S7), this localization could be attributed to the physico-chemical properties of the molecule, rather than to interactions with soluble plasma proteins.
These new results have been added in the revised version: Manuscript (pages 12 and 20); Supplementary Information pages 7-9, 12-13 and 19-20); Supplementary Fig. S9.

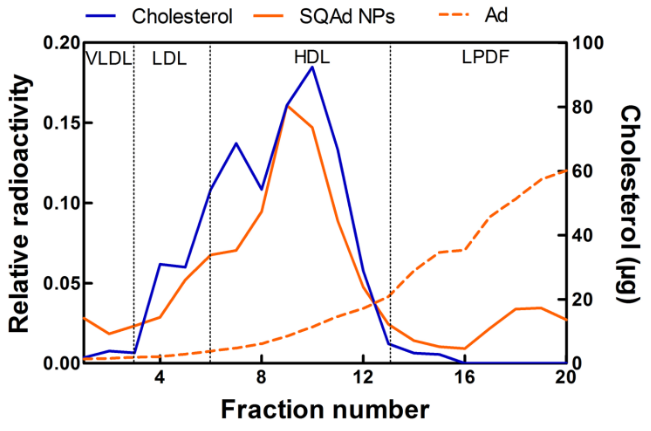

Supplementary Fig. S9. ^{3}H-SQAd and ^{3}H-Ad distribution in plasmatic fractions – in vivo. Radioactivity (orange lines) and cholesterol (blue line) distribution among the collected fractions of plasma obtained from rats intravenously injected with ^{3}H-SQAd (solid orange line, n=4) or free ^{3}H-Ad (dashed orange line, n=2), 5 min post administration. Results are expressed as relative radioactivity compared to total plasma.

Secondly, the administration of SQCy5.5 NPs resulted also in comparable distribution among the plasma fractions, thus confirming that the conjugation to squalene led to a specific interaction with lipoproteins. In this case, due to detection limits, quantification has been carried out in the pool of fractions corresponding to LDL/VLDL (fractions 1-6), HDL (fractions 7-13) and LPDF (fractions 14-20). The highest % of SQCy5.5 (expressed as relative to the concentration in the whole plasma) has been found again in the HDL fractions (50%). (Supplementary Fig. S10)

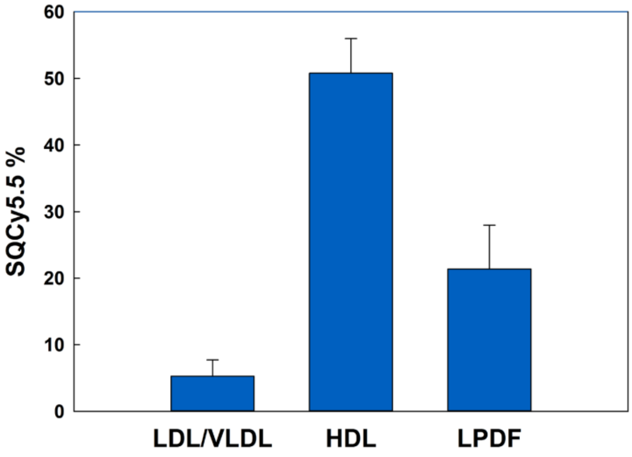

Supplementary Fig. S10. SQCy5.5 distribution in plasmatic fractions – in vivo. SQCy5.5 distribution of fluorescence among the collected fractions of plasma obtained from rats intravenously injected with SQCy5.5 NPs, 5 min post administration (n=4). Results (mean ± standard error of the mean (s.e.m.)) are expressed as relative to the concentration in the whole plasma.

These new results have been added in the revised version: Manuscript (pages 13 and 20), Supplementary Information (pages 9-12 and 20), Supplementary Fig. S10.

On the whole, these results clearly demonstrated that the interaction with the HDL fraction, the main cholesterol transporting particles in rats, was not exclusive of SQGem but represented a more general concept common to different squalene derivatives, independently of the molecule conjugated to the squalene.

Nevertheless, as suggested by the reviewer, we additionally investigated whether such a specific interaction with the major cholesterol transporting particles in rats could also occur (or not) with other types of nanocarriers. Thus, we have prepared ^{3}H-Gemcitabine loaded liposomes (^{3}H-LipoGem) and we have administered them intravenously to rats. Following the same experimental protocol as already described for ^{3}H-SQGem NPs, ^{3}H- SQAd NPs and SQCy5.5 NPs, blood has been collected, five minutes after injection, by cardiac puncture and the different plasma fractions separated by an ultracentrifugation in NaBr density gradients. The majority of the radioactivity, expressed as relative to the total amount in plasma, was found in the fractions 5-8, which could a priori correspond to the HDL1 subpopulation, also suggesting an interaction of liposomes with this subpopulation.
However, very importantly, a control experiment carried out by replacing the plasma with a 1.25 g mL^{-1} NaBr solution incubated for 5 minutes with ^{3}H-GemLipo, revealed that ^{3}H-GemLipo accumulated in the same 5-8 fractions even in the absence of any lipoprotein. In addition, it was observed that, contrarily to the different squalene derivatives, the LipoGem only partly overlapped with the cholesterol content in the different fractions. Accordingly, it was concluded that the distribution of ^{3}H-GemLipo in fractions 5-8 simply resulted from their accumulation in the region of the gradient which displayed the same density than the liposomal formulation and was not a consequence of the interaction with the lipoproteins. (Supplementary Fig. S11).

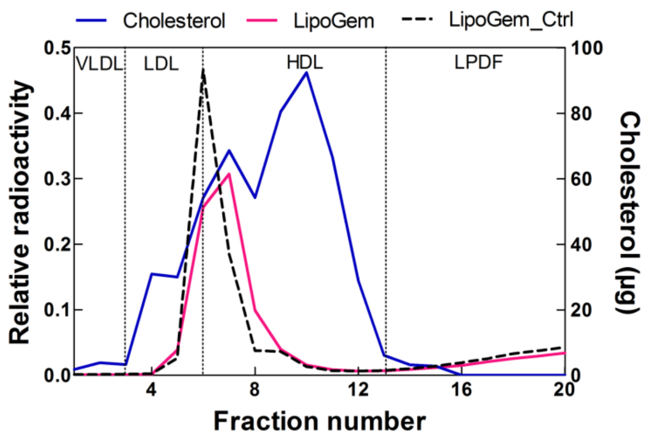

Supplementary Fig. S11. ^{3}H-lipoGem distribution in plasmatic fractions – in vivo. Radioactivity (pink line) distribution among the collected fractions of plasma obtained from rats intravenously injected with ^{3}H-LipoGem (n=4). Results are expressed as relative radioactivity compared to total plasma. Blue line indicates the cholesterol distribution in Sprague Dawley rats. Black dashed line corresponds to the radioactivity distribution among the collected fractions after gradient ultracentrifugation of the 1.25 g mL^{-1} NaBr solution incubated with ^{3}H-LipoGem (Control experiment, n=2)

These new results have been added in the revised version: Manuscript (pages 13 and 20); Supplementary Information pages 14-15 and 20); Supplementary Fig. S11.

In a nutshell, all these additional experiments confirmed that the interaction of the squalene bioconjugates with main cholesterol-transporting lipoproteins in rats is a generic concept driven by the squalene moiety by virtue of its bio-relation to cholesterol and is not simply dependent on the lipidic nature of the nanocarrier.

The authors would like to thank the reviewer for his/her interesting comments which have allowed to improve the manuscript.

Reviewer #3*:*

The authors have carried out and report here a study of the use of squalene to induce drug insertion into LPs for cancer cell targeting. After intravenous administration, SQ-Gem nanoparticles strongly interacted with lipopoproteins, which may facilitate active targeting due to high LP receptors expression in tumor cells. The topic is interesting. However, the whole study is not well designed and several major points were missed. The paper is premature for publication in Nature Communications. I recommend this paper should be submitted to a more specific journal. And several major concerns need to be addressed before resubmission.
1. Lack of originality innovation. The SQ-Gem conjugate and SQ-Gem NPs has been previously reported (Journal of controlled release, 2010, 147(2): 163-170; Nanomedicine: Nanotechnology, Biology and Medicine, 2011, 7(6): 841-849; Chemical Communications, 2014, 50(40): 5336-5338.). The concept of lipopoproteins involved nanoparticles for potentially targeting tumor cells cannot be considered as a novel drug delivery strategy, which is the inherent advantage of SQ-Gem conjugate and SQ-Gem NPs. This paper is a follow-up study of SQ-Gem NPs, and is more related to the pharmacokinetics of SQ-Gem NPs after intravenous administration, e.g. the distribution of SQGem and Gem in human plasma fractions.

Answer: It is the opinion of the authors that the submitted manuscript does not represent a simple additional study on SQGem NPs. We believe that this article will have a higher impact, because it demonstrates, for the first time, the use of a squalene moiety for driving drug insertion into lipoproteins, which are thus exploited as indirect natural drug carriers. Importantly, this interaction occurs spontaneously, without the need of complex LDL isolation or recombinant LDL synthesis and/or drug loading. This approach represents therefore a novel concept in drug delivery.
The additional experiments performed in this revised version of the manuscript further increase the strength of the proposed approach, as the covalent coupling to squalene of other molecules, with therapeutic or imaging activities (i.e., adenosine, NIR dye), endows the resulting bioconjugates with the capacity to interact with the lipoproteins too. The ability of squalene to mediate the insertion into lipoproteins (likely by virtue of its bio-relation to cholesterol) is unique since in additional experiments, we also demonstrated that gemcitabine-loaded liposomes (chosen as an example of another lipidic nanocarrier) did not interact with lipoproteins.
In the light of these results, we believe that we have demonstrated that the conceptual approach has a generic character, represents a real novelty in the drug delivery field and opens perspectives for a large application of the squalene-based NPs.

2. The SQ-Gem NPs and 3H-SQ-Gem NPs were not well characterized, for example, the data related to the particle size, size distribution and surface charge status are missing. How will lipopoproteins affect the nanostructure of NPs? e.g. the TEM images of SQ-Gem NPs and lipopoproteins-SQ-Gem NPs. There is only speculation, as the results of in silico simulations. Some aspects of this seem non-intuitive to me.

Answer: As suggested by the reviewer, data on the characterization of the SQGem NPs (and on the other squalene-based nanoparticles and liposomes used in this study) have been added to the revised manuscript. (See Supporting Table S1, page 43).

In addition, as suggested, we have investigated the interaction between SQGem NPs and lipoproteins by transmission electron microscopy. Briefly, SQGem NPs have been incubated with human LDL for 5 minutes at 37 °C and then images of this physical mixture have been acquired. SQGem NPs only and LDL particles only have been used as controls. In the physical mixture, shape modified SQGem NPs, possibly in the course of a disassembled process (yellow arrows), and LDL particles (pink arrows) are visible but also SQGem NPs displaying lipoproteins on their surface (blue arrows). (Supplementary Fig. S15) This microscopy image reveals how the first encounter between SQGem NPs and LDL occurs. Of note, it provides only a static image, while the interaction between the SQGem and lipoproteins is rather a dynamic phenomenon. Indeed, the establishment of this preliminary interaction is followed by the disassembly of the SQGem NPs (demonstrated by additional experiments using FRET nanoparticles, see Supplementary Fig. S12 and S13) and the release of single SQGem bioconjugate molecules that will further insert into the lipoproteins (which is obviously not observable by TEM).
These new results have been added in the revised version: Manuscript (pages 17-18); Supplementary Information (page 19); Supplementary Fig. S15.

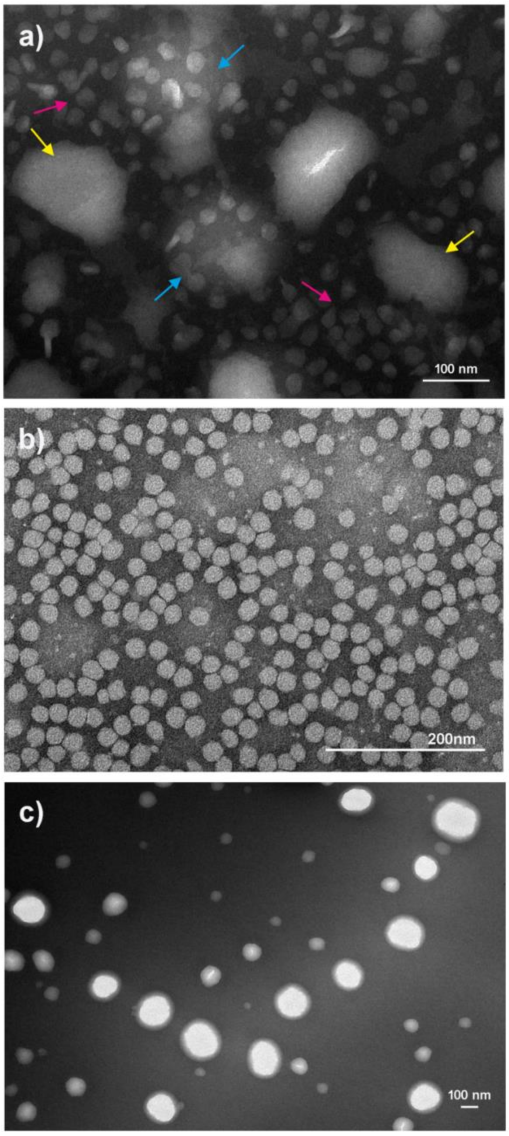

Supplementary Fig. S15. TEM images. TEM images of (a) physical mixture of SQGem NPs (final concentration 500 µg mL^{-1}) and LDL (final concentration 50 µg mL^{-1}) after 5 minutes incubation at 37°C. Yellow arrows point SQGem NPs, pink arrows point LDL particles and blue arrows point SQGem NPs with LDL particles on their surface; (b) LDL dispersion (25 ng.mL^{-1}) and (c) SQGem NPs (1 mg.mL^{-1})

To still convince the reviewer about the reality of the interaction between SQGem NPs and LDL, additional experiments have been carried out by isothermal titration calorimetry (ITC), using human lipoproteins separated from the blood of healthy volunteers. The heat flows were determined when SQGem NPs were added to LDL dispersion, HDL dispersion or albumin solution placed in the titration cell. ITC thermograms revealed the existence of a strong interaction between the SQGem and the LDL, while it was not observed either with HDL or with albumin. The specificity of the recorded signal was confirmed after dilution of SQGem NPs in PBS (Supplementary Fig. S8). These results are in agreement with those obtained after incubation of the SQGem NPs with human blood (Revised Manuscript: Figure 1, page 8) and provide a further proof of the existence of a specific interaction with the human LDL fraction, not only based on *in silico* simulations.

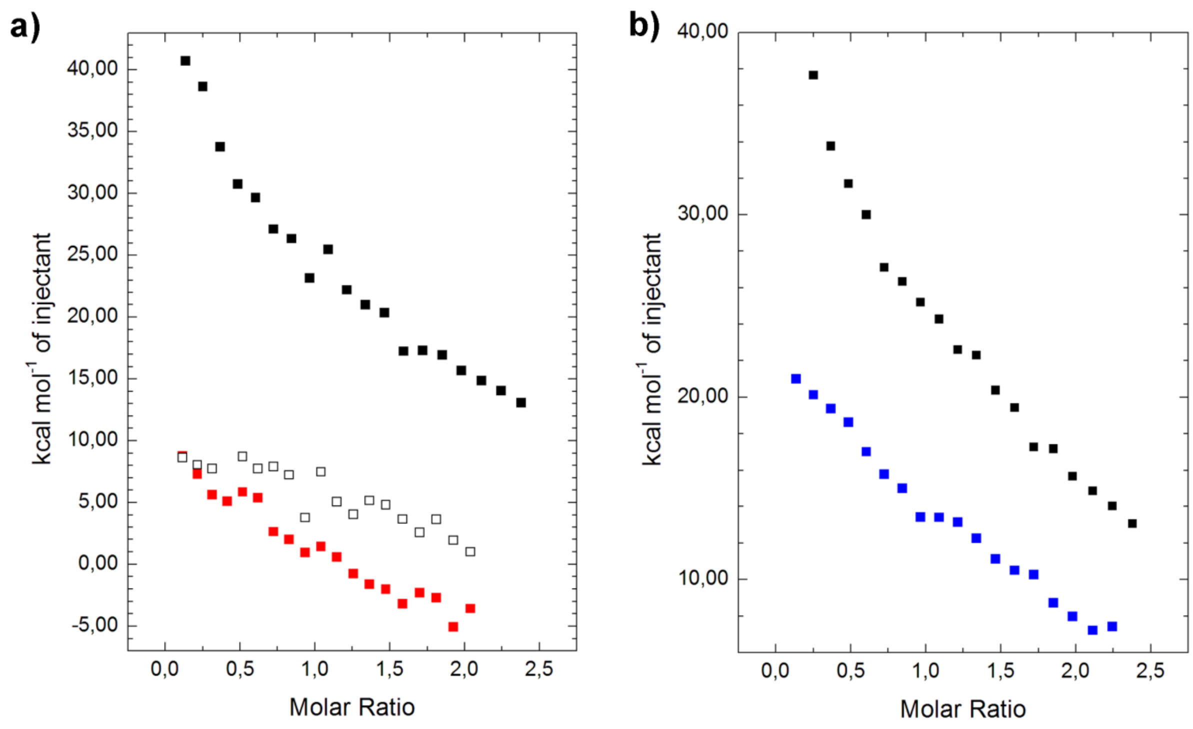

Supplementary Fig. S8. **Isothermal titration calorimetry analysis.** Isothermal titration calorimetry analysis (n=2) of the interaction between the SQGem NPs and lipoproteins or albumin. (a) Integrated binding heats upon injection of SQGem NPs into LDL (solid squares), HDL (open squares) dispersions or albumin (red squares) solution. (b) Integrated binding heats upon injection of SQGem NPs into LDL dispersion (solid squares) or PBS (blue squares).

These results have been added in the revised version: Manuscript (pages 9 and 16-17); Supplementary Information (page 18); Supplementary Fig. S8.

3. The authors concluded that squalene was chosen because of the lipid nature and its structural similarity with cholesterol, which have good affinity with lipoproteins. Whether cholesterol is a better choice to conjugate with gemcitabine?

Answer: Being the squalene a precursor in cholesterol's biosynthesis, this bio-relation drives the insertion of the SQGem bioconjugates into lipoprotein particles. We agree with the logic hypothesis of the reviewer that the replacement of the squalene by a cholesterol moiety would further increase the affinity toward lipoproteins and the transport to cells with high lipoprotein receptor activity. We had also considered this possibility but we did not follow this approach, because cholesterol-based nanomedicines may represent a cardiovascular risk factor that would compromise further possible clinical application.
In spite of this limitation, the comment of the reviewer has motivated us to verify whether a cholesterol-gemcitabine bioconjugate could self-assemble in water as nanoparticles and exert a cytotoxic activity.

Thus, we have synthesized the CholGem bioconjugate according to the following reaction scheme.

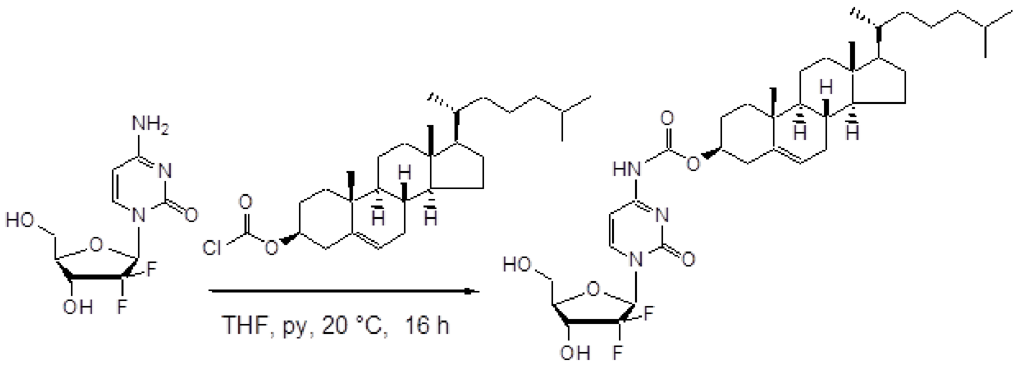

Briefly, to a suspension of gemcitabine base (99 mg, 0.37 mmol) in THF (5 mL) was added pyridine (50 mg, 0.62 mmol) and in one portion 200 mg of cholesteryl chloroformate (0.44 mmol). The resulting mixture was stirred at room temperature for 16 h and treated with aqueous 0.5 N HCl (10 mL). The mixture was then extracted with methylene chloride (3 x 15 mL). The combined extracts were washed with sodium bicarbonate (1 x 2 mL) and brine (2 x 2 mL), dried over magnesium sulfate and concentrated under reduced pressure. The crude residue was chromatographed over silica gel eluting with AcOEt/cyclohexane 2:1 to leave 190 mg of the title compound as an amorphous white solid. (75 % yield). IR (neat) ν 3500-3000 (br), 2940, 2915,1760, 1742, 1655, 1648, 1625,1549, 1440, 1432, 1407, 1365, 1344, 1315, 1285, 1265, 1259, 1210, 1192, 1158, 1131, 1117, 1103, 1080, 1059, 1026, 1015, 999, 949, 919, 827, 806, 784, 762, 722, 701, 690 cm^{-1}; ^1H NMR (300 MHz, DMSO-d6) δ 10.75 (s, 1H, N*H*), 8.20 (d, *J* = 7.5 Hz, 1H), 7.08 (d, *J* = 7.5 Hz, 1H), 6.29 (d, *J* = 6.6 Hz, 1H, OH), 6.56 (t, *J* = 7.3 Hz, 1H), 5.39 (br s, 1H), 5.27 (t, *J* = 5.2 Hz, 1H, OH), 4.55-4.40 (m, 1H), 4.25-4.05 (m, 1H), 3.95-3.75 (m, 2H), 3.70-3.55 (m, 1H), 2.50-2.25 (m, 2H), 2.00-1.70 (m, 5H), 1.65-0.90 (m, 21H), 0.99 (s, 3H), 0.89 (d, *J* = 6.3 Hz, 3H), 0.84 (d, *J* = 6.3 Hz, 6H), 0.66 (s, 3H) ppm; MS (ESI+) *m*/*z*(%) 698.3 (15) [M+Na]+, 676.3 (100) [M+H]+, 632 (10).

INSTITUT GALIEN PARIS-SUD – UMR CNRS 8612

5, rue Jean-Baptiste Clément
92296 Châtenay-Malabry cedex - France

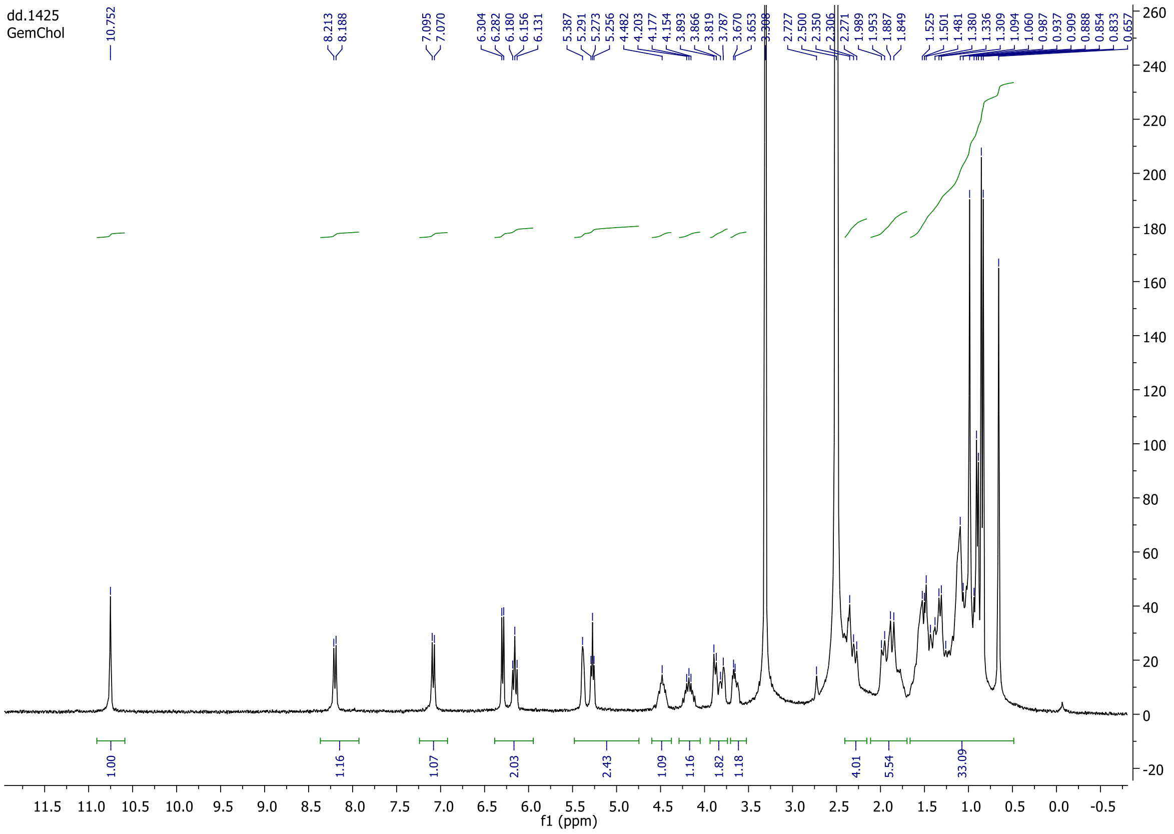

CholGem NPs were then prepared by nanoprecipitation. Briefly, CholGem was weighted and dissolved in ethanol (2 mg mL^{-1}). The resulting solution was then added dropwise under magnetic stirring into 1 mL of MilliQ® water (ethanol/water 0.5/1 v/v), followed by ethanol evaporation under vacuum. The obtained suspension contained CholGem NPs (final concentration 1 mg mL^{-1}) with a mean diameter of 134 ± 12 nm and a narrow size distribution (PdI 0.10 ± 0.02). NPs were negatively charged with a mean zeta potential value of -48 mV.

Once demonstrated that nanoparticles could be prepared, their cytotoxicity was evaluated in vitro using the 3-(4,5-dimethylthiazol-2-yl)-2,5-diphenyl tetrazolium bromide (MTT) test on MDA-MB-231 breast cancer cells and compared to that of SQGem NPs. Results are shown in the figure below. It was observed that CholGem NPs did not display any significant cytotoxicity and 70% cell viability was observed even after 72 h incubation with NPs at 200 µM concentration. On the other hand, when cells were exposed to SQGem NPs complete cell death was observed already at the concentration of 25µM.

In conclusion, although it was demonstrated that the CholGem bioconjugate was also able to self-assemble as NPs, it did not display any advantage compared to the SQGem NPs due to absence of pharmacological activity.

The design and in vitro evaluation of the CholGem NPs being out of the scope of the submitted article, these data have not been added to the revised manuscript.

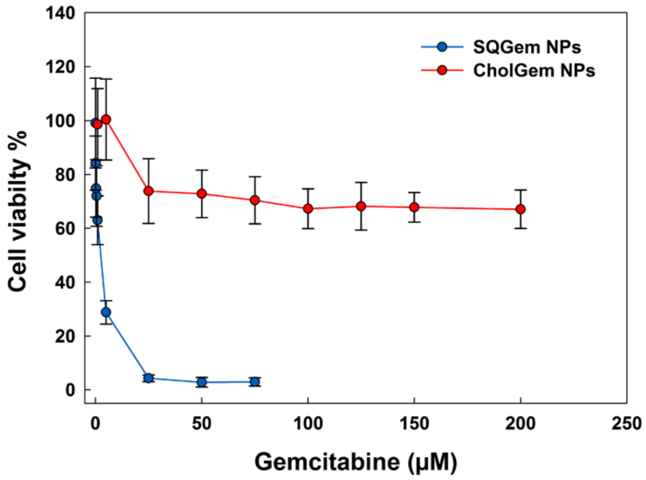

4. It is not clear to me what is meant by " In this study, a protein-driven dissociation of SQ-Gem nanoparticles into SQ-Gem monomers was demonstrated to occur before cell capture" (line 267 and 268, p 13). Please provide convincing interpretations, maybe with more evidence. If the SQ-Gem NPs were dissociated before cell uptake, how can lipopoproteins facilitate tumor cell targeting? In addition, if the SQ-Gem NPs will be dissociated in blood, what's the advantage of SQ-Gem NPs over SQ-Gem conjugates? Will the PEGylated SQ-Gem NPs also be dissociated in blood? If not, which one is a better formulation for drug delivery, PEGylated SQ-Gem NPs or non-PEGylated SQ-Gem NPs?

Answer: To meet these reviewer comments, additional experiments have been carried out in order to provide a stronger demonstration that the interaction with the lipoproteins involves single SQGem bioconjugate molecules as a consequence of the disassembly of the SQGem NPs in biological medium.

Thus, we have further investigated the disassembly of the SQGem NPs by performing new experiments with FRET SQGem NPs. These NPs have been prepared by labelling SQGem NPs with the squalene derivatives of the cyanine 5.5 (SQCy5.5, 0.6% w/w) and the cyanine 7.5 (SQCy7.5, 0.6% w/w) which behave as FRET donor and acceptor, respectively. For details concerning the preparation of these FRET nanoparticles, see Supplementary Information on pages 13-14. Fluorescence emission spectrum of these FRET NPs is reported in Supplementary Fig. S12. Taking advantage of the dependence of FRET signal from the distance between the FRET pair (SQCy5.5/SQCy7.5), this approach enabled to monitor the integrity of SQGem NPs over time.
Stability has been evaluated at 37 °C after opportune dilution of NPs in (i) water, (ii) rat blood and (iii) ethanol where SQGem is soluble. Thus, the dilution in ethanol has been used as a control of the complete disassembly of FRET SQGem NPs and consequently absence of energy transfer between the donor and acceptor dyes. Samples were imaged at regular times post incubation using the IVIS Lumina imaging system. After excitation at 640 nm (SQCy5.5 excitation wavelength), the emission was collected at 695-770 nm (donor signal, D) and 810-875 nm (FRET signal, A). Following signal quantification, the FRET proximity ratio, providing a semiquantitative measurement of the FRET efficiency, has been calculated according to the equation A/(A+D).(Preus, S. & Wilhelmsson, L. M.. *Chembiochem*, **2012**, 13, 1990-2001)

While NPs were stable in water up to 24 h, the rapid drop of the FRET signal in blood clearly indicated a fast disassembly of the NPs in this medium, thus allowing the release of individual molecules available for insertion into lipoproteins. (Supplementary Fig. S13).

These new data have been added in the revised version: Manuscript (page 14); Supplementary Information (pages 9-14 and 21); Supplementary Fig. S12 and S13.

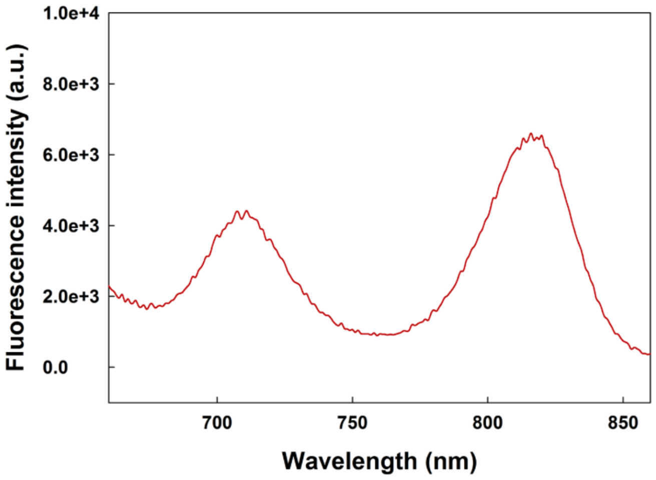

Supplementary Fig. S12: Fluorescence emission spectrum of FRET NPs. Fluorescence emission spectrum of FRET SQGem NPs, diluted in MilliQ® water (1:26.5) after excitation of the donor at 640 nm.

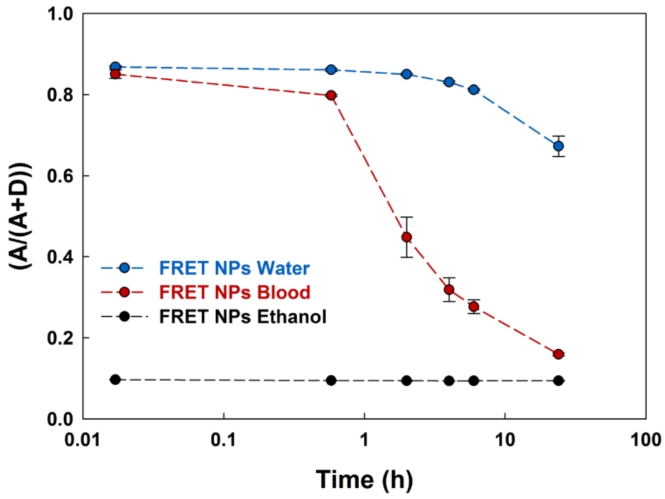

Supplementary Fig. S13. *In vitro* stability of FRET NPs. *In vitro* stability study of FRET NPs after dilution with (i) MilliQ® water, (ii) rat blood or (iii) ethanol. Results are expressed as mean ± standard error of the mean (s.e.m.) (n=2).

Now, what is the advantage of SQ-Gem NPs over SQ-Gem conjugates? Although the interaction involves single SQGem bioconjugate molecules, their formulation in form of nanoparticles is required to allow their intravenous administration *in vivo*. Indeed, the SQGem bioconjugates are soluble in organic solvents only, while they are not soluble in water, forming nanoparticles in aqueous medium. These NPs can be easily administered intravenously after adjustment of the tonicity (addition of 5% dextrose).

Which one is a better formulation for drug delivery, PEGylated SQ-Gem NPs or non-PEGylated SQ-Gem NPs?
The SQGem NPs of this study were not PEGylated. However, as pointed by the reviewer, whether the PEGylation could be responsible of a different behavior of the NPs, was worth to be investigated. Accordingly, we have prepared PEGylated SQGem NPs, and with the aim to assess their stability over time in different media, we have labelled these nanoparticles with SQCy5.5 and SQCy7.5 for allowing FRET experiments.
Briefly, 2 mg of SQGem mixed with SQPEG (10% w/w), SQCy5.5 (0.6% w/w) and SQCy7.5 (0.6% w/w) were dissolved in ethanol. The resulting solution was added dropwise under magnetical stirring into MilliQ water (ethanol/water 0.5/1). Ethanol was then evaporated under reduced pressure and a suspension of PEG_FRET NPs (final concentration of SQGem 2 mg mL^{-1}) was obtained. These nanoparticles displayed a mean diameter of 158 ± 22 nm, a narrow size distribution (PdI 0.06) and a negative surface charge (zeta potential -31 ± 5 mV).
Stability of PEG_FRET NPs was evaluated as described above for the FRET NPs. As displayed in Supplementary Fig. S14, the FRET efficiency profiles of PEGylated and non PEGylated SQGem nanoparticles perfectly overlapped. These results clearly indicated that once administered *in vivo*, the SQGem bioconjugates were released similarly from both kind of NPs (PEGylated or not) and could be further available as molecules for the interaction with circulating LPs.

On the basis of these results, it was clear that the PEGylation of the SQGem NPs would just increase the complexity of the nanoformulation, without providing any advantage in terms of drug delivery. Moreover, since several concerns about the safety of PEG have been recently reported, the possibility to achieve a long circulation time in blood without surface modification with PEG chains, but as a simple result of the spontaneous interaction with the circulating lipoproteins, represents once again an original approach.

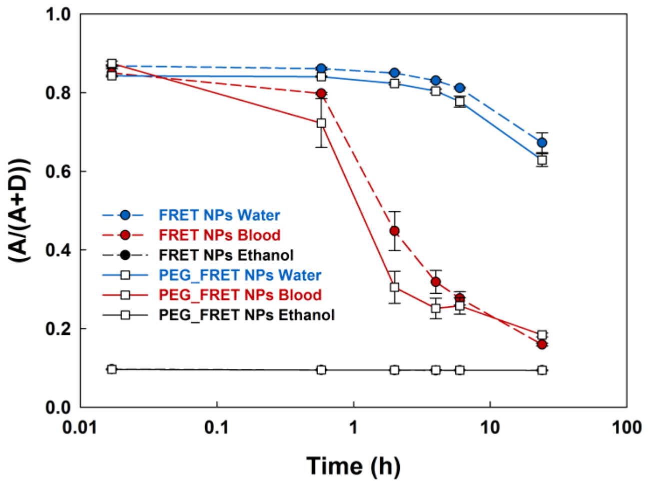

Supplementary Fig. S14. *In vitro* stability of FRET NPs vs PEG_FRET NPs. *In vitro* stability study of FRET NPs and PEG_FRET NPs after dilution with (i) MilliQ® water, (ii) rat blood or (iii) ethanol. Results are expressed as mean ± standard error of the mean (s.e.m.) (n=2).

All these new data have been added in the revised version: Manuscript (page 14); Supplementary Information (pages 9-14 and 21); Supplementary Fig. S12, S13, S14.

5. The organ distribution and the systematic pharmacokinetics profile of SQ-Gem NPs should be examined. How will lipopoproteins in vivo affect the biodistribution in organs and the pharmacokinetics of SQ-Gem NPs?

Answer: The pharmacokinetic and biodistribution of the SQGem NPs have not been included in the present manuscript because these data have been already published previously in details. (Reddy et al., Squalenoylation favorably modifies the *in vivo* pharmacokinetics and biodistribution of gemcitabine in mice, *Drug metabolism and disposition*, **2008**, 36:1570–1577). In this study, we have clearly demonstrated that the conjugation of gemcitabine to squalene deeply modified pharmacokinetic, metabolism and biodistribution profiles, comparatively to gemcitabine administered as a free drug. (See Figures below). SQGem NPs enabled blood long circulating properties with a controlled and prolonged release of gemcitabine, thus increasing the blood $t_{1/2}$ (~ 3.9-fold), the mean residence time (~ 7.5-fold) and the distribution volume compared to the drug administered as a free molecule.
On the whole, these results support the hypothesis that the interaction with the circulating lipoproteins allows the formation of a plasma reservoir of the drug. For the ease of the reviewer, we have reproduced below the pharmacokinetic and urinary excretion data, already published in Drug Metabolism and Disposition, 2008.

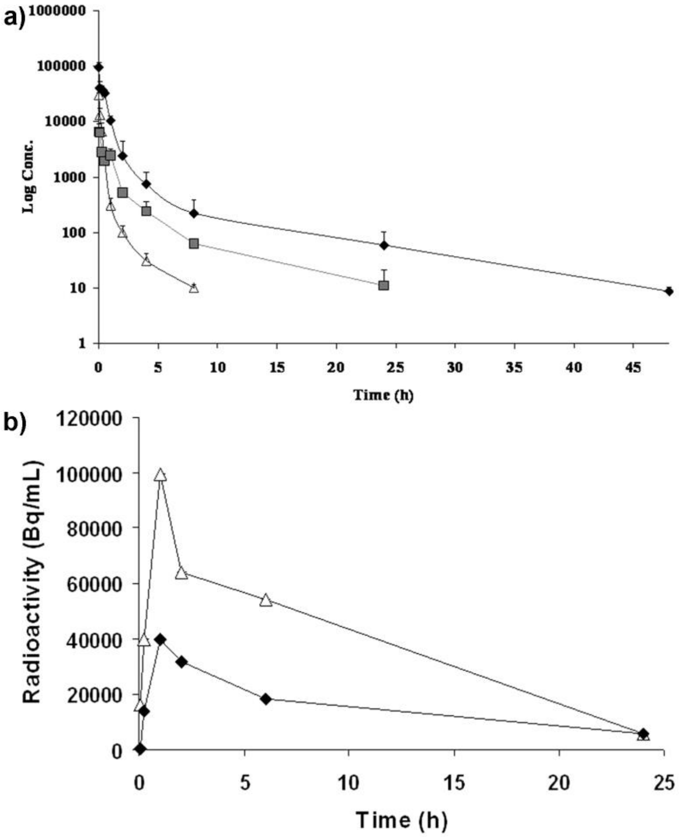

(a) Comparative plasma concentration versus time curves of Gem (△; 15 mg/kg), SQGem (◆) (15 mg/kg mol Eq. Gem), and Gem_{NPs} (■) (*i.e.*, the Gem released from SQGem NPs) in mice. Dosage was performed by LC-MS. The values are the mean ± S.D. of four mice. (b) Urinary excretion profiles of radioactivity after single-dose i.v. administration of 10 mg/kg of radiolabeled Gem (△) or SQGem NPs (◆). The values are the mean ± S.D. of three mice (from Reddy et al., *Drug metabolism and disposition*, **2008**, 36:1570–1577).

These already published data provide the reviewer with the information that the conjugation to squalene induces, indeed, a modification of the drug behavior and a different distribution pattern comparatively to the free Gem.

6. The authors conclude that "It was discovered that endogenous LDL particles may function as carriers for SQ-Gem, thus allowing the indirect targeting of cancer cells displaying high expression and activity of LDL receptors, without the need to functionalize NPs surface with hydrophilic PEG (polyethylene glycol) chains and/or with specific ligands." Actually, PEGylation cannot facilitate the cellular uptake of NPs, even hinder the cellular uptake, but benefit the long circulation in blood. Moreover, the authors didn't set the PEGylated SQ-Gem NPs as a control. How can they draw this conclusion?

Answer: As stated by the reviewer, the surface modification of nanoscale drug delivery systems with poly(ethyleneglycol) (PEG) has been successfully used to interfere with NPs opsonization and their rapid uptake. Indeed, the PEGylation endows nanoparticles with long circulating properties (ie., "stealthness") and the capacity to passively accumulate at level of the tumors, taking advantage of the so-called enhanced permeability and retention (EPR) effect. However, and we agree with the reviewer, the presence of the PEG chains could also represent an obstacle to the nanoparticle cell internalization.
Thus in the revised version of the manuscript the sentence has been rephrased as follows (see revised manuscript page 6):
"It was discovered that endogenous LDL particles may function as carriers for SQ-Gem, thus allowing the indirect targeting of cancer cells displaying high expression and activity of LDL receptors, without the need to functionalize NPs surface with specific ligands."

In the additional experiments already mentioned before, PEGylated SQGem NPs (that were missing in the first version of the manuscript) have been prepared and compared with the SQGem NPs. As described above, we have used the FRET principle to monitor the stability of these NPs both in water and in rat blood.
Results revealed the same behavior, (Supplementary Fig. S14**)** thus clearly demonstrating that both non-PEGylated and PEGylated NPs disassembled in blood at the same rate, leading to the release of individual SQGem bioconjugate molecules capable of interaction with lipoproteins. Accordingly, to meet the 3Rs guiding principles (reduction, refinement and replacement) for the use of animals in research, the PEGylated nanoparticles have not been tested *in vivo.*

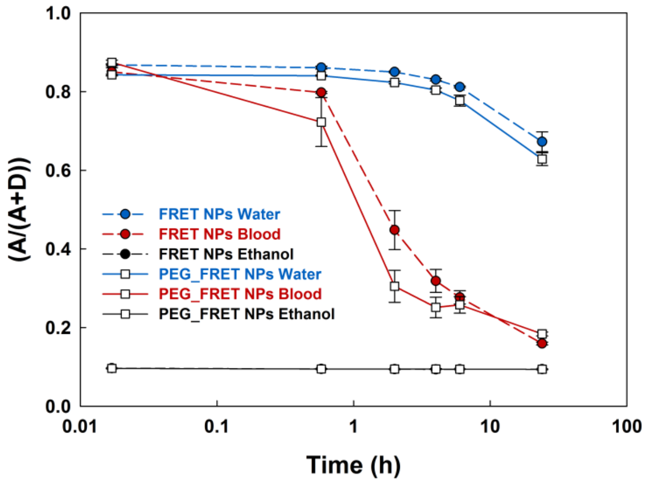

Supplementary Fig. S14. *In vitro* stability of FRET NPs vs PEG_FRET NPs. *In vitro* stability study of FRET NPs and PEG_FRET NPs after dilution with (i) MilliQ® water, (ii) rat blood or (iii) ethanol. Results are expressed as mean ± standard error of the mean (s.e.m.) (n=2).

7. The manuscript should be carefully checked for grammar mistakes.

The article has been carefully checked and mistakes have been corrected.

The authors would like to thank the reviewer for his/her comments which contributed to improve the submitted manuscript.

Reviewer #1 (Remarks to the Author):

All the doubts and criticism moved by the reviewers result addressed by an accurate work and by further experiments.

In particular the investigation of the behavior of nanoparticles made by self-assembly of two other squalene derivatives makes possible the rising in value of the claim contained in the paper title.

Reviewer #2 (Remarks to the Author):

The authors have nicely addressed my concerns and overall the paper is ready for publication.

Reviewer #3 (Remarks to the Author):

The authors added some important experiments to further support the validity and advantage of the use of squalene-based conjugates to improve the performance of GEM. Indeed, the present manuscript has been significantly improved. If the authors can address the concerns below the paper should be published in Nature Comm.

Several concerns about the PEGylation that needs to be address prior to publication:

1. Why SQPEG instead of other derivatives of PEG (e.g. the commercial available DSPE-PEG2000) was used in the PEGylated formulation? The authors might consider that SQPEG and SQGEM would have higher compatibility due to the same SQ fragment, but that is exactly the trouble. Because the authors had showed that SQ conjugates strongly interacted with lipoproteins, SQPEG would be no exception. As a result, SQPEG may do just the opposite, interacting with lipoproteins too. If so, this experiment makes no sense, but might give rise to misleading results.

2. The amount of SQPEG used in the formulation is 10% (w/w). Why? Is it enough to avoid the interaction between SQGEM NPs and lipoproteins?

3. Please specify the molecular weight of the PEG chain in SQPEG.

INSTITUT GALIEN PARIS-SUD – UMR CNRS 8612

5, rue Jean-Baptiste Clément
92296 Châtenay-Malabry cedex - France

Answers to the reviewers

Reviewers' comments:

Reviewer #1:
All the doubts and criticism moved by the reviewers result addressed by an accurate work and by further experiments. In particular the investigation of the behavior of nanoparticles made by self-assembly of two other squalene derivatives makes possible the rising in value of the claim contained in the paper title

Answer: The authors thank the reviewer for the positive comments.

Reviewer #2:$
The authors have nicely addressed my concerns and overall the paper is ready for publication.

Answer: The authors thank the reviewer for the positive comments.

Reviewer #3*:*

The authors added some important experiments to further support the validity and advantage of the use of squalene-based conjugates to improve the performance of GEM. Indeed, the present manuscript has been significantly improved. If the authors can address the concerns below the paper should be published in Nature Comm.

Answer: The authors thank the reviewer for the positive comments.

Several concerns about the PEGylation that needs to be address prior to publication:
1. Why SQPEG instead of other derivatives of PEG (e.g. the commercial available DSPE-PEG2000) was used in the PEGylated formulation? The authors might consider that SQPEG and SQGEM would have higher compatibility due to the same SQ fragment, but that is exactly the trouble. Because the authors had showed that SQ conjugates strongly interacted with lipoproteins, SQPEG would be no exception. As a result, SQPEG may do just the opposite, interacting with lipoproteins too. If so, this experiment makes no sense, but might give rise to misleading results.

Answer: We would like to recall the initial question of the reviewer: "*Will the PEGylated SQGem NPs also be dissociated in blood? If not, which one is a better formulation for drug delivery, PEGylated SQGem NPs or non-PEGylated SQGem NPs?*"
In order to answer to this question, we have prepared PEGylated SQGem NPs, and with the aim to assess their stability over time in blood, we have labelled these nanoparticles with SQCy5.5 and SQCy7.5 for performing detailed FRET experiments. On the basis of these additional experiments, we have showed unambiguously that PEGylated SQGem NPs had the same behavior than non-PEGylated SQGem NPs and that they disassembled in blood at the same rate (see figure S14). <u>As a consequence, the SQPEG bioconjugates are separated in the blood stream from the SQGem bioconjugates and therefore they do not influence the interaction of SQGem with the lipoproteins.</u>
Accordingly, it was clear that the PEGylation did not provide any advantage in terms of drug delivery, and therefore <u>we did not envision adding SQPEG to the nanoformulation, which remained composed of SQGem bioconjugates only</u>.

Now, the question of the reviewer is *why using SQPEG instead of DSPE-PEG2000*? We did perform the above mentioned experiments by using SQPEG instead of other PEG derivatives because of the excellent

compatibility of SQPEG with SQGem which has been acknowledged also by the reviewer. Importantly, the use of SQPEG for the preparation of these PEGylated SQGem NPs has been reported in details previously and this nanoformulation was certifyed in terms of colloidal properties and surface modification (see F. Bekkara-Aounallah *et al.*, Novel PEGylated nanoassemblies made of self-assembled squalenoyl nucleoside analogues, *Adv. Funct. Mater*. 2008, 18, 3715–3725).

Nevertheless, to meet the reviewer comments, we have carried out additional experiments to prepare PEGylated SQGem NPs using the DSPE-PEG 2000, instead of the SQPEG.

SQGem_DSPE-PEG NPs were prepared by nanoprecipitation. Briefly, 2 mg of SQGem mixed with DSPE-PEG (3% mol) were dissolved in ethanol. The resulting solution was added dropwise under magnetical stirring into MilliQ water (ethanol/water 0.5/1 v/v). Ethanol was then evaporated under reduced pressure and a suspension of SQGem_DSPE-PEG NPs (final concentration of SQGem 2 mg mL^{-1}) was obtained. These nanoparticles displayed a mean diameter of 114 ± 5 nm and a polydispersity value of 0.16 ± 0.01. The zeta potential value (-41 ± 3 mV) confirmed surface modification of SQGem NPs (-22 mV).
Once demonstrated that such nanoparticles could be prepared, their cytotoxicity was evaluated in vitro using the 3-(4,5- dimethylthiazol-2-yl)-2,5-diphenyl tetrazolium bromide (MTT) test on MDA-MB-231, the breast cancer cell line already used in the submitted article. And cytotoxicity of SQGem_DSPE-PEG NPs was compared to that of SQGem NPs. Results are shown in the figure below.

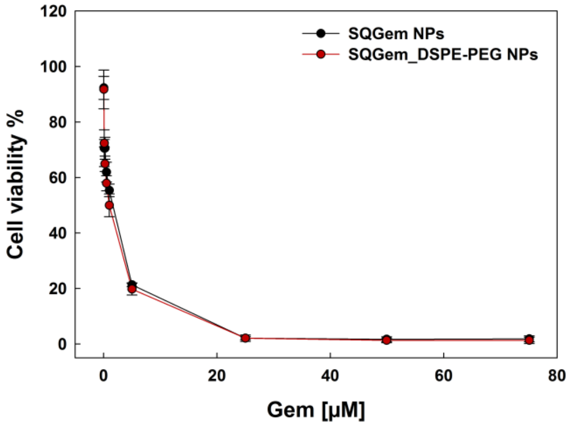

The cytotoxity profile of SQGem NPs and SQGem_DSPE-PEG NPs perfectly overlapped, thus confirming once again that the PEGylation did not modify the behavior of the SQGem NPs. Of note, the cell culture medium contained 10% serum with lipoproteins. As previously observed with SQPEG, the introduction of the DSPE-PEG didn't provide any advantage in terms of drug delivery efficiency.

Since the authors feel that the design and in vitro evaluation of the SQGem_DSPE-PEG NPs remains a little out of the scope of the submitted article, they suggest not to add these new data to the revised manuscript which could confuse the major scientific message of the paper (ie. that le linkage of the squalene moiety to a drug allows incorporation into LDL, after intravenous administration, which indirectly confers targeting capacity towards LDLR expressing cells, incl. cancer cells).

2. The amount of SQPEG used in the formulation is 10% (w/w). Why? Is it enough to avoid the interaction between SQGEM NPs and lipoproteins?

Answer: The 10 % (w/w) SQPEG in the nanoformulation corresponds to 3 mol %. This is in agreement with the PEG concentration generally used in the literature and is consistent with the general guidelines for PEGylation of nanoparticles and nanovesicles (see for exemple J. S. Suk, Q. Xu, N. Kim, J. Hanes, L. M. Ensign, PEGylation as a strategy for improving nanoparticle-based drug and gene delivery, *Adv. Drug. Deliv. Rev.* 2016, 99, 28-51 or D. Marsh, R. Bartuccib, L. Sportellib, Lipid membranes with grafted polymers: physicochemical aspects, *Biochim. Biophys. Acta – Biomembranes* 2003, 1615, 33-59).

This value (i.e., 3 mol %) has been added in the revised version of the manuscript: see Supplementary Information on page 14.

3. Please specify the molecular weight of the PEG chain in SQPEG.

Answer : The molecular weight of the PEG chain in SQPEG was 2000. PEG with this molecular weight is commonly described in literature for successfull PEGylation of nanoparticles, as demonstrated also by its use in the FDA approved Doxil® formulation, which consists of PEGylated Doxorubicin-loaded liposomes (Gabizon et al., Pharmacokinetics of Pegylated Liposomal Doxorubicin, *Clin Pharmacokinet* 2003; 42 (5): 419-436)

The PEG MW value of 2000 has been added in the revised version of the manuscript: see Supplementary Information on page 12.

Reviewer #3 (Remarks to the Author):

The paper should be published in Nature Communications.

But, please remove the SQPEG modified SQGEM NPs from the supporting information. I still think that SQPEG is not a good option for PEGylation. And the in vitro cytotoxity profile of SQGem NPs and SQGem_DSPE-PEG NPs did not say anything, in vivo evaluation is the key. BTW, 3 mol% is absolutely not the gold standard for PEGylation modification. Anyway, despite its importance, it's indeed a little out of the scope of the present study.

INSTITUT GALIEN PARIS-SUD – UMR CNRS 8612

5, rue Jean-Baptiste Clément
92296 Châtenay-Malabry cedex - France

Answers to the reviewers

Reviewers' comments:

Reviewer #3:

The paper should be published in Nature Communications.

But, please remove the SQPEG modified SQGEM NPs from the supporting information. I still think that SQPEG is not a good option for PEGylation. And the in vitro cytotoxity profile of SQGem NPs and SQGem_DSPE-PEG NPs did not say anything, in vivo evaluation is the key. BTW, 3 mol% is absolutely not the gold standard for PEGylation modification. Anyway, despite its importance, it's indeed a little out of the scope of the present study.

Answer: The authors thank the reviewer for having accepted this work to be published in Nature Communications.
As suggested by the reviewer PEGylated NPs and all related experiments have been removed because out of the scope of the study.
